# Blood cell indices and inflammation-related markers with kidney cancer risk: a large-population prospective analysis in UK Biobank

**DOI:** 10.3389/fonc.2024.1366449

**Published:** 2024-05-23

**Authors:** Qingliu He, Chengcheng Wei, Li Cao, Pu Zhang, Wei Zhuang, Fangzhen Cai

**Affiliations:** ^1^ Department of Urology, The Second Affiliated Hospital of Fujian Medical University, Quanzhou, China; ^2^ Department of Urology, Union Hospital, Tongji Medical College, Huazhong University of Science and Technology, Wuhan, Hubei, China; ^3^ Department of Urology, The First Affiliated Hospital of Chongqing Medical University, Chongqing, China; ^4^ Department of Orthopaedic, Union Hospital, Tongji Medical College, Huazhong University of Science and Technology, Wuhan, Hubei, China; ^5^ Department of Urology, Sichuan Provincial People’s Hospital, School of Medicine, University of Electronic Science and Technology of China, Chengdu, China

**Keywords:** kidney cancer, blood cell indices, inflammation-related markers, UK Biobank, prospective analysis

## Abstract

**Background:**

Kidney cancer is a prevalent malignancy with an increasing incidence worldwide. Blood cell indices and inflammation-related markers have shown huge potential as biomarkers for predicting cancer incidences, but that is not clear in kidney cancer. Our study aims to investigate the correlations of blood cell indices and inflammation-related markers with kidney cancer risk.

**Methods:**

We performed a population-based cohort prospective analysis using data from the UK Biobank. A total of 466,994 participants, free of kidney cancer at baseline, were included in the analysis. The hazard ratios (HRs) and 95% confidence intervals (CIs) for kidney cancer risk were calculated using Cox proportional hazards regression models. Restricted cubic spline models were used to investigate nonlinear longitudinal associations. Stratified analyses were used to identify high-risk populations. The results were validated through sensitivity analyses.

**Results:**

During a mean follow-up of 12.4 years, 1,710 of 466,994 participants developed kidney cancer. The Cox regression models showed that 13 blood cell indices and four inflammation-related markers were associated with kidney cancer incidence. The restricted cubic spline models showed non-linear relationships with kidney cancer. Finally, combined with stratified and sensitivity analyses, we found that the mean corpuscular hemoglobin concentration (MCHC), red blood cell distribution width (RDW), platelet distribution width (PDW), systemic immune-inflammation index (SII), and product of platelet count and neutrophil count (PPN) were related to enhanced kidney cancer risk with stable results.

**Conclusion:**

Our findings identified that three blood cell indices (MCHC, RDW, and PDW) and two inflammation-related markers (SII and PPN) were independent risk factors for the incidence of kidney cancer. These indexes may serve as potential predictors for kidney cancer and aid in the development of targeted screening strategies for at-risk individuals.

## 1 Introduction

As one of the most common urogenital malignancies, kidney cancer has garnered increased attention due to its rising incidence and significant mortality rates, accounting for 5% of all cancer cases and holding the sixth position among the most prevalent cancers in men (1–4). Kidney cancer, also known as renal cancer, is a disease that is becoming more common globally, with over 400,000 new cases reported each year. The mortality rate for kidney cancer is also high, with around 175,000 fatalities occurring annually worldwide (5–8). Despite its frequency, kidney cancer remains a complex and heterogeneous disease, characterized by challenges in early detection, limited treatment options, and an inadequate understanding of its underlying mechanisms (9). Several well-established risk factors have been identified, including age, sex, smoking, obesity, and hypertension (10, 11). However, numerous other factors that are suspected of elevating the risk of kidney cancer require further investigation (10).

Blood indices, such as the mean corpuscular volume (MCV), mean corpuscular hemoglobin (MCH), and platelet count, have served as valuable indicators of hematological alterations that could potentially reflect systemic body state and tumor progression (12–15). These biomarkers provide valuable insights into the underlying pathophysiological processes and interactions within the tumor microenvironment (16, 17). Notably, kidney cancer patients frequently exhibit anemia, with up to 35% of cases demonstrating decreased levels of hemoglobin (HGB), hematocrit (HCT), MCV, and MCH due to the weak activity of erythropoietin (EPO) and abnormal iron metabolism (18, 19). For the red blood cell and platelet systems, preoperative reductions in HGB, HCT, MCV, and MCH have been linked to an increased predisposition for early recurrence and progression of kidney cancer (20, 21). Additionally, a decrease in the average size of platelets has emerged as an independent predictor of tumor-specific mortality (18). While these findings highlight the significance of abnormal hemograms in the progression and prognosis of kidney cancer, little attention is focused on the occurrence of kidney cancer (22, 23).

Inflammation plays an important role in the incidence and progression of cancer (24, 25). Systemic inflammation is usually assessed through various biochemical or hematological markers routinely measured in common blood tests or as ratios from these measurements, which are called inflammation-related markers, including the systemic immune-inflammation index (SII), neutrophil–lymphocyte ratio (NLR), platelet–lymphocyte ratio (PLR), and product of platelet count and neutrophil count (PPN) (26–30). While increasing evidence indicates that these markers served as prognostic markers in newly diagnosed cancer patients with various malignancies, only a few studies have focused on the risk of cancer incidence (24, 31–33). As an immunogenic tumor, kidney cancer has a strongly evident interaction with inflammatory mediators (34). However, the association between inflammation-related markers and kidney cancer is still unclear.

In this paper, we aim to examine a wide range of blood cell indices and inflammation-related markers to determine their independent contributions to kidney cancer risk. Using a comprehensive prospective study design, we analyzed a large dataset comprising kidney cancer patients and matched controls, thereby providing valuable indexes into the development of this malignancy.

## 2 Methods

### 2.1 Study design

The UK Biobank is a large-scale prospective study designed to provide valuable resources for investigating the causes of various diseases. Comprehensive information regarding the study procedure and data collection can be accessed online or through relevant literature sources (https://www.ukbiobank.ac.uk/media/gnkeyh2q/study-rationale.pdf) (35). In brief, the study included participants who were registered with the NHS and lived within a 40-km radius of a UK Biobank assessment center. An initial invitation was extended to approximately 9.2 million individuals (36). Between 2006 and 2010, over 500,000 men and women aged between 40 and 69 years old consented to participate in the cohort and visited one of the 22 assessment centers located in England, Wales, and Scotland. The UK Biobank study was approved by the National Health and Social Care Information Management Board and the North West Multicenter Research Ethics Committee (11/NW/0382). This approval ensures that the study was conducted in compliance with ethical guidelines and that the rights and privacy of the participants were protected.

### 2.2 Population selection

**Figure 1:**
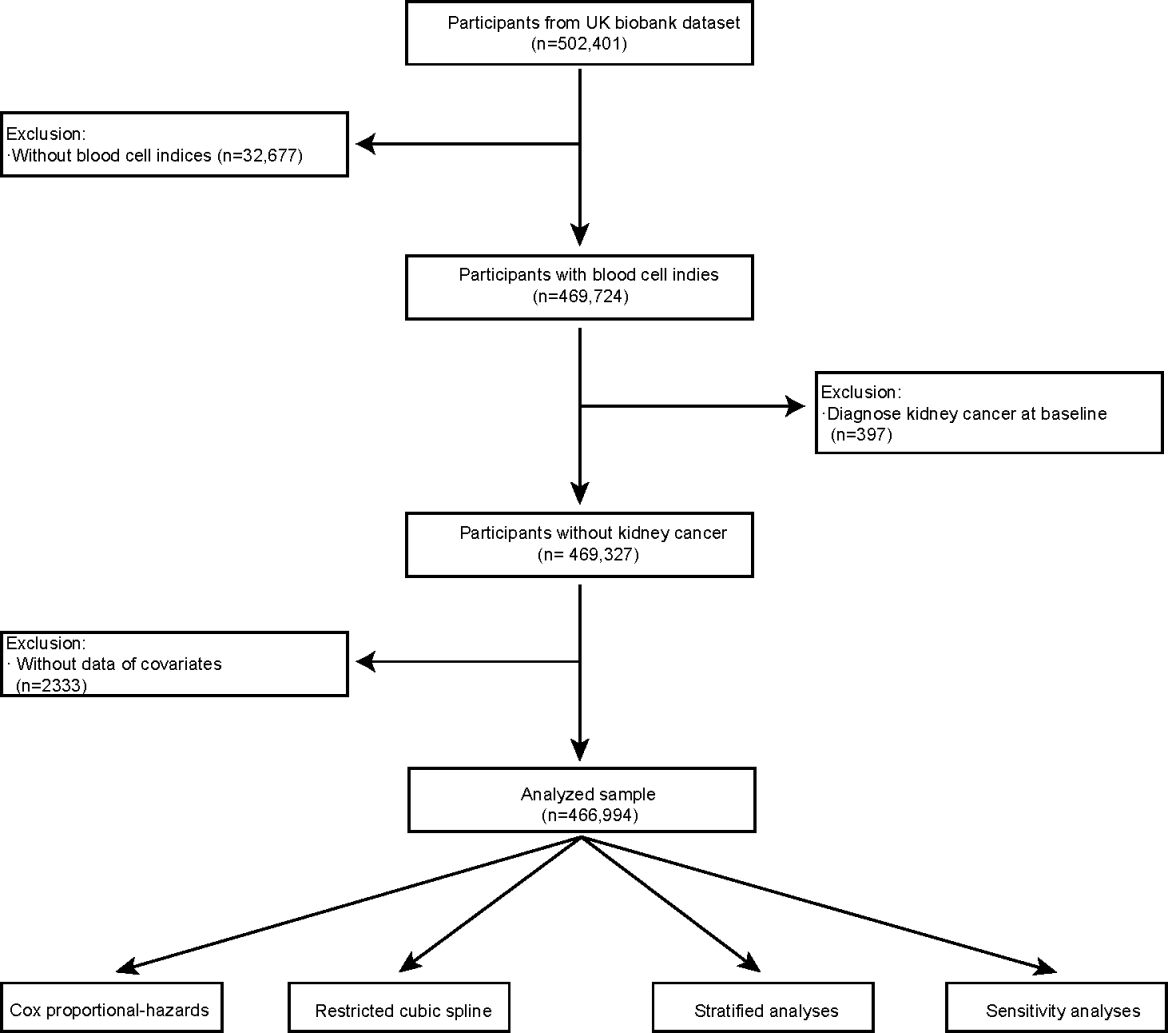
Flowchart of the research design.

This study was performed following the guidelines and regulations outlined in UKB application number 61083. A total of 502,401 participants were included in our analysis. Initially, we excluded 32,677 participants who had missing blood cell indices. Subsequently, we eliminated 397 participants who had kidney cancer at baseline and were lacking critical covariate data (*N* = 482). Ultimately, the primary analysis consisted of 466,994 participants (**Figure 1**).

### 2.3 Blood cell indices

UKB comprises a comprehensive collection of blood cell indices extracted from biological samples. Using a hematology analyzer, exclusive count data were acquired, and other parameters were calculated accordingly (details at https://biobank.ctsu.ox.ac.uk/crystal/ukb/docs/haematology.pdf). All indices included in this study were extracted from the initial visit and then classified as “red blood cell,” “immature red blood cell,” “white blood cell,” and “inflammation-related index.” The red blood cell category included RBC, HCT, MCV, MCH, MCHC, and RDW. The immature RBC category included the reticulocyte count, reticulocyte percentage, nucleated RBC, nucleated RBC percentage, MRV, MSCV, and IRF. The white blood cell category included WBC, basophil count (BASO), basophil percentage, eosinophil count (EO), eosinophil percentage, monocyte count, monocyte percentage, neutrophil count (NEUT), neutrophil percentage, lymphocyte count (LYMPH), lymphocyte percentage, MPV, platelet distribution width, and platelet (PLT) and high light scatter reticulocyte percentage. For a more complete examination of the connection between “inflammation-related marker” and the incidence of kidney cancer, SII, PLR, NLR, and PPN were calculated. PLT, NEUT, and LYMPH concentrations were measured at 1,000 cells/µL. PLT was multiplied by (NEUT/LYMPH) to arrive at SII. The PLR was computed by dividing the PLT by the LYMPH. NLR was determined using NEUT/LYMPH. PPN was determined by multiplying PLT by NEUT. All analytes within the dataset had identical detection limits. None of the results fell below the threshold for detection. **Supplementary Table S1** of the **Supplementary Materials** shows a list of indicators with their corresponding abbreviations and data fields.

### 2.4 Covariates

We utilized the baseline touch-screen questionnaire to evaluate a number of potential confounding variables: age (years), sex, race (white, mixed, Asian, black, etc.), household income, lifestyle behavior (smoking status and alcohol drinker status), and body mass index (BMI). The questionnaire yielded the codes a degree (college or university degree) or no degree for education. The Townsend Deprivation Index is a composite measure of deprivation based on nonhome ownership, non-car ownership, unemployment, and domestic overcrowding that indicates the socioeconomic status of the participant. Adapted from the American Heart Association Guidelines, a balanced diet score was defined as adhering to four or five of the following components: (1) total fruit consumption of 4.5 pieces per week, (2) total vegetable consumption of 4.5 servings per week (three tablespoons of vegetable considered as one serving), (3) total fish consumption of two servings per week, (4) processed meat consumption at two times per week, and (5) red meat consumption at five times per week. The comorbidity variables included hypertension (Field 6150) and diabetes (Field 2443).

### 2.5 Kidney cancer diagnosis

This study’s results were C64 (kidney malignant tumor, excluding renal pelvis). The International Classification of Diseases (ICD) classification system was utilized to record the diagnoses. The incident disease in this study was determined by the primary or secondary diagnoses from hospital admission data or by the primary or secondary causes after the baseline data collection. The participants were observed from the time of baseline until the date of the first diagnosis or on December 31, 2021.

### 2.6 Statistical analysis

The mean (standard deviation, SD) and number (percentage) for continuous variables were obtained. **Figure 1** illustrates the study design flowchart. Blood cell indices were log-transformed and normalized to *Z* scores [*Z* = (value - mean)/SD] prior to Cox analysis so that the hazard ratio (HR) represents the effect per SD increment. Model 1 represented the unadjusted model, which did not consider any covariates. Model 2, the minimally adjusted model, accounted for gender and age as potential confounding variables. Model 3, the fully adjusted model, considered a comprehensive set of covariates including sex, age, race, qualification, BMI, smoking, alcohol consumption, health diet score, household income, diabetes, and hypertension. The potential for nonlinearity was investigated using restricted cubic spline models fitted to Cox models and adjusted for covariates as in model 3. Potential nonlinearity was examined by comparing the linear model to the model containing both linear and cubic spline terms using the likelihood ratio test. Then, we performed stratified analyses to estimate the potential modification effects according to age (≤60 and >60 years), sex (male or female), race (white, mixed, Asian, black, and others), smoking status (current, former, and never), alcohol drinker status (current, former, and never), qualifications (with college or university degree or none), diabetes (yes and no), hypertension (yes and no), and BMI (≤25 and >25). By modeling the cross-product terms of the stratifying variables with each blood cell index, interaction *P* values were examined. In the sensitivity analysis, we first applied models 1 and 2 to test the robustness of our findings. Second, we used quantile regression for further analysis. Third, we used the first repeat assessment visit of blood cell index data in 2012 and conducted Cox regression analysis to support the stability of the results.

All statistical analyses were carried out using R version 4.2.1. All *P* values listed below were adjusted.

## 3 Results

### 3.1 Baseline characteristics

Baseline characteristics stratified by kidney cancer status are shown in **Table 1**. A total of 466,994 kidney cancer-free participants from UKB were included in the primary analyses. Overall, the mean age of the participants was 56.5 (± 8.1) years; 214,089 (45.8%) were male patients and 252,905 (54.2%) were female patients. During a mean follow-up time of 12.4 years, 1,710 participants developed kidney cancer. Among them, 1,094 (64.0%) participants were male patients, and 616 (36.0%) participants were female patients.

**Table 1:** Baseline characteristics of UK Biobank participants by incident kidney cancer incidence.

Characteristics	Overall (*N* = 466,994)	No kidney cancer (*N* = 465,284)	Kidney cancer (*N* =1,710)	*P* value
**Mean follow-up duration (years) (SD)**	12.4 ± 1.0	12.4 ± 0.9	6.5 ± 3.5	<0.001
**Age, mean (SD), years**	56.5 ± 8.1	56.5 ± 8.1	60.6 ± 6.5	<0.001
**Gender (%)**				<0.001
Male	214 089 (45.8)	212.995 (45.8)	1,094 (64.0)	
Female	252,905 (54.2)	252,289 (54.2)	616 (36.0)	
**Race (%)**				<0.001
White European	44,1207 (94.5)	439,551 (94.5)	1,656 (96.8)	
Mixed	2,717 (0.6)	2,711 (0.6)	6 (0.4)	
South Asian	8,798 (1.9)	8,777 (1.9)	21 (1.2)	
Black	7,073 (1.5)	7,061 (1.5)	12 (0.7)	
Others	7,199 (1.5)	7,184 (1.5)	15 (0.9)	
**With college or university degree (%)**	151,108 (32.4)	150,686 (32.4)	422 (24.7)	<0.001
**BMI, mean (SD), kg/m^2^**	27.4 ± 4.8	27.4 ± 4.8	28.9 ± 4.9	<0.001
**Smoking status (%)**				<0.001
Current	48,996 (10.5)	48,757 (10.5)	239 (14.0)	
Former	161,651 (34.6)	160,930 (34.6)	721 (42.2)	
Never	254,506 (54.5)	253,763 (54.5)	743 (43.5)	
Missing	1,841 (0.4)	1,834 (0.4)	7 (0.4)	
**Alcohol drinker status (%)**				0.001
Current	429,274 (91.9)	427,711 (91.9)	1,563 (91.4)	
Former	16,682 (3.6)	16,604 (3.6)	78 (4.6)	
Never	20,387 (4.4)	20,325 (4.4)	62 (3.6)	
Missing	651 (0.1)	644 (0.1)	7 (0.4)	
**Health diet score, mean (SD)**	2.2 ± 0.9	2.2 ± 0.9	2.1 ± 0.9	<0.001
**Household income (%)**				<0.001
<18,000 (£)	19,707 (4.2)	19,638 (4.2)	69 (4.0)	
18,000–30,999 (£)	138,248 (29.6)	137,642 (29.6)	606 (35.4)	
31,000–51,999 (£)	101,721 (21.8)	101,297 (21.8)	424 (24.8)	
52,000–100,000 (£)	104,384 (22.4)	104,039 (22.4)	345 (20.2)	
>100,000 (£)	81,350 (17.4)	81,125 (17.4)	225 (13.2)	
“Do not know” or missing	21,584 (4.6)	21,543 (4.6)	41 (2.4)	
Health status (%)				
Diabetes history	24,210 (5.18%)	24,053 (5.17%)	157 (9.18%)	<0.001
Hypertension	111,708 (23.92%)	111,055 (23.87%)	653 (38.19%)	<0.001

For continuous variables, data are presented as mean (SD), and for categorical variables, data are presented as number (percentage). The level of education was classified as either higher (college/university degree) or lower.

### 3.2 Blood cell indices and kidney cancer risk

**Figure 2:**
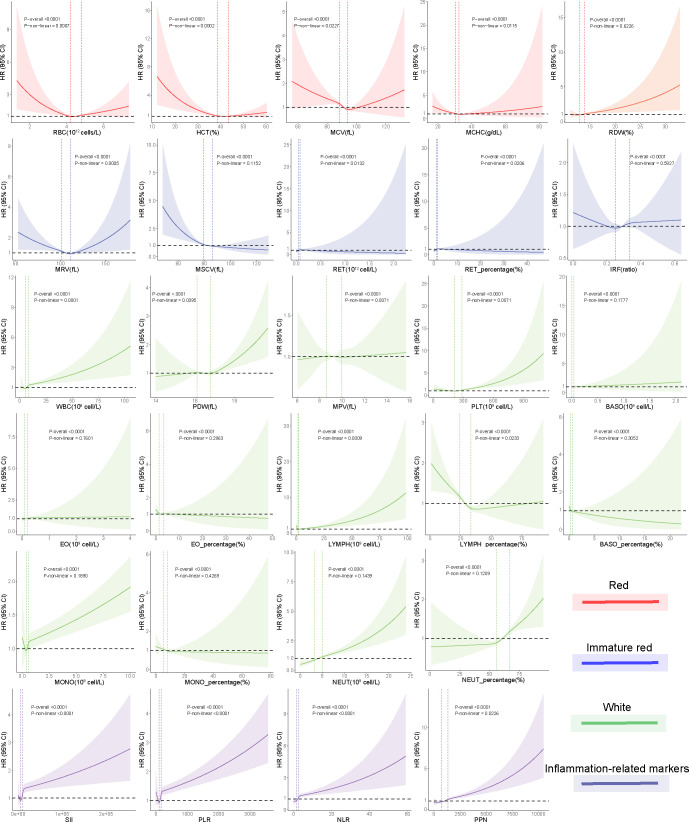
Nonlinear associations between blood cell indices and risk of incident kidney cancer using restricted cubic spline models. Two vertical dashed lines represent the 25% and 75% exposure values for each exposure. Adjustments were made for demographic and socioeconomic factors. The red, green, blue, and purple hues denote that each blood cell measurement corresponds to the “red blood cell,” “immature red blood cell,” “white blood cell,” and inflammation-related index categories, respectively.

In the RBC category, decreased levels of MCV [HR 0.90, 95% confidence interval (CI): 0.86–0.95, *P* < 0.0001] and MCHC (HR 0.91, 95% CI: 0.876–0.96, *P* < 0.0006), indicative of an anemia state, were associated with higher kidney cancer incidence in the fully adjusted models (model 3). RDW, indicative of the heterogeneity of erythrocytes, was positively associated with kidney cancer risk (HR 1.10, 95% CI: 1.05–1.14, *P* < 0.0001). U-shaped relationships were discovered between RBC (*P* for nonlinearity = 0.0007; **Figure 2**), HCT (*P* for nonlinearity <0.0002; **Figure 2**), MCV (*P* for nonlinearity = 0.0227; **Figure 2**), and MCHC (*P* for nonlinearity = 0.0115; **Figure 2**) with incident kidney cancer.

In the immature RBC category, decreased reticulocyte maturation parameters, including NRBC (HR 0.88, 95% CI: 0.78–0.99, *P* = 0.0391), NRBC% (HR 0.88, 95% CI: 0.78–0.99, *P* = 0.0391), MRV (HR 0.94, 95% CI: 0.90–0.99, *P* = 0.0180), and MSCV (HR 0.88, 95% CI: 0.83–0.92, *P* < 0.0001), were significantly associated with a higher kidney cancer risk. MRV (*P* for nonlinearity = 0.00051) had a U-shaped association with kidney cancer risk. MSCV had a linear association with kidney cancer risk, rapidly reaching the lowest risk at ≈80 and becoming flat thereafter.

In the WBC category, increased levels of WBC (HR 1.04, 95% CI: 1.03–1.05, *P* < 0.0001), NEUT (HR 1.14, 95% CI: 1.11–1.17, *P* < 0.0001), and NEUT% (HR 1.17, 95% CI: 1.12–1.23, *P* < 0.0001) trended toward a higher kidney cancer risk, while increased levels of LYMPH% (HR 0.85, 95% CI: 0.81–0.89, *P* < 0.0001) showed protective effects against incident kidney cancer. Among platelet indices, we found significant associations between kidney cancer incidence and PDW (HR 1.07, 95% CI: 1.02–1.12, *P* = 0.0054) and PLT (HR 1.12, 95% CI: 1.09–1.15, *P* < 0.001). U-shaped associations were also found between PLT (*P* for nonlinearity < 0.0001; **Figure 2**) and kidney cancer risk.

### 3.3 Inflammation-related markers and kidney cancer risk

**Figure 3:**
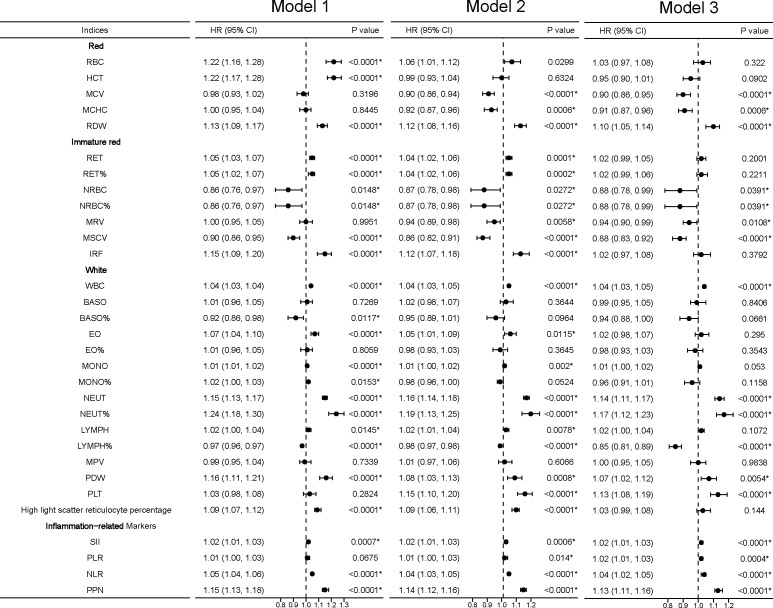
Linear associations between blood cell indices and incident kidney cancer. *Model 1 was a non-adjusted model that adjusted for none. **Model 2 was a minimally adjusted model adjusted for gender and age. ***Model 3 was a fully adjusted model adjusted for gender, age, race, qualification, BMI, smoking, alcohol, health diet score, household income, diabetes, and hypertension. Exposures (excluding the inflammation-related markers) were log-transformed and standardized to the Z score so that the HR represents the predicted effect of a one-SD increment. Statistical significance at *P* < 0.05.

We found that white blood indices were significantly positively associated with the incidence of kidney cancer. Thus, we hypothesize that inflammation status may be associated with kidney cancer. We developed four inflammation-related markers, including the systemic immune-inflammation index (SII), platelet-to-lymphocyte ratio (PLR), neutrophil-to-lymphocyte ratio (NLR), and the product of platelet count and neutrophil count (PPN). In the inflammation-related index category, increased SII (HR 1.02, 95% CI: 1.01–1.03, *P* < 0.0001), PLR (HR 1.02, 95% CI: 1.01–1.03, *P* < 0.0001), NLR (HR 1.04, 95% CI: 1.02–1.05, *P* < 0.0001), and PPN (HR 1.13, 95% CI: 1.11–1.16, *P* < 0.0001) trended toward a higher kidney cancer risk (**Figure 3**). U-shaped associations were also found between PLT (*P* for nonlinearity <0.0001; **Figure 2**) and kidney cancer risk. RCS models revealed a positive association between the inflammation-related index and kidney cancer risk (**Figure 2**). Then, we conducted quantile regression between the inflammation-related markers and incident kidney cancer. We divided the inflammation-related index into four quartiles (Q1–Q4) (**Table 2**). We found that Q4 had a higher incidence of kidney cancer in all models, while *P* for trend was significantly different in model 3. In Q4, SII (HR 1.47, 95% CI: 1.29–1.68, *P* < 0.0001), PLR (HR 1.44, 95% CI: 1.26–1.64, *P* < 0.0001), NLR (HR 1.57, 95% CI: 1.36–1.81, *P* < 0.0001), and PPN (HR 1.55, 95% CI: 1.35–1.78, *P* < 0.0001) trended toward a higher kidney cancer risk compared with Q1 in model 3 (**Table 2**).

**Table 2:** Quantile regression between inflammation-related markers and incident kidney cancer.

Inflammation-related index	Model 1 HR (95% CI)	Model 2 HR (95% CI)	Model 3 HR (95% CI)
Systemic immune-inflammation index (SII)			
Q1	Reference		
Q2	0.97 (0.85, 1.12)	1.02 (0.89, 1.17)	1.06 (0.92, 1.22)
Q3	0.94 (0.82, 1.08)	1.01 (0.88, 1.16)	1.08 (0.93, 1.24)
Q4	1.26 (1.11, 1.43)	1.33 (1.17, 1.52)	1.47 (1.29, 1.68)
P trend	<0.001	<0.001	<0.001
Platelet-to-lymphocyte ratio (PLR)			
Q1	Reference		
Q2	0.96 (0.84, 1.10)	1.03 (0.90, 1.18)	1.10 (0.96, 1.26)
Q3	0.93 (0.81, 1.07)	1.03 (0.90, 1.18)	1.14 (0.99, 1.31)
Q4	1.13 (0.99, 1.29)	1.25 (1.10, 1.43)	1.44 (1.26, 1.64)
P trend	0.068	<0.001	<0.001
Neutrophil-to-lymphocyte ratio (NLR)			
Q1	Reference		
Q2	1.22 (1.05, 1.42)	1.17 (1.00, 1.36)	1.14 (0.98, 1.33)
Q3	1.46 (1.26, 1.69)	1.34 (1.16, 1.55)	1.30 (1.12, 1.51)
Q4	1.92 (1.67, 2.20)	1.61 (1.40, 1.86)	1.57 (1.36, 1.81)
P trend	<0.001	<0.001	<0.001
Product of platelet count and neutrophil count (PPN)			
Q1	Reference		
Q2	1.12 (0.96, 1.30)	1.13 (0.98, 1.31)	1.08 (0.93, 1.25)
Q3	1.34 (1.17, 1.55)	1.40 (1.22, 1.61)	1.29 (1.12, 1.48)
Q4	1.62 (1.41, 1.86)	1.80 (1.57, 2.06)	1.55 (1.35, 1.78)
*P* trend	<0.001	<0.001	<0.001

Model 1 was a non-adjusted model that adjusted for none. Model 2 was a minimally adjusted model adjusted for gender and age. Model 3 was a fully adjusted model adjusted for gender, age, race, qualification, BMI, smoking, alcohol, health diet score, household income, diabetes, and hypertension.

### 3.4 Stratified analyses

Based on possible effect modifiers and blood cell indices risk variables, HRs in stratified analyses were often in the same direction. In the RBC category, we found that the associations of MCV, MCHC, and RDW were stronger among older, male, white European, former smoker, current alcohol drinker, people with college or university degree, no diabetes, hypertension, and BMI >25 participants. In the immature red category, MSCV and MRV showed significant associations among the above-mentioned specific groups, while NRBC and IRF were not remarkable. In the WBC category, we found that associations of WBC, NEUT, NEUT%, LYMPH%, and PDW had similar trends in both age and gender, while PLT had stronger associations in the older participants. For the inflammation-related index category, SII, PLR, NLR, and PPN had similar results to the RBC category (**Supplementary Tables S2**–**S5**).

### 3.5 Sensitivity analyses

**Figure 4:**
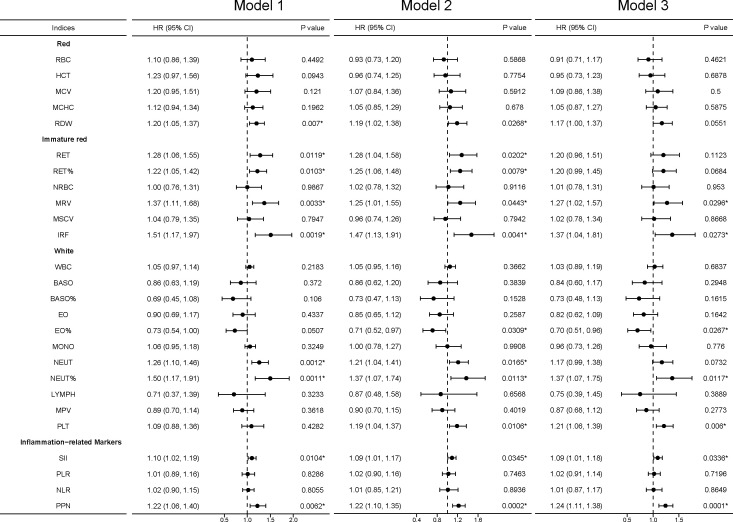
Sensitivity analyses between blood cell indices and incident kidney cancer. Model 1 was a non-adjusted model that adjusted for none. Model 2 was a minimally adjusted model adjusted for gender and age. Model 3 was a fully adjusted model adjusted for gender, age, race, qualification, BMI, smoking, alcohol, health diet score, household income, diabetes, and hypertension. Exposures (excluding inflammation-related markers) were log-transformed and standardized to the Z score so that the HR represents the predicted effect of a one-SD increment. Statistical significance at *, P < 0.05.

In sensitivity analyses, we found that the association between the RBC category and kidney cancer risk lost significance in model 3, including MCV, MCHC, and RDW, while the association between RDW and kidney cancer risk remained significant in model 1 and model 2. For the immature RBC category, MRV maintained a significant difference and IRF regained a significant difference in model 3. For the WBC category, EO%, NEUT%, and PLT were significantly associated with kidney cancer risk. The inflammation-related markers SII and PPN had a stable association (**Figure 4**).

## 4 Discussion

To the best of our knowledge, this is a comprehensive prospective investigation of the connection between 27 blood cell indices and incident kidney cancer. The study utilized data from a substantial cohort of 466,994 participants from the UK Biobank, with an average follow-up period of 12.4 years. We confirmed that three blood cell indices (MCHC, RDW, and PDW) and two inflammation-related markers (SII and PPN) were associated with the risk of kidney cancer. However, further investigation is needed to elucidate the underlying mechanisms responsible for these associations. In the future, we can predict tumor risk by building disease prediction models based on blood cells. At the same time, it provides another strategy for population screening, and further detection of outliers in the blood cell index population could exclude the risk of tumor occurrence. Finally, our research can guide the direction of future basic research. Various exposure factors may promote the development or progression of tumors by mediating the circulation of blood cells.

The detection, classification, and function of circulating immune cells are helpful to further understand the complex mechanism of tumors. The changes in hematological indexes reflect the changes of the internal environment in the body, which may provide necessary conditions for the occurrence and development of tumors. Hypoxia plays an important role in cancer progression. Dynamin-related protein 1 (Drp1) was an important protein that controls the quality of mitochondria and cellular processes through alterations in its oligomeric structure and other modifications that may be associated with hematological index changes (37). Single-cell RNA sequencing could provide a new insight into the tumor immune microenvironment (38, 39). Emerging nano-/biotechnology could drive oncolytic virus-activated and combined cancer immunotherapy through hematological mediation, and some supramolecular biomaterials could also be applied for cancer immunotherapy (40–43). At present, more and more detection techniques based on multi-omics have played an important role in the diagnosis, treatment, and identification of tumors. Our study highlights the possible role of immune-associated cells in the development of kidney cancer.

It is widely reported that MCHC plays an important role in regulating the development and progression of several cancers, such as non-small cell lung cancer (44), prostate cancer (45), oral squamous cell carcinoma, and head and neck cancers (46). We found that a decreased level of MCHC was significantly correlated with a higher kidney cancer incidence at first. MCHC is an important index of anemia, and anemia is hypothesized to be an independent adverse prognostic indicator in patients with kidney cancer (20, 47). Behind anemia, the accompanying hypoxia may play a critical role. Hypoxia, a condition in which tissues are oxygen-deprived, upregulates the expression of hypoxia-inducible factor (HIF), which then induces hundreds of genes in an HIF-dependent manner to encode proteins that play key roles in numerous aspects of cancer biology, such as proliferation, cell survival, epithelial-to-mesenchymal transition (EMT), angiogenesis, invasion, and metastasis (48). Consequently, we infer that MCHC impacts kidney cancer risk, possibly through anemia and HIF pathways (49).

RDW, which reflects erythrocyte volume and size heterogeneity, is regarded as a strong and independent risk factor for death in the general population and is expected to be an effective indicator (50). In addition, it is generally recognized that RDW is a risk factor for various diseases, such as cardiovascular disease, venous thromboembolism, diabetes, and so on (50). Actually, we found that RDW was positively associated with kidney cancer risk, which supports RDW as a potential diagnosis marker for kidney cancer.

Platelets have been reported to influence cancer progression, metastasis, and angiogenesis in multiple ways, such as protecting tumor cells from high shear forces in the blood circulation and leukocyte attack and binding to C-type lectin-like receptor 2 (CLEC-2) to facilitate hematogenous cancer metastasis and cancer-associated thrombosis (12, 51). It is well documented that a higher platelet count is linked with shorter disease-specific survival in kidney cancer (52). PDW reflects the variation of platelet volume in the blood. The results demonstrated that elevated PDW is linked with a high kidney cancer incidence, which demonstrated that PDW might be an effective risk factor for kidney cancer.

While previous research discovered that the inflammation marker SII was connected with kidney cancer risk (53), the association of PPN and kidney cancer has not been clear. Our paper showed that both SII and PPN were related to kidney cancer risk. Inflammatory conditions can exist in some types of cancer prior to the development of a malignant transformation. In other types of cancer, an oncogenic alteration causes an inflammatory microenvironment which improves cancer cell growth (54, 55). However, inflammatory conditions can exist in some types of cancer prior to the development to a malignant transformation, and chronic inflammation is generally acknowledged as an independent risk factor for the development of a majority of cancers (56). Another study suggests that inflammatory pathways can promote kidney cancer cell growth and immune evasion (34), which may provide theoretical bases for inflammation markers acting as risk factors for kidney cancer.

Several studies have shown a strong association between SII and kidney cancer. A study showed that SIRI and SII indexes show a moderate efficiency to show metastases in RCC (57). A large multi-center longitudinal study showed that SII increases the risk of total and cause-specific mortality among patients with chronic kidney disease, including kidney cancer (58). SII could also predict the survival of patients with renal cell cancer treated with nivolumab (59). Now, most articles have studied the relationship between SII and renal cancer progression and response to treatment. However, the incidence of kidney cancer and inflammation-related markers still requires further research.

There are several advantages to this study. First, this is a comprehensive examination of the association between blood indices and incident renal cancer. Second, owing to the large sample size, lengthy follow-up period, and extensive measurement of covariates, our study is longitudinal and possesses distinct advantages. Third, we summarized the mechanisms underpinning the association between blood indices and kidney cancer, which provides strong evidence for clinical application.

There are inevitably some limitations and deficiencies. Because these blood indices may be associated with health status and are susceptible to other factors such as comorbidities, socioeconomic status, and poor health, it is not feasible for this analysis to rule out detection bias. Then, the identification of UKB is determined by the ICD classification. Regrettably, the ICD categorization system relies on morphology rather than pathology, making it impossible to differentiate the relative risk of various blood cells for different forms of kidney cancer based on their pathological characteristics. In addition, the majority of UKB participants are European, which necessitated the use of stratified analyses that limited the generalizability of our results.

Additional limitations can be due to the fact that the pathogenesis of renal cancer is still unclear, and many factors influence blood cell indices. Hypertension and diabetes were the main risk factors of kidney cancer that could affect blood cell indices. Cigarette smoking and obesity are also likely to affect kidney cancer incidence trends and also included in the covariates (60–62). However, inadequate adjustment of covariates may lead to a bias in this study. Thus, establishing a larger cohort associated with renal cancer will help further explore the role of circulating immune cells. The simultaneous use of high-throughput sequencing and more detailed case data could also help further understand the specific mechanisms of kidney cancer progression. Establishing a kidney cancer-related cohort based on a diverse population is necessary to improve generalizability.

## 5 Conclusion

This study identified three blood cell indices and two inflammation-related markers as dependent risk factors for kidney cancer incidence. As these indexes could be obtained through routine blood tests, they would be useful in large-scale screening or primary care settings to help discover individuals who might benefit from early screening or targeted prevention strategies for kidney cancer. Additional research is necessary to further illustrate the underlying mechanisms by which these blood cell indices and inflammatory-related markers are associated with kidney cancer risk.

## Data availability statement

The original contributions presented in the study are included in the article/**Supplementary Material**. Further inquiries can be directed to the corresponding authors.

## Ethics statement

This research was conducted under the UK Biobank application 61083. The UK Biobank study was approved by the National Health and Social Care Information Management Board and the North West Multicenter Research Ethics Committee (11/NW/0382). The studies were conducted in accordance with the local legislation and institutional requirements. The participants provided their written informed consent to participate in this study.

## Author contributions

QH: Conceptualization, Data curation, Formal analysis, Methodology, Software, Visualization, Writing – original draft, Writing – review & editing. CW: Conceptualization, Data curation, Formal analysis, Methodology, Writing – review & editing. LC: Validation, Writing – review & editing. PZ: Validation, Writing – review & editing. WZ: Conceptualization, Funding acquisition, Methodology, Supervision, Writing – review & editing. FC: Conceptualization, Funding acquisition, Methodology, Supervision, Writing – review & editing.
