# Supplementary material for: Blood cell indices and inflammation-related markers with kidney cancer risk: a large-population prospective analysis in UK Biobank
Source: Front Oncol. 2024 May 23;14:1366449. doi: 10.3389/fonc.2024.1366449 (PMC11153768; doi:10.3389/fonc.2024.1366449)
Supplement: Supplementary file 2 [file DataSheet_1.pdf]

UK Biobank: Protocol for a large-scale prospective epidemiological resource

Protocol No: UKBB-PROT-09-06 (Main Phase)

UK Biobank Coordinating Centre
1 & 2 Spectrum Way
Adswood
Stockport
Cheshire SK3 0SA

Tel:0161-475-5360
Fax:0161-475-5361
E-mail: enquiries@ukbiobank.ac.uk

21 March 2007 (AMENDMENT ONE FINAL)

Contents

		Page
**1**	**Scientific rationale and design**	
1.1	Overall aims of UK Biobank prospective resource	3
1.2	Rationale for large size	6
1.3	Background to baseline questionnaire	17
1.4	Background to baseline physical measurements	23
1.5	Background to baseline samples	31
1.6	Planning and piloting	38
1.7	Assessment centre planning	49
**2**	**Development of the resource**	
2.1	Overall strategy	56
2.2	Identification and invitation	57
2.3	Baseline assessment	63
2.4	Sample processing	73
2.5	Potential for enhancements	78
2.6	Long-term follow-up	81
2.7	Data handling and security	89
2.8	Strategy for access	96
2.9	Organisation	100
**Annexes**		
1	UK Biobank committees and staff	105
2	References	107

1. SCIENTIFIC RATIONALE AND DESIGN

1.1 Overall aims of UK Biobank prospective resource

1.1.1 Reliable assessment of different causes of disease

Scientists have known for many years that our risks of developing different diseases are due to the complex interplay of different factors: our lifestyle and environment; our personal susceptibility (genes); and the play of chance (luck). But, despite this longstanding awareness, a clear picture of the combined effects of different factors on the risks of different diseases in different circumstances is yet to emerge. Cohorts to date have typically been characterised by small numbers of disease cases (which may yield unstable estimates due to random variations); incomplete or inadequate measures of potential risk factors (which may yield systematic under-estimates of disease associations); incomplete or inadequate measures of confounding factors (which may yield over- or under-estimates); and/or retrospective case-control designs in which the disease itself may influence risk factor levels (i.e. "reverse causality"). Consequently, to help assess the main causes of various chronic diseases quantitatively, there is now a strategic need to establish some large blood-based prospective epidemiological studies in a range of settings with prolonged and detailed follow-up of cause-specific morbidity and mortality.

The UK Biobank resource aims to include 500,000 people from all around the UK who are currently aged 40-69. This age group is being studied because it involves people at risk over the next few decades of developing a wide range of important diseases (including cancer, heart disease, stroke, diabetes, dementia). The UK National Health Service treats the single largest group of people anywhere in the world, and keeps detailed records on all of them from birth to death. Consequently, prolonged follow-up of participants through routine medical and other health-related records will allow the identification of comparatively large numbers of individuals who develop each of a wide range of disabling and life-threatening conditions. Because UK Biobank will involve extensive baseline questionnaire and physical measures, as well as stored blood and urine samples that allow many different types of assay (e.g. genetic, proteomic, metabonomic, biochemical and haematologic), it will be a uniquely rich resource for investigating why some people develop particular diseases while others do not. This will help researchers to understand the causes of diseases better, and to find new ways to prevent and treat many different conditions.

1.1.2 Value of prospective study designs

A variety of study designs can be used to investigate different aspects of the relationships between different exposures and the risk of disease. These include family-based studies of genetic factors, retrospective case-control studies of particular conditions, and prospective observational studies [1,2]. For the comprehensive and reliable quantification of the combined effects of

lifestyle, environment, genotype and other exposures on a variety of outcomes, a prospective study has a number of advantages [1]. As well as allowing a wide range of different conditions to be studied, exposures can be assessed prior to disease development, which avoids recall bias and allows investigation of factors that might be affected by disease processes and treatments (e.g. blood marker concentrations, blood pressure) or by an individual's response to developing some condition (e.g. weight, physical activity, diet). Prospective studies are also able to assess those conditions that cannot readily be investigated retrospectively (e.g. fatal conditions, dementia) and can include all cases of those diseases that have high case-fatality rates (e.g. myocardial infarction). Moreover, it is possible to make a broader consideration of both the risks and benefits associated with a specific exposure, through the inclusion of multiple endpoints (e.g. the full health effects of smoking on a wide range of disparate diseases; or the relevance of blood pressure to different types of vascular disease). In contrast with a retrospective design, a prospective study can also provide a more straightforward source of comparable controls selected from within the same population.

By comparison with family-based or retrospective case-control studies, much larger numbers of people need to be recruited into a prospective study and careful follow-up needs to continue for many years until sufficient numbers of cases of any particular disease have developed. Hence, for studying the impact on some particular condition of factors (such as genes) that are not likely to be materially influenced by development of that condition, alternative designs may well suffice. Family-based studies are particularly valuable for identifying genes that are causally related to disease (but may over-estimate their relevance to the general population), while retrospective case-control studies are efficient for rapid accrual of large numbers of cases of some particular disease (especially at younger ages when associations may be stronger) [2]. Even in such circumstances, however, an established large-scale prospective cohort provides a valuable resource for assessing the relevance of these and other factors in the general population. Moreover, as more factors are assessed and more health events accrue over time, the UK Biobank resource will become increasingly valuable (and cost-effective) to researchers for the assessment of the complex interplay between the effects of different factors (some of which may be influenced by the development of disease and so only reliably assessed in such a resource).

For all of these reasons, several large blood-based prospective cohorts have been established in recent years, and UK Biobank is intended to complement these existing resources. Studies conducted in different populations extend the range of exposures that can be considered: for example, the 500,000 person Kadoorie Study in China involves lower cholesterol levels than can be reliably studied in the UK or other developed populations [3]; and the 150,000 person Mexico City Prospective Study involves greater levels of obesity than in the UK [4]. Some of these studies have concentrated chiefly on assessment of certain types of exposure (e.g. diet in the 500,000 person European Prospective Investigation into Cancer and Nutrition [EPIC], which is being conducted in several European countries [5]) and/or of certain types of

outcome (e.g. cause-specific mortality and heart disease or cancer in the Kadoorie, Mexican and EPIC cohorts), and so will be particularly valuable for assessing the relevance of those particular exposures and outcomes. By contrast, UK Biobank aims to assess the relevance of a very wide range of exposures to a very wide range of health-related outcomes (i.e. not just mortality and cancer but also many other conditions that cause substantial disability). As is discussed later, the baseline questions and measurements have been chosen carefully to allow this wide assessment to be conducted in the whole cohort, and so too have the different blood and urine samples that are being collected and stored (see Sections 1.3-1.5). In addition, there is the potential for certain enhancements to be added in substantial subsets of the UK Biobank participants to allow more detailed assessment of certain exposures (see Section 2.5). Moreover, by imbedding UK Biobank within a single National Health Service which provides the overwhelming majority of health care, it is intended that a very wide range of conditions can be identified and validated with routine medical and other health-related records (see Section 2.6).

1.2 Rationale for large size

1.2.1 General approach to sample size calculations

UK Biobank will consist of at least 500,000 men and women from the UK general population aged 40 to 69. This age range allows investigation of the common causes of morbidity and premature mortality, and also allows ascertainment of events at an age where such cause-specific outcomes are generally well recorded, with less co-morbidity (and competing causes of mortality) than outcomes at older ages. The inclusion of at least 500,000 individuals is the result of consideration of the number of events required for the reliable quantification of a number of different factors on a range of diseases (see below), as well as practical concerns regarding design and cost. In particular, the inclusion of 500,000 participants still allows acquisition of sufficiently detailed exposure information while retaining feasibility within financial and organisational constraints.

This section focuses on the power of "nested case-control" studies based on the UK Biobank resource. Other types of analysis will also be undertaken using UK Biobank as a research platform (e.g. "case-cohort" comparisons), but analyses based on nested case-control studies will, in general, be the most limited in their statistical power. It is, therefore, the power of nested case-control analyses that may be viewed as being the primary *statistical* determinant of the size of UK Biobank. The sample size and statistical power of UK Biobank is considered from two perspectives. Firstly, the power profile of nested case-control studies is explored from a generic perspective: that is, given N cases and M unmatched controls, what is the minimum detectable odds ratio (MDOR) that can be detected with 80% power, under a variety of assumptions about the genetic and/or environmental exposure prevalence in the study population and about the particular analysis that is to be undertaken. Secondly, the likely number of cases that UK Biobank will generate of a range of pivotal complex diseases is investigated. Given the chosen design of UK Biobank, this indicates where each of these complex diseases may fit in the power profile.

1.2.2 Power profiles for nested case-control studies

The tables in this section detail the power profile for either a main effect (genetic or environmental), or a gene-environment interaction term, in an unmatched case-control study with binary exposure variables (genetic and/or environmental) analysed using unconditional logistic regression. This setting, which invokes both a binary outcome (case/control status) and a binary exposure (exposed: yes/no), will generally be the least powerful among corresponding settings that may be considered on a data set of equivalent size (e.g. all else being equal, the statistical power would typically be higher if the exposure variable was continuous). The power calculations were all based on simulation: a detailed description of the mathematical models used to generate these results may be found on the UK Biobank website [6]. These calculations make the following assumptions: (i) simulation and analysis are both based on a logistic regression model; (ii) interaction terms reflect

departures from additivity on the log-odds scale (i.e. departures from a multiplicative model); and (iii) each nested case-control study contains four unmatched controls for each case.

1.2.2.1 A conventional power profile

Table 1.2.1 details the "conventional" power profile for the binary main effect (genetic or environmental). The tabulated MDORs are indexed by: (i) the number of cases available for study in a nested case-control study (2500, 5000, 10,000 or 20,000); (ii) the prevalence of the "at risk" exposure category of the binary genetic and environmental risk factors (0.5, 0.25, 0.1, 0.05 or 0.01); and (iii) the two-tailed p-value used to define statistical significance in particular circumstances (0.01, 10^{-4} or 10^{-7}). Here, the term "conventional power profile" implies that no account is taken of power loss consequent upon certain issues, such as misclassification errors in assessment of the exposure of outcome, or subject-to-subject variation in the baseline risk of developing the outcome of interest (which are considered in Section 1.2.3).

Exposure prevalence	Critical P-value	Minimum detectable OR for main effect (4 controls per case)			
		2,500 cases	5,000 cases	10,000 cases	20,000 cases
0.5	0.01	1.16	1.11	1.08	1.06
0.5	10^{-4}	1.23	1.16	1.11	1.08
0.5	10^{-7}	1.32	1.22	1.15	1.10
0.25	0.01	1.19	1.13	1.09	1.06
0.25	10^{-4}	1.28	1.19	1.13	1.09
0.25	10^{-7}	1.37	1.25	1.17	1.12
0.1	0.01	**1.28**	**1.19**	**1.13**	**1.09**
0.1	10^{-4}	1.39	**1.26**	**1.18**	**1.12**
0.1	10^{-7}	1.54	1.36	**1.24**	**1.16**
0.05	0.01	1.39	1.26	1.18	1.12
0.05	10^{-4}	1.59	1.39	1.26	1.80
0.05	10^{-7}	1.80	1.51	1.34	1.23
0.01	0.01	1.99	1.63	1.41	1.28
0.01	10^{-4}	2.50	1.91	1.58	1.38
0.01	10^{-7}	3.16	2.26	1.78	1.51

Table 1.2.1: MDORs associated with 80% statistical power for main effects (genetic or environmental) by exposure prevalence and critical significance test level in a conventional analysis of power

In genetics, the genotype at a given locus typically has 3 levels (i.e. with alleles G and g, there are three genotypes GG, Gg and gg) and, all else being equal, inferences based on a single parameter summarising the effect of the 3 level genotype will typically be more powerful than inferences based on the equivalent binary exposure variable. A genetic determinant will act as if it is binary if expression of the G allele is either "dominant" (GG & Gg versus gg) or "recessive" (GG versus Gg & gg). In the case of an analysis involving a genotypic exposure variable, the least powerful setting considered here may, therefore, be viewed as reflecting one of these two settings. Genetic and environmental exposures are treated as being equivalent in Table 1.2.1.

Using arguments based on the prior probability that a true association will exist between a given genetic determinant and the disease of interest [7], it may reasonably be argued that, in a genetic association study, $p<10^{-4}$ can be used as a reasonable definition of statistical significance under circumstances where the genetic exposure is defined on the basis of a variant lying in a vaguely defined candidate gene; here, "candidature" may be based on biological plausibility or linkage-based genomic positioning. For the purpose of a whole genome association-based scan, however, $p<10^{-7}$ is a more appropriate definition of statistical significance [8,9].

Table 1.2.2 details the conventional power profile for the gene-environment interaction term in a model otherwise equivalent to that in Table 1.2.1. The interaction OR reflects the magnitude of departure from the OR based solely on a simple multiplicative model using the main effects. So, for example, if the OR associated with the binary genetic determinant in subjects that are *unexposed* to the "at risk" level of the environmental exposure is 1.6, while the equivalent OR in those that are *exposed* to that environmental determinant is 2.0, the interaction OR would be 2.0÷1.6=1.25.

**Genotype prevalence**	**Environmental prevalence**	**Critical P-value**	**Minimum detectable OR for interaction effect (4 controls per case)**			
			**2,500 cases**	**5,000 cases**	**10,000 cases**	**20,000 cases**
0.5	0.5	0.01	1.37	1.25	1.17	1.12
0.5	0.5	10^{-4}	1.54	1.36	1.24	1.16
0.5	0.5	10^{-7}	1.80	1.51	1.34	1.23
0.25	0.25	0.01	1.46	1.31	1.21	1.14
0.25	0.25	10^{-4}	1.69	1.45	1.30	1.20
0.25	0.25	10^{-7}	1.96	1.61	1.40	1.27
0.1	0.1	0.01	2.07	**1.67**	**1.44**	**1.29**
0.1	0.1	10^{-4}	2.62	**1.98**	**1.62**	**1.41**
0.1	0.1	10^{-7}	3.28	2.31	**1.81**	**1.52**
0.05	0.05	0.01	3.42	2.39	1.85	1.54
0.05	0.05	10^{-4}	5.02	3.13	2.24	1.77
0.05	0.05	10^{-7}	7.24	4.05	2.69	2.01
0.05	0.5	0.01	1.88	1.56	1.37	1.25
0.05	0.5	10^{-4}	2.34	1.82	1.53	1.35
0.05	0.5	10^{-7}	2.89	2.12	1.70	1.46
0.5	0.05	0.01	1.88	1.56	1.37	1.25
0.5	0.05	10^{-4}	2.34	1.82	1.53	1.35
0.5	0.05	10^{-7}	2.89	2.12	1.70	1.46

Table 1.2.2: MDORs associated with 80% statistical power for gene-environment interactions effects by exposure prevalence and critical significance test

1.2.2.2 Commentary on conventional power profiles

In light of plausible estimates of the size of the relative risks for many genetic variants associated with complex disease [10], it may be argued that it would be desirable for a nested case-control study based on the UK Biobank resource to be able to detect an OR associated with a main effect of 1.33 or more with a statistical power of at least 80% when the exposure has a prevalence of 10% or more. Similarly, it may be viewed as desirable to be able to detect an interactive odds ratio of 2.0 or more with similar power when either of the two binary exposures has such a prevalence. The underlined cells in bold in Tables 1.2.1 and 1.2.2 indicate circumstances where these requirements are met. Based on approximate linear interpolation of Table 1.2.1, the conventional power profile suggests that it would be desirable to have approximately 3,500 cases (with 4 unmatched controls per case) for an analysis based on a main effect OR≥1.33 reflecting a variant in a vague candidate gene ($p<10^{-4}$) and 6,000 for an analysis forming part of a whole genome association scan ($p<10^{-7}$). Similarly, when interest focuses on interactions, the conventional power analysis in Table 1.2.2 suggests that the numbers of cases required to meet these requirements for OR≥2.0 are approximately 5,000 and 10,000 respectively.

1.2.2.3 Taking account of realistic bioclinical complexity

In this sub-section, the previous power calculations are repeated with account taken of the impact of realistic bioclinical complexity, as represented by additional elements that are added into the simulation model. It is here that the additional flexibility permitted by the simulation-based approach becomes invaluable. The following additional assumptions are made: (i) there is unobservable subject-to-subject heterogeneity in the baseline risk of developing disease, which is of such a magnitude that a subject on the highest 97.5% population centile for risk is at 100 times the risk of a subject on the lowest 2.5% population centile; (ii) there is a symmetrical 1% genotyping error (i.e. in a random 1% of subjects, the correct genotype is replaced by a genotype that implies the wrong “at risk” status); (iii) there is a symmetrical 20% misclassification error in assessing the environmental exposure (i.e. in a random 20% of subjects, the true environmental exposure is replaced by the incorrect exposure); (iv) the identification of cases is of low sensitivity (i.e. only 20% of all cases arising in the population are identified by the available follow-up systems); (v) the probability that a non-diseased participant is incorrectly classified as a disease case is 0.2%; and (vi) as there are many more non-cases than cases, the combination of the last two assumptions means that approximately 33% of designated cases do not have the disease while 1.5% of designated controls do have the disease.

Because there are so many scenarios that might be considered, this one set of assumptions should not be seen as representing a “true,” or even “optimal”, set of assumptions with which to work. Furthermore, even if the “true” assumptions were known, they would inevitably vary from disease to disease and from exposure to exposure. Rather, these conservative assumptions have been chosen to reflect what might typically occur when relying entirely

on the environmental exposure assessment at the baseline visit and on the outcome classification defined via routine health information systems, in order to assess the impact on the conventional power profiles detailed in Section 1.2.2.1. Subsequently, the impact of modifying some of these assumptions is also considered.

1.2.2.4 Impact of bioclinical complexity on power profiles

Table 1.2.3 suggests that, under the particular set of assumptions about bioclinical complexity detailed above, detection of a genetic main effect associated with a binary genotype with prevalence of 10% and odds ratio of 1.33 that required 3,500 cases (with 4 unmatched controls per case) under the conventional power profile for $p<10^{-4}$ needs to be increased to between 8,000 and 10,000 cases. Similarly, the required number of cases for a genome-wide association analysis at $p<10^{-7}$ is increased from 6,000 to 10-12,000 cases. For many realistic research questions that may be posed in relation solely to environmental exposures at p<0.01, the sample size requirement will also be in the range 5,000 to 10,000 cases. Finally, for the detection of gene-environment interactive odds ratio <2.0 under settings where either the at-risk genotype or environmental determinant has a prevalence as low as 10%, it will generally be desirable to have closer to 20,000 cases (Tables 1.2.4a-c).

**Exposure prevalence**	**Critical P-value**	**Minimum detectable OR for main effect (4 controls per case)**			
		**2,500 cases**	**5,000 cases**	**10,000 cases**	**20,000 cases**
0.5	10^{-4}	1.39	1.27	1.19	1.13
0.5	10^{-7}	1.52	1.35	1.24	1.16
0.5	0.01	1.47	1.32	1.22	1.16
0.33	10^{-4}	1.39	1.28	1.19	1.14
0.33	10^{-7}	1.54	1.39	1.24	1.18
0.33	0.01	1.51	1.35	1.26	1.17
0.2	10^{-4}	1.47	1.32	1.24	1.16
0.2	10^{-7}	1.63	1.44	1.30	1.21
0.2	0.01	1.69	1.47	1.32	1.23
0.1	10^{-4}	1.65	1.46	**1.31**	**1.22**
0.1	10^{-7}	1.87	1.60	1.42	**1.27**
0.1	0.01	2.14	1.74	1.52	1.38
0.05	10^{-4}	1.99	1.67	1.48	1.32
0.05	10^{-7}	2.30	1.86	1.65	1.41
0.05	0.01	3.15	2.44	1.99	1.68

Table 1.2.3: MDORs associated with 80% power for main effects (genetic or environmental) by exposure prevalence and critical significance test level (with allowance for assumed bioclinical complexity)

a) 5,000 cases and 20,000 controls		Genotype prevalence			
		**0.1**	**0.2**	**0.33**	**0.5**
**Environmental prevalence**	**0.1**	3.94	2.88	2.80	2.48
	**0.2**	2.95	2.46	2.14	2.10
	**0.33**	2.65	2.25	2.01	2.03
	**0.5**	2.98	2.29	2.10	2.12
**(b) 10,000 cases and 40,000 controls**					
**Environmental prevalence**	**0.1**	3.03	2.36	2.11	2.05
	**0.2**	2.32	1.95	1.87	1.78
	**0.33**	2.15	1.80	1.68	1.64
	**0.5**	2.16	1.86	1.70	1.70
**(c) 20,000 cases and 80,000 controls**					
**Environmental prevalence**	**0.1**	2.47	**1.94**	**1.82**	**1.72**
	**0.2**	**1.97**	1.67	1.58	1.54
	**0.33**	**1.79**	1.58	1.47	1.45
	**0.5**	**1.79**	1.61	1.46	1.44

Table 1.2.4: MDORs associated with 80% power for gene-environment interaction by joint exposure prevalences at significance test level $p<10^{-4}$ (with allowance for assumed bioclinical complexity)

1.2.2.5 Changing assumptions about bioclinical complexity

Formal testing indicated that the type 1 error associated with the model-based analysis of the simulated data sets was nominal both for main effects and for interactions [6]. Furthermore, the simulated size of main effects had little impact on the estimated MDORs for the interactions. All of the analyses considered above assumed that there were four times as many unmatched controls as there were cases. For a fixed number of cases, there are tangible benefits in statistical power associated with increasing the control:case ratio from 1:1 to 4:1. Indeed, when a particularly rare determinant (such as an interaction) is being studied, it may be beneficial to increase the control:case ratio beyond 4:1 [6]. Consequently, given that multiple nested case-control studies will be conducted within UK Biobank, it may be cost-effective to establish a large common control group that is subject to comprehensive genotyping (i.e. allowing case-cohort approaches).

It was assumed in all of the analyses in Sections 1.2.2.3-4 that there was a 100-fold variation in the underlying risk of disease between a subject on the general population 97.5% centile and one on the 2.5% centile. But, the estimated MDORs were found to be remarkably insensitive to the choice of this frailty variance [6]. It was also assumed that the disease prevalence in a subject who was at the at-risk level for neither the genetic nor the environmental determinant was 1%. As demonstrated by others [11], however, the estimated MDORs are reasonably robust to changes in the baseline prevalence of disease; in particular, the MDORs changed little if the baseline prevalence was changed from 1% to 0.1%.

The exposure and outcome misclassification rates used in the analyses reported above are meant to reflect a situation in which exposure data are obtained at recruitment, and the binary outcomes are taken precisely as

recorded in the routine health information systems. But, if additional time and resources are invested in repeating assessments of exposure (see Section 2.5), and in refining outcome data (see Section 2.6), these misclassification rates will fall. For example, reducing the misclassification rate for the environmental exposure from 20% to 10%, and the proportion of non-diseased subjects incorrectly inferred to be cases from 0.2% to 0.045%, reduces the MDORs (for $p<10^{-4}$) for the genetic and environmental main effects and for the interaction term from 1.32 to 1.24, 1.66 to 1.33, and 2.35 to 1.81, respectively, in a study with 5,000 cases and 20,000 controls and with genetic and environmental exposure prevalences of 20%. This corresponds to only 10% of cases really being disease-free, as opposed to 33% under the original assumption. But, although these sensitivity analyses indicate that gains can be obtained in statistical power by refining the assessment of exposures and outcomes, these gains come at the cost of investing more time and resources in re-assessing subjects. There is no doubt that re-assessment of this nature will be valuable for some scientific questions and less important for others.

In analyses of nested case-control studies based on the UK Biobank resource, ethnic substructure will need to be considered. Even when "self-reported" ethnic group is taken into account, confounding by ethnicity can still impact on studies of the genetic determinants of complex disease. There is active ongoing debate as to how important this will be in practice [12-14], and how problematic it will be in the UK population specifically. All that can be said at present is that adjustments for ethnic stratification (such as "genomic control") can reduce the effective sample size, and their impact on statistical power will be relatively greater in studies that are looking for smaller relative risks. The Wellcome Trust is currently funding two projects that are investigating population substructure in the UK general population. So, by the time analysis of the UK Biobank resource starts, there should be a much clearer picture of the pattern of latent ethnic stratification in the British population and of how best to deal with it. No quantitative adjustment has been made to the present power calculations to address this issue as it is entirely unclear how large that adjustment (if any) should be (Lon Cardon: personal communication).

1.2.2.6 Summary on power profiles

The analyses above indicate that 5-10,000 cases would typically be needed for reliable nested case-control studies of environmental or genetic main effects across a wide range of biomedical research questions for which UK Biobank might realistically be used as a scientific platform, and across a range of realistic assumptions about bioclinical complexity. In such circumstances, when the exposure prevalence is 10%, 5,000 cases will enable the reliable detection of ORs of the order of 1.5, while 10,000 cases will enable the detection of ORs of around 1.33. When the primary interest focuses on interactive effects, there will often be a need for closer to 20,000 cases, even to detect interactive ORs of as much as 2.0. In order that such large numbers of cases may be generated for any given complex disease of interest, it is clear that UK Biobank must be very large. Although the

calculations that generated these conclusions invoked a range of assumptions about the underlying bioclinical setting, these fundamental conclusions are reasonably robust to the particular assumptions that were made. The next section explores the rate at which binary disease end points may be expected to arise within UK Biobank, given an initial sample size of 500,000 recruits.

1.2.3 Expected numbers of cases of various conditions

1.2.3.1 Incident cases developing during follow-up

The predicted occurrence of events in UK Biobank was generated by simulation for selected conditions of interest. (This list is not intended to be exhaustive, but instead is intended to illustrate the likely power of UK Biobank for important clinical conditions with a range of incidence rates.) Full details of this analysis and the information sources that were used to obtain death, disease and migration rates are available on the UK Biobank web site [6]. It was assumed that 500,000 participants between the ages of 40 and 69 years will be recruited over 3-5 years with an age-sex distribution at recruitment corresponding to the age-sex distribution in the relevant age ranges across Great Britain as a whole at the 2001 Census. The simulated participants were then followed dynamically over time with the application of appropriate age- and sex-specific death and incidence rates. All of the simulations take appropriate account of two classes of loss-to-follow-up: (i) migration overseas; and (ii) withdrawal from UK Biobank with a demand that there be no further follow-up through routine health information systems. For convenience, this second class of loss-to-follow-up is referred to as “comprehensive withdrawal” and is assumed to amount to no more than 1/500 subjects per annum (which seems likely to be a rather large overestimate). By simultaneously considering death, disease incidence, overseas migration and comprehensive withdrawal, the analyses take appropriate account of the gradual attrition of the cohort as a whole. Subjects are considered to be no longer “at risk” of developing a specific condition once they had developed that condition, but they remain “at risk” of developing all other conditions.

Table 1.2.5 summarises the number of health events that might be anticipated in UK Biobank after taking account of such losses-to-follow-up as well as the fact that recruits to cohort studies are typically more healthy than the general population (i.e. “healthy cohort effect”). These detail the expected time after the commencement of recruitment that will be required for UK Biobank to generate 1,000, 2,500, 5,000, 10,000 and 20,000 cases of sixteen important complex diseases. Table 1.2.5 is adjusted for the impact of migration overseas and for comprehensive withdrawal, and so pertains to settings in which there is no need to contact subjects at the time of undertaking the nested case-control study in order to refine the exposure assessment or disease outcome.

Condition	Time to achieve				
	**1,000 cases**	**2,500 cases**	**5,000 cases**	**10,000 cases**	**20,000 cases**
**Bladder cancer**	11 years	19 years	31 years	-	-
**Breast cancer (F)**	4 years	6 years	10 years	17 years	40 years
**Colorectal cancer**	5 years	9 years	14 years	22 years	42 years
**Prostate cancer (M)**	6 years	9 years	14 years	22 years	41 years
**Lung cancer**	7 years	12 years	19 years	34 years	-
**Non-Hodgkins lymphoma**	11 years	22 years	-	-	-
**Ovarian cancer (F)**	12 years	26 years	-	-	-
**Stomach cancer**	16 years	29 years	-	-	-
**Stroke**	5 years	8 years	12 years	18 years	28 years
**MI and coronary death**	2 years	4 years	5 years	8 years	13 years
**Diabetes mellitus**	2 years	3 years	4 years	6 years	10 years
**COPD**	4 years	6 years	8 years	13 years	23 years
**Hip fracture**	7 years	11 years	15 years	21 years	31 years
**Rheumatoid arthritis**	7 years	14 years	27 years	-	-
**Alzheimer's disease**	7 years	10 years	13 years	18 years	23 years
**Parkinson's disease**	6 years	10 years	15 years	23 years	37 years

Table 1.2.5: Expected years after starting recruitment before 1,000, 2,500, 5,000, 10,000 and 20,000 cases of 16 diseases of interest occur (with allowance for healthy cohort effect, overseas migration and comprehensive withdrawal of 1 in 500 participants)

In some circumstances, re-assessment of the exposure assessment or disease outcome may be considered valuable. Analyses were performed with further adjustment for a proposed loss-to-follow-up model that reflects the experience of the 1958 Birth Cohort [15]. This model entails approximately 5% of subjects withdrawing almost immediately (within the first year) and a subsequent on-going withdrawal rate of 1.4% per annum. Similar estimates were provided by the proportion of participants in the Whitehall study of Civil Servants who were willing to be re-assessed after about 20 years [16]. As these estimates already take account of migration overseas and the equivalent of comprehensive withdrawal from that study, these two elements are not included as additional causes of loss-to-follow-up. In general, this model added about 1 year to the time taken to reach a particular number of events by the end of the first decade of follow-up (i.e. increasing 9 years to 10 years) and about 2 years by the end of the second decade of follow-up [6].

By about the end of the first decade (i.e. around 2015) in either scenario, there will be about 20,000 cases of diabetes mellitus, more than 10,000 cases of MI and coronary death, more than 5,000 cases of COPD, and 5,000 cases of breast cancer. By the fifteenth year of follow-up (ie. around 2020), there will also be at least 5,000 cases of stroke, Alzheimer's disease, Parkinson's disease, colorectal cancer and prostate cancer. In other words, UK Biobank will have generated at least 5,000 incident cases for 8 of these 16 conditions by about 2020, and so should be sufficiently mature to allow reliable assessment of the determinants of these conditions. Moreover, it will also have generated similar numbers of cases of a range of other important conditions, and these numbers will continue to increase as follow-up though health-care records continues.

1.2.3.2 Prevalent cases identified at baseline

Table 1.2.6 details the expected number of prevalent cases of selected chronic diseases that will be identified at the baseline assessment of the UK Biobank resource. These estimates have been obtained from population prevalence data in Morbidity Statistics from General Practice Fourth National Study (MSGP4) 1991-92 [17], supplemented by the General Practice Research Database (GPRD) 1998 for COPD and Health Survey for England (HSE) 2003 for diabetes mellitus [6]. The right-hand column in Table 1.2.6 details the expected numbers of cases down-weighted by 50% to take account of the intrinsically “healthy” nature of the UK Biobank subjects that is likely. Using the same indicative sample size requirements derived for case-control studies based on incident cases, it is clear that there should be adequate numbers of prevalent cases at recruitment to study a wide range of important complex diseases. In particular, for several of these diseases there will be between 5,000 and 10,000 cases at baseline which would allow detection of ORs of between 1.33 and 1.5 associated with exposures with a prevalence as low as 10%. Case-control studies based on prevalent cases could provide opportunities for important early results from the UK Biobank resource, although it should be noted that such retrospective studies do not enjoy the key advantages of a prospective study (as outlined in Section 1.1.2).

Condition	Data source	Sex	Age band:		Total each M & F	Total both M & F	50% down-weight
			45-64 years	65-74 years			
**Diabetes (type 1 and 2)**	HSE 2003	M	6,902	5,918	12,820		
	HSE 2003	F	4,365	4,268	8,633	21,453	10,726
**Diabetes mellitus**	MSGP4	M	8,919	1,779	10,698		
	MSGP4	F	3,285	1,377	4,662	15,360	7,680
**Ischaemic Heart Disease**	MSGP4	M	8,273	3,446	11,719		
	MSGP4	F	3,754	2,035	5,789	17,508	8,754
**Angina pectoris**	MSGP4	M	5,355	2,172	7,527		
	MSGP4	F	2,837	1,487	4,324	11,851	5,925
**COPD**	GPRD 1998	M	4,589	*3,510	8,099		
	GPRD 1998	F	4,106	2,312	6,418	14,517	7,258
**COPD**	MSGP4	M	937	1,971	2,908		
	MSGP4	F	2,923	1,140	4,063	6,971	3,485
**Stroke**	MSGP4	M	5,668	2,045	7,713		
	MSGP4	F	3,776	1,704	5,480	13,193	6,596
**Parkinson’s disease**	MSGP4	M	1,334	558	1,892		
	MSGP4	F	1,088	372	1,460	3,352	1,676
**Rheumatoid arthritis**	MSGP4	M	917	258	1,175		
	MSGP4	F	1,813	543	2,356	3,531	1,765

* Rates are for 65y+ (not 65-74y), and differences from MSGP4 may relate to definitions used

Table 1.2.6: Expected numbers of participants with selected chronic diseases at baseline assessment for the UK Biobank resource using various population prevalences and target recruitment numbers

1.2.4 Conclusions

With the recruitment of 500,000 middle-aged adults, UK Biobank will provide a powerful platform for studying a range of complex diseases that are of great relevance to public health. In the early phases of the resource (i.e. the first 10-15 years), extensive and powerful research will be able to be undertaken on incident cases of some of the more common conditions (including diabetes mellitus, coronary heart disease, COPD and breast cancer) as well as on some aspects related to conditions already present at recruitment. Beyond the fifteen year (i.e. after 2020), at least 10 complex diseases will generate 10,000 and then 20,000 incident cases, and many other conditions will generate enough cases to ensure that UK Biobank provides a valuable platform for population-based research. By maintaining close and active contact with other similar resources, UK Biobank can also ensure that it is in a position to make a major contribution to collaborative initiatives to support the investigation of rarer conditions, and the earlier study of both main effects and interactions. But, if UK Biobank were to involve substantially less than 500,000 people, it would clearly be considerably less valuable as a stand-alone project and would only be able to contribute as one part of a network of large cohorts.

1.3 Background to baseline questionnaire

1.3.1 General approach to prioritisation

Collection of lifestyle and other potentially health-related information through self-completed questionnaires and interview complements the physical measurements and biological samples collected at the baseline assessment visit for UK Biobank, and will form a database that allows a wide range of research questions – both anticipated and unforeseen – to be addressed in the future. Due to the broad scope of this resource (as well as time and cost constraints), the emphasis in the baseline questionnaire has been to concentrate on known and potential risk factors for outcomes that are already, or are projected to become, important public health concerns for the adult population. Certain criteria were established to assist in prioritising questions related to potential exposures and confounders. These criteria included: the perceived strength of knowledge or hypotheses about exposure-disease relationships; the public health importance of the relevant condition; the likely importance of factors that might act as confounders or sources of bias; the reliability and validity of questionnaire measures; and the availability of alternate sources of information about the factor (including biometric parameters and biological samples assessed at baseline, and past medical and other health-related records). Further, it was considered important that the measured exposures typically have a reasonable prevalence (e.g. at least 15%) in the population so that there would be sufficient power to examine their relevance reliably, both overall and in different circumstances (i.e. at different levels of other exposures) [18].

With respect to feasibility, the comprehension and acceptability of each question, the time taken to complete each of them, and their response distributions were examined in pilot studies, which aided the final selection and presentation of suitable questions. The UK Biobank questionnaire is administered in two sequential parts during the assessment centre visit: a touch-screen self-completed questionnaire followed by a computer-assisted personal interview (CAPI). Due to the relative staff costs for self-completed versus interviewer-administered questions, topic areas and questions considered of an exploratory nature have been restricted to the self-completed questionnaire (wherever possible), and questions that needed to be asked by an interviewer required greater evidence of their value to be included. Because significant variations in lifestyle and other factors (e.g. diet) typically occur over time, repeat assessments will be required in substantial subsets of the UK Biobank cohort throughout follow-up in order to quantify, and make allowance for, this variation (see Section 2.5.1).

1.3.2 Questionnaire structure and administration

Due to the large size of the UK Biobank cohort, the approach to data capture aimed to optimise the accuracy and completeness of the data collected, while also maximizing the efficiency of the process. Computerized direct data entry methods were selected in preference to conventional paper questionnaires as these allow internal consistency checks, automated coding, immediate

access, and ongoing monitoring and audit. The computer technology devised to record questionnaire responses has been developed specifically for UK Biobank based on an existing platform used previously in large-scale studies. It has been piloted to determine its usability and acceptability among potential participants, and has been enhanced in the light of that experience.

Following completion of the consent procedures (which also use the direct data entry system), the touch-screen self-administered questionnaire is used to collect the majority of information. This questionnaire typically takes participants about 30 minutes to complete with a single member of staff able to monitor and assist (as required) about 10-12 participants simultaneously, which makes it particularly efficient. Moreover, the touch-screen questionnaire is designed so that participants are only asked questions that are directly relevant to themselves (e.g. reproductive history and oral contraceptive use are only asked of women; detailed smoking habits only asked of those who have smoked). Because it involves direct computer entry by participants rather than interview, privacy is enhanced and there have been high response rates to sensitive questions during piloting (although such questions can be skipped if preferred).

Information that is not readily collected via the touch-screen system (e.g. not involving categorical or numerical responses; requires detailed questioning) is collected in a subsequent computer-assisted personal interview (CAPI), which is designed to last only about 5-10 minutes to control staff costs. A pre-visit aide memoire is provided to participants prior to attending the assessment centre so that they can note certain information (e.g. medications, operations, family history, and birth details) that may be difficult or time-consuming for them to recall during the visit. Certain questions are only asked in the interview if the participant has given particular answers to certain “screening” questions on the touch-screen. For example, if a participant indicates on the touch-screen that they have particular medical conditions, then the interviewer will be prompted to ask the participant specific questions about these conditions. Pre-coded lists of diseases, drugs, and occupations are built into the CAPI system, along with structured search facilities, to help this information to be recorded (and automatically coded) both rapidly and completely. Other innovations to improve data quality and efficiency of collection include the use of inbuilt cross-checks between relevant questionnaire responses, and check messages when extreme values are entered or when no value is provided.

1.3.3 Overview of questionnaire scope

The UK Biobank questionnaire can be categorised into the following broad topic areas of interest: sociodemographics and occupation; lifestyle exposures (including smoking, alcohol, physical activity and diet); early life exposures; psychological state; cognitive function; family history of illness; and medical history and general health. A review of questionnaires previously used in observational studies, clinical trials and population surveys was conducted in order to identify appropriate questions to quantify exposures in these areas, and there was wide consultation with international experts in each area of

interest. In some cases, validated questionnaires for the topics of interest were too extensive to be included in their entirety, or the questions were inappropriate for a general population cohort. In adapting questionnaires where short scales were not available, attention was given to those questions likely to be reliably reported, simple to answer and with a wide range of responses (and this was assessed in the pilot studies). For most of the topic areas, the questions to select for inclusion in the UK Biobank questionnaire were unambiguous and non-contentious. Questions about sociodemographic factors, smoking, alcohol, family history, early life exposures, general health and disability have been utilized in many population studies, and there was little difficulty in selecting validated and important sets of questions that could be readily answered by participants. For certain topic areas (e.g. cognitive function), however, decisions about development of the questionnaire were less straightforward.

1.3.3.1 Sociodemographic factors

Socioeconomic position and demographic markers are known to be correlated with mortality, measures of morbidity and access to health services [19-21]. Hence, assessment of these factors, both as potential exposures and as confounders, is necessary for any longitudinal study. A variety of variables were considered important to assess a range of potential factors that both inform on material deprivation, social deprivation, socioeconomic class and education, and also correlate well with measures of health status (including mortality, morbidity and hospital admissions) [22, 23]. Questions have been included on housing tenure, car ownership, household income, household structure, employment status and current occupation, ethnicity and country of birth, qualifications and school leaving age. These questions were mostly sourced and adapted from general population surveys (such as the 2001 Census and the Health Survey for England) where they had been tested extensively on large and diverse populations.

1.3.3.2 Smoking and alcohol

In developed countries, tobacco smoking and alcohol consumption are the leading lifestyle exposures contributing to disease burden [24, 25]. Tobacco is a known risk factor for lung and other cancers, cardiovascular diseases, chronic obstructive pulmonary disease and a number of other respiratory conditions. Alcohol consumption has been associated with ischaemic heart disease, stroke, certain cancers, cirrhosis of the liver, various psychiatric disorders and injury [26]. Smoking behaviour questions were adapted from various longitudinal epidemiological studies and surveys, as well as after consultation with experts in the field. Due to the magnitude of the risk association of tobacco smoking with both common cancers and cardiovascular diseases, and the knowledge regarding dose-response, duration and temporal relationships to mortality [27], the questions on smoking are very comprehensive. But, since detailed questions are only asked of those who have smoked, they impose little time overall (an average of 30 seconds on the touch-screen in piloting). Alcohol consumption is assessed with quantity-frequency type questions, and include beverage

specificity because of evidence to suggest this may improve under-reporting [28], as well as being a factor of interest in its own right. For both smoking and alcohol exposure, reasons for recent stopping are investigated to allow the possibility of reverse causality to be taken into account.

1.3.3.3 Family history and early life exposures

Associations of *in utero* and early childhood exposures with common diseases of adult life have been widely reported. Questions on birth weight, breastfeeding, maternal smoking, childhood body size and residence at birth were selected as these have been identified as potential predictors of adult health [29, 30]. Family history is a known predictor of common cancers, cardiovascular diseases and a number of other medical conditions. Consequently, questions are included relating to a limited family history among first degree relatives of common serious illnesses, as well as about being a twin or other multiple order birth. These questions could identify potential subgroups of interest for more intensive family-based studies in the future. In order to control for potential biases in future statistical analyses, parental details (non-identifying) are requested with the purpose of linking siblings within the cohort. Given that all these questions rely upon participant recall, inclusion of these factors was balanced against their likely validity [31, 32].

1.3.3.4 General health and disability

Medical history, reproductive history for women, general health questions, self-reported disability, as well as some limited phenotype information (related to skin and hair colour, chronic pain and chest pain, wheeze), will be collected using standardized questions adapted from various health surveys and longitudinal studies conducted in Britain. These factors are important in any analysis examining health outcomes, both to take account of known and potential predictors of future disease and to identify prevalent health states. Baseline medical history can also be used to select populations of interest within the cohort to follow with respect to molecular and genetic predictors of disease progression and prognosis. To ensure that the self-reported medical history and medication use is well discriminated, automated coding databases have been developed within the CAPI system, which will be administered by trained interviewers. In order to validate and reinforce this self-reported information, it will be linked with the participants' past medical records (see Section 2.6).

1.3.3.5 Environmental factors

A large number of potential environmental exposures were considered for inclusion in the UK Biobank questionnaire. Questions were selected that were feasible to collect within the limited available time, considered to be predictors of common diseases (such as respiratory illness and musculoskeletal conditions), and provided valid and reasonable response distributions. These include current address, residence at birth, occupation and other workplace factors, passive smoke exposure, indoor air pollution and mobile phone use

[33, 34]. Current address will allow researchers to explore multiple potential environmental risk factors by linkage with UK ecological databases (whilst maintaining participant confidentiality). Occupation is collected by trained interviewers with the Standard Occupational Classification 2000 [35] built into the CAPI system. This allows precise and discriminatory occupational categorization, and the ability to explore the relevance of this factor as a socioeconomic and environmental determinant of disease. In addition, the collection of blood and urine samples will allow concurrent quantification of specific environmental exposures (such as cotinine for cigarette smoke, or heavy metals such as lead, cadmium and mercury) which can be used to complement questionnaire assessment of these exposures.

1.3.3.6 Dietary habits

Observational studies and randomised trials have provided conflicting evidence regarding the effects of various dietary components (such as fat and fibre) on important disease outcomes [36-38] and about the most appropriate method to approach measurement [39-41]. The availability of biological samples in the UK Biobank resource will allow the direct measurement of the levels of many biomarkers of interest (e.g. lipid profile, vitamins, red cell fatty acids). But, since biomarkers do not necessarily reflect true intakes [42] and are not available for many dietary items, questionnaire methods must also be employed. All currently validated questionnaires on diet – namely the food frequency questionnaire, 24 hour dietary recall and multiple day food diaries – can involve significant time and resources for both their completion and subsequent coding. Indeed, the resources required to code multiple day food diaries can be so substantial that they are typically archived in large studies and only coded on a nested case-control basis.

Within the context of UK Biobank, it has been necessary to strike a balance between the resources used to assess diet and those used for other factors known to be important causes of a wide range of conditions. A relatively short set of self-completed food frequency questions has been selected to rank participants at baseline according to commonly eaten food groups based on the expected distribution in the British population, as well as seeking information about some common sources of various nutrients [43]. It is recognised that this approach does not allow assessment of total energy intake or some specific nutrients. Hence, it is planned to supplement this information by administering repeated 24-hour dietary recall questionnaires remotely via the internet (with the pilot experience indicating that more than half of all participants will have internet access and be willing to be re-contacted via e-mail). A self-administered questionnaire suitable for internet use and coding (based on the EPIC-soft 24-hour recall questionnaire) is now being developed in conjunction with scientists at the National Institutes of Health and the International Agency for Research on Cancer for this purpose.

1.3.3.7 Physical activity

The questions on physical activity that have been included in the UK Biobank questionnaire were adapted, based upon piloting, from a validated survey

instrument [44]. They are principally intended to allow participants to be ranked according to their levels of physical activity (vigorous, moderate and walking). In addition, questions on common sedentary activities have been included to provide a composite measure of physical inactivity [45, 46]. It is also intended to collect additional questions, based on a 24-hour recall of daily activities, via the internet. As for diet and various other relevant lifestyle factors, repeat assessments of activity will be required in representative subsets of the UK Biobank cohort throughout follow-up to take account of variations that occur over time (see Section 2.5.1). Repeat assessment visits for these subsets of participants not only allow the standard baseline questions about activity to be repeated in order to make allowance for variation over time, but also provides an opportunity to conduct more intensive assessments of physical activity (e.g. heart rate monitoring to estimate energy expenditure) that can be used to characterise baseline activity in the whole cohort more completely (see Section 2.5.2).

1.3.3.8 Psychological and cognitive state

With respect to psychological state, the approach in the UK Biobank questionnaire has been to assess psychological trait (neuroticism) and mood based on standardized questionnaires, and to record serious life events and medical presentations for psychological symptoms [47]. These areas are considered to be both predictive of future mental health outcomes and complementary to the assessment of cognitive function. While screening tests to assess cognitive function exist, they are time-consuming and generally unsuitable for self-administration. In addition, they have typically only been administered and validated in much smaller and older populations than in UK Biobank. Following wide consultation, a comprehensive review was conducted of brief tests of cognition that can be self-administered, are easily repeatable within a larger cognitive screening battery [48], and have associations with future cognitive decline. Based on this review, paired-associated learning questions to assess global cognition [49] and reaction time tests for touch-screen administration have been developed and refined through piloting to ensure that they provide wide response distributions.

1.4 Background to baseline physical measurements

1.4.1 General approach to prioritisation

The inclusion and exclusion of baseline physical measurements at the assessment for UK Biobank were considered with respect to relevance, reliability and resources. With respect to relevance, the inclusion of a measure at baseline was dependent on other epidemiological studies having indicated that it was significantly associated with health outcomes. For reliability, methods were chosen within a quality assurance framework that involved calibration, maintenance, ease of use, training, monitoring and data transfer to IT systems. Given the large sample size, recurrent costs were considered to be more important than capital costs, and the target for making all of the measurements in the assessment centre was about 20 minutes.

1.4.2 Included measurements

The included baseline measurements listed below were piloted in the integrated pilot (March-June 2006), as well as in the phase 1 pilot. Although there were minor modifications to Assessment Centre procedures between the two phases of piloting, average times for making these measures remained about 20 minutes. Additional measures were considered but excluded following the Phase 1 Pilot experience, chiefly based on the criteria of time available during the assessment (see Section 1.6.4)

1.4.2.1 Blood pressure (and pulse rate)

Blood pressure is a well established cause of coronary heart disease, stroke and several other vascular diseases [50], and, through mechanisms that are poorly understood, may be an important cause of dementia [51]. In addition, blood pressure accounts for a large proportion of the effects of obesity on health, such that a proper understanding of the effects of obesity is not possible without a proper understanding of the effects of blood pressure. Although the average age-specific blood pressure levels of UK adults have fallen in recent years, most UK adults in middle and old age still have blood pressure levels that significantly increase their risk of developing vascular disease [50, 52].

Blood pressure (and pulse rate) will be measured in UK Biobank using the Omron HEM-7015IT digital blood pressure monitor. After correctly applying the blood pressure cuff, staff need only press a button on the monitor before waiting for the cuff to automatically inflate then deflate. Following this, the monitor automatically downloads the systolic and diastolic blood pressure (and pulse rate) readings to the assessment centre IT system. The process is then repeated, to obtain a second set of readings, after the participant has rested for about one minute. The blood pressure measurement process is quick (taking two to three minutes in total, including the one minute’s rest) and simple (requiring minimal staff training and monitoring).

The Omron HEM 7015-T has been recommended for use by the British Hypertension Society. A less technically advanced version (Omron 705CP) has been used in several large studies, including the Anglo Scandinavian Cardiac Output Trial (ASCOT) and the British Genetics of Hypertension (BRIGHT) Study, and it is used routinely in NHS blood pressure clinics. Compared with this earlier version, the Omron HEM 7015-T can automatically download readings to a computer, thereby saving time (and, hence, also staff costs) and reducing the potential for data errors. Despite its technical advantages, the Omron HEM 7015-T digital monitors involve only a modest capital cost, and they will be a source of minor recurrent costs (e.g. each device only needs infrequent recalibration).

Blood pressure levels are known to fluctuate randomly within individuals, which complicates matters if measurements at one visit are to be taken as indicating the "usual" blood pressure levels for those individuals. Importantly, random fluctuations in blood pressure tend to result in individuals having their blood pressure "miscategorised" in such a way that the effects of blood pressure on disease outcomes are systematically underestimated [50, 53]. This "regression dilution" bias can be appropriately controlled by re-measuring blood pressure every few years during follow-up in a reasonably representative sample of participants [53] (as will be done in UK Biobank: see Section 2.5.1). Regression dilution bias for the other measurements detailed below can also be corrected in the same way, although the bias may be less since these other measurements do not fluctuate as much as blood pressure.

1.4.2.2 Weight

Most differences in weight between individuals can be accounted for by differences in height and body fatness. After taking adequate account of height (see below), therefore, weight turns out to be a useful indicator of body fatness [54]. An easy, widely used, and reasonably accurate way of taking account of height is simply to divide weight by the square of height, yielding the so-called body mass index (kg/m^2). Body mass index has been shown to be quite strongly correlated with percentage body fat (i.e. the percentage of body weight accounted for by fat weight) as determined by more sophisticated laboratory methods such as densitometry [55]. For European adults, a body mass index of 25 to 30 kg/m^2 is generally considered [56, 57] to indicate "overweight", and greater than 30 kg/m^2 to indicate "obesity". There is now clear evidence from many sources that a body mass index above about 25 kg/m^2 increases the risks of developing ischaemic heart disease [58], ischaemic stroke [59], type 2 diabetes [60], osteoarthritis [61] and at least four types of cancer (colorectal, kidney, endometrial and postmenopausal breast) [62-65]. The effects of excessive body fat are of growing significance for public health in the UK because adults (and children) are storing increasingly large amounts of body fat: for example, whereas about one in five middle-aged adults in England and Wales had a body mass index greater than 30 kg/m^2 in the early 1990s, now about one in three do [52].

Weight will be measured using the Tanita BC-418 MA body composition analyser, which is described in detail below in Section 1.4.2.6. Staff will ask

participants to remove shoes and heavy outer clothing and then step onto the footpads of the body composition analyser. Staff then press a button to start the analysis, during which weight (and several other variables) are measured. The readings then download automatically to the assessment centre IT system. Measuring weight adds no delay to the bioimpedance assessment, and the body composition analyser is straightforward for staff to use. The analysers represent a moderate capital expense but they are robust (requiring only infrequent recalibration), they accurately measure body weight to within 0.1 kg, and they will also yield other potentially valuable information about body composition (Section 1.4.2.6). Automatic transmission of weight readings to the assessment centre IT system will reduce labour costs and improve data accuracy.

1.4.2.3 Height

The key reason for measuring height is that information on height can substantially improve the value of several other physical measurements. For example, after correction for height, weight becomes a reasonably good measure of body fatness (e.g. as body mass index: Section 1.4.2.2) and certain spirometric measurements (Section 1.4.2.8) assume greater predictive potency. In UK Biobank, height can also be used in algorithms that estimate percentage body fat and other indicators of body composition from bio-impedance (Section 1.4.2.6). Height often needs to be allowed for in epidemiological studies because height is itself an independent predictor of mortality. For example, shorter people tend to have moderately higher risks of vascular diseases [66], and moderately lower risks of neoplastic diseases [67], compared with taller people. The reasons for these associations are not known. Further data on associations with the main components of height (i.e. leg length and trunk length) might improve understanding of how height affects health, and of how best to correct for these effects when considering other variables. Leg length and trunk length can be estimated simply and reliably from standing height by also measuring sitting height.

Standing *and sitting* height (shoeless) will be measured using a *Seca 202 height measure.* Staff will read the measurements off analogue rulers and manually enter the readings into the assessment centre IT system, which will automatically and immediately flag up impossible or implausible values. The process of height measurement takes less than one minute and requires only a little staff training. The *Seca 202 height measure was* recommended (for use with adults) by experts involved in studies of child growth, and will involve only a minor capital expense.

1.4.2.4 Waist circumference

Excessive body fat is known to increase the risks of several common diseases (Section 1.4.2.2) and, in addition, there is considerable evidence that excessive fat stored in the intra-abdominal cavity may be especially harmful [68, 69]. Intra-abdominal fat, which is lipolytically more active than fat elsewhere [70], releases large amounts of free fatty acids into the bloodstream and, because this blood drains directly into the portal vein, the

free fatty acids are transported straight to the liver. When there is a large amount of intra-abdominal fat, the resulting heavy flux of free fatty acids to the liver is thought likely to disturb hepatic metabolism in ways that lower the body's sensitivity to insulin [70], while also disturbing the balance of blood lipids [68], and ultimately raising the risks of developing type 2 diabetes, hypertension and other specific vascular diseases [71-73]. Intra-abdominal fat mass can be inferred reasonably well from waist circumference. Clinical studies have shown that, within each sex, waist circumference is highly correlated with intra-abdominal fat mass estimated by ultrasonography and MRI [74, 75]. (By contrast with weight, waist circumference is only weakly related to height, and typically no height adjustment is required [76].) Furthermore, many epidemiological studies have reported that larger waist circumference predicts higher levels of major vascular risk factors [73], and also a higher incidence of vascular events [72], even after allowing for weight and height (e.g. as body mass index).

Waist circumference at the level of the umbilicus will be measured using a Wessex non-stretchable sprung tape measure that has been used in previous large health studies (including the BRIGHT hypertension study [77]). Staff will manually enter the readings into the assessment centre IT system, which will automatically and immediately warn staff of impossible or implausible values. Measurement of waist circumference typically takes about two minutes as it involves adjustment of some clothing by the participant, and it will involve negligible capital expenditure. However, measuring waist circumference will require a modest amount of staff training and monitoring to ensure that the measurements are done correctly.

1.4.2.5 Hip circumference

There is some epidemiological evidence that larger hip circumference is associated with lower risks of vascular diseases, independently of the effects of weight, height and waist circumference [78, 79]. The reason for these reported inverse associations is uncertain, as hip circumference is determined by poorly understood factors, such as pelvic bone width and amount of gluteal muscle and subcutaneous fat [76]. However, because waist circumference and hip circumference appear to affect vascular disease in opposite directions, the ratio of waist circumference to hip circumference ("waist:hip ratio") could be a particularly informative predictor of vascular risk. The large INTERHEART retrospective case-control study recently reported that waist:hip ratio was a much stronger predictor of incident myocardial infarction than either waist circumference or body mass index [80]. But, the overall body of evidence concerning waist:hip ratio is quite inconsistent [71, 81], and better large-scale prospective evidence is needed to elucidate the real (if any) role of hip circumference in vascular diseases.

Hip circumference will be measured using the same tape measure as for waist circumference (Section 1.4.2.4). As with waist circumference, measuring hip circumference will require some staff training and monitoring, but the process is quite quick (about one extra minute) and involves almost no capital outlay.

1.4.2.6 Bio-impedance

Body mass index and waist:hip ratio (Sections 1.4.2.2 & 1.4.2.5) are both easy to estimate, but each have important theoretical and practical limitations. For example, body mass index makes no allowance for the possibility that, at any given height, a greater weight might be a consequence of more muscle rather than more fat [82]. Waist:hip ratio takes no account of the potentially deleterious effects of fat other than fat in (and over) the abdomen, while assuming that all major factors increasing hip circumference (including fat over the hip) somehow produce beneficial effects. Furthermore, neither body mass index nor waist:hip ratio can address the fundamental question of whether percentage body fat or absolute fat mass (or some similar measure related to body composition) is aetiologically much more relevant to specific diseases. Assessing whole-body bio-impedance provides a straightforward, rapid and reliable way around most of these limitations. Bio-impedance is defined as the opposition in biological tissues to the flow of alternating current, and it is invariably much greater in adipose tissue (which contains little water or electrolyte) than in lean tissue (which is essentially an electrolyte solution). As a consequence, the overall level of impedance in the body can be a good indicator, when combined appropriately with other data (e.g. age, sex, weight and height), of the absolute and relative amounts of adipose and lean tissue [82, 83]. Many cross-sectional studies have shown that body composition estimated by bio-impedance agrees closely with that estimated by more rigorous laboratory methods [83-85]. Bio-impedance has consequently been used widely in clinical studies [86] and in some small or medium-sized epidemiological studies [87-89], but not in many large epidemiological studies [3, 89]. Assessment of body composition in more detail than has been possible in previous large UK studies could yield new insights into the increasingly pressing problem of obesity in the UK.

In UK Biobank, bio-impedance will be measured using the Tanita BC-418MA body composition analyser. This device measures bio-impedance by passing an extremely low, and completely imperceptible, via the trunk, legs and arms [84, 85]. Participants stand briefly in bare feet on the analyser’s footpads, and hold its handles, while measurements of bio-impedance (and weight: Section 1.4.2.2) are made automatically and then downloaded electronically to the assessment centre IT system. This assessment takes about three minutes in total, and will require a modest amount of staff training to ensure that the analyser’s (few) buttons are operated correctly. Tanita are the leading manufacturer of bio-impedance assessment equipment, and there are in-built algorithms for estimating body composition that have been developed in Western populations. This will not, however, preclude researchers from using the raw data on bio-impedance from UK Biobank since both measured and calculated values will be captured. The Tanita analysers represent a modest capital cost, but recurrent costs will be small (e.g. requiring only infrequent recalibration).

1.4.2.7 Hand grip strength

Hand grip strength is a predictor of all-cause and cardiovascular mortality, as well as disability. A cohort study of 6,040 45-68 year old healthy men in Hawaii found that, after 30 years, the lowest tertile of hand grip strength had a relative risk of mortality compared with the highest tertile of about 1.3 [90]. The risk of self-care disability doubled for those with a baseline hand grip strength in the lowest tertile compared with the highest tertile [91]. A smaller cohort of 919 65-101 year old disabled women in Baltimore found that the lowest tertile of hand grip strength had about three times the risk of cardiovascular mortality compared with the highest tertile [92]. In a more recent study of 1,071 men in the Baltimore Study of Ageing, survival analysis over a 25-year period showed that the rate of loss of muscle strength was a more important predictor than baseline strength in men less than 60 years of age, but the reverse was true for men aged over 60 [93]. A recent study of European men and women aged over 50 years found that low hand grip strength was associated with lower bone mass and, for women, increased risk of developing incident vertebral fracture (OR=2.67; 95% CI: 1.13 to 6.30) [94]. An analysis of 1490 men and women aged 61-73 in a Derbyshire cohort found that grip strength was greater on the non-dominant side in about one quarter of individuals (Helen Martin: personal communication).

Right and left hand grip strengths will be measured once each using a Jamar J00105 hydraulic hand dynamometer. The measurement of hand grip strength is dependent on maximal effort by the participant, so staff need to instruct participants how to use the equipment in order to help ensure that maximal effort is obtained. In terms of equipment, maintenance costs and participant's time, grip strength measurements require minimal resources. It takes a total of about two minutes for both right and left hands. Since manual input of data is required, there is the potential for errors within the range of valid values (although the IT system will flag up impossible or implausible values).

1.4.2.8 Spirometry

Although spirometry assesses lung function, it has also been found to be a predictor for death from all-causes, cardiovascular and cerebrovascular disease, as well as chronic lung disease and lung cancer [95-97]. An analysis of the Whitehall Study suggested that height-adjusted forced expiratory volume in 1 second (FEV_1) was a stronger predictor of mortality than height, body mass index or plasma cholesterol, while age-adjusted FEV_1 was almost as strong a predictor as systolic blood pressure [98]. Spirometry is dependent on maximal effort by the participant, which can be detected by comparing the FEV_1 and forced vital capacity (FVC). The American Thoracic Society (ATS) recommends that at least three spirograms should be obtained which are free from artefacts (such as coughs) and that the two largest FEV_1 and FVC should be within 150mL to be considered acceptable. In a cross-sectional analysis among 25,000 people in the EPIC-Norfolk study, however, the better of just two blows provided a population distribution that was closely

associated with other factors, such as obesity [99] and self-reported health [100].

The Pneumotrac Vitalograph and ndd Easyone spirometers were the two leading models recommended by respiratory experts that were consulted. Both machines had been used extensively in observational studies and clinical trials, and fulfilled various key requirements (e.g. conformed to ATS requirements, validated, reliable, robust, easy to use, IT data download). The Vitalograph Pneumotrac 6800 spirometer was chosen chiefly because it performed slightly better in preliminary pilots, and linkage to the assessment centre IT appeared more straightforward. It was decided to make up to three measurements of lung function within a maximum of 6 minutes (since more attempts over a more prolonged period were not considered acceptable for participants). Staff are carefully trained in the conduct of the measures, including demonstration of the use of the equipment to participants, in order to increase the likelihood that two technically acceptable measurements are obtained. Spirometry requires minimal resources in terms of equipment and maintenance costs, but it does involve significant training and participant time. Electronic data capture of the flow curves in the assessment centre IT system allows immediate feedback to staff about the technical quality of the measurements, while also facilitating central validation.

1.4.2.9 Bone densitometry

The assessment of bone mineral density with calcaneal ultrasound has been found to be predictive of hip fracture in both the EPIDOS study of 5,662 elderly women in France [103] and the EPIC-Norfolk study of 14,824 men and women aged 42-82 years in the UK [104]. In both studies, one standard deviation less broadband ultrasound attenuation was associated with a doubling in risk of hip fracture. Calcaneal bone density in the left heel will be assessed using the Norland McCue Contact Ultrasound Bone Analyser (CUBA), which provides a measure of Broadband Ultrasound Attenuation (BUA). While previous studies have measured either one foot or both feet (and, in most instance), simply average the readings from both feet), time constraints mean it is only feasible to measure one foot. A small amount of contact gel is placed on the two transducers, and the participant is then asked to put their foot in the holder and to sit upright with slight pressure on their heel to ensure good contact. Staff will manually enter the readings into the assessment centre IT system, which will automatically and immediately warn staff of impossible or implausible values. Calcaneal ultrasound takes 1-2 minutes (provided the participant remains still), although preparations may increase the procedure time to 3-4 minutes. The analysers do represent a moderate capital expense but they are robust (requiring only infrequent recalibration) and straightforward to use (requiring only a modest amount of staff training and monitoring).

1.4.3 Excluded measures

A number of other measures were considered, but excluded from the core baseline assessment for reasons of feasibility (see below).

1.4.3.1 Electrocardiogram (ECG)

A 12-lead ECG would allow the detection of asymptomatic ECG abnormalities, such as silent myocardial infarction, left ventricular hypertrophy, left axis deviation and ventricular ectopic beats. The Whitehall II Study of London civil servants found that abnormal ECG changes (such as Q waves, ST depression and left bundle branch block) were asymptomatic in about 2% of the population and associated with a two-fold higher risk of all-cause mortality [101]. In the British Regional Heart Study of 7,735 middle-aged men, such ECG abnormalities were predictive of non-fatal and fatal cardiovascular disease [102]. The phase 1 pilot for UK Biobank included a 12-lead ECG which allowed Minnesota coding. But, although the ECG tracing itself took only about ten seconds, preparation time by the participant in removing some clothing and by staff in attaching limb and chest leads extended the measurement time to about ten minutes. A 4-limb ECG would be somewhat quicker to conduct, but most minor ECG abnormalities would not be detected by it. Consequently, given the time constraints for the assessment centre visit, it was decided to exclude an ECG from the standard UK Biobank baseline visit (but see Section 2.5.3).

1.4.3.2 Other excluded measures

Other potential baseline measurements that were considered, but excluded, are: continuous or ambulatory blood pressure and pulse rate; ankle-brachial index; pulse wave velocity; carotid intimal-medial thickness; cardiac echocardiogram; skinfold thickness; spirometry reversibility; quadriceps strength; timed shuttle walk test; aggregated locomotor test; and visual and auditory acuity. Despite their potential association with various health outcomes, time constraints meant that these measures could not readily be included with the other measures in the baseline assessment of the full cohort (although it is intended to seek separate funding to conduct some of them in selected subsets, both at baseline and during repeat assessments: see Section 2.5.3).

1.5 Background to baseline samples

1.5.1 General approach to sample collection

Development of the protocol for the collection of biological samples in UK Biobank was led by a number of key principles. In particular, the aim should be to collect samples that would allow the widest possible range of assays that could plausibly be envisaged for the future, and to avoid collection, processing or storage approaches that would inherently preclude such assays (i.e. "future proof" the collection as far as possible given current knowledge and available resources). The UK Biobank sample handling procedures are the result of extensive consultation and peer review in the scientific community, followed by extensive piloting to ensure that the proposed procedures were fit for purpose [105]. The coordinating centre laboratory Standard Operating Procedures detail the samples to be collected, the preliminary processing and storage temperatures, the transport of samples to a central processing facility, and the processing, aliquoting and storage of each sample (which is summarised below).

1.5.2 Biological samples to be collected

There was extensive consultation and discussion on which biological samples to collect at the assessment centre visit. The inclusion criteria were based on the likely value of the additional information that would be made available by collecting some particular sample type (i.e. the range of assays that could be made and the physiological coverage of the material), and the feasibility and cost of collecting and processing such samples from the 500,000 participants. On this basis, it was decided to collect 40-50 ml of blood and a random urine sample during the baseline assessment visit (see Box 1.5.1).

Sample type	Selection criteria
Blood	• Variety of fractions: plasma, serum, white cells, red cells, peripheral blood lymphocytes<br>• Wide range of biomolecules: DNA, RNA [5' ends], proteins, analytes<br>• Wide physiological coverage: genome, proteome and metabolome, haematological parameters<br>• Suitable for a very wide range of assay technology<br>• Ease and low cost of collection
Urine	• Wide range of biomolecules: proteins, analytes (including pharmaceuticals)<br>• Wide physiological coverage: proteome and metabolome (including gut microbiome)<br>• Suitable for many assay/technology types<br>• Low cost of collection

Box 1.5.1: Included biological samples and rationale

Having decided on blood and urine collection, consideration was given to additional types of sample that might allow measurements of factors not covered by blood or urine (see Box 1.5.2). On this basis, it was decided to exclude all other sample types because they were not considered likely to provide sufficient additional information to characterise participants in ways that would be importantly predictive of subsequent health outcomes. For example, bacterial gut fermentation by-products in faeces are biomarkers of a number of diseases of the gut (such as irritable bowel syndrome and, possibly, Crohn's disease). These markers include hydrogen, methane, alkanes, methyl alkanes, phenols and organic acids, which can also be measured accurately in urine [106]. Furthermore, the gut microbiome can be profiled in urine using NMR approaches. Hair and nails may be used to assess medium-term exposure to heavy metals. But, a study of the toxicokinetics of methylmercury exposure concluded that hair and blood levels are of questionable value as indicators of both body and target organ concentrations of mercury [107]. Moreover, some forms of arsenic (such as arsenobetaine, the major organic arsenic compound in seafood) do not accumulate in hair [108]. In addition, measures of environmental arsenic in hair and nails are influenced by external contaminants (such as air, water soaps and shampoos), and such exposure is better measured in urine [109].

Sample type	Exclusion criteria
Faeces	• Limited additional information (e.g. gut microbiome)<br>• Difficulty in collecting/processing<br>• Potential impact on recruitment<br>• Complexity and cost of storage
Hair	• Limited additional information (e.g. exposure to environmental heavy metals)<br>• Complicating effects of cosmetics and toiletries
Nails	• Limited additional information (e.g. exposure to environmental heavy metals)<br>• Complicating effects of cosmetic products<br>• Inconsistency of sample collection<br>• Possible impact on recruitment of clipping nails
Saliva	• Limited additional information (e.g. indicators of periodontal disease and oral cancer)<br>• Extra cost of storage

Box 1.5.2: Excluded biological samples and rationale

1.5.3 Types of sample collection tubes

There is a very wide variety of preservatives and additives available for the collection of blood and urine. In a review of factors that affect the quality of biomarker assays, the importance of careful selection of anticoagulants and preservatives in the collection tubes was stressed [110]. Certain anticoagulants are recommended for some analyses whilst others are contraindicated. For example, blood collected into EDTA-containing tubes is good for DNA-based assays, but may be unsuitable for others because it

chelates magnesium ions; heparin-stabilized blood affects T-cell proliferation assays and heparin binds to many proteins. EDTA plasma and serum give assay-dependent variation in measures of growth hormone, thyroid stimulating hormone, insulin, C-peptide, total estradiol, testosterone, cortisol and progesterone in fluorometric and immunofluorometric assays [111]. Any anticoagulant may cause *in vitro* induction of cytokines and artefactually elevated concentrations [112]; and addition of borate stabilises urine samples but interferes with some metabonomic assays (Jeremy Nicholson: personal communication). Inevitably, the selection of additives is a compromise, and the choice made for UK Biobank has been made to cover as wide a range of potential future uses as is feasible.

UK Biobank's sample handling pilot studies have demonstrated that maintaining whole blood and urine samples at 4°C for at least 36 hours prior to processing and cryopreservation allows a very wide range of assays to be performed [105]. An additional acid citrate dextrose (ACD) tube of whole blood maintained at 18°C also allows subsequent immortalisation of lymphocytes. Consequently, the processing of collection tubes at the assessment centre can be minimised, and most of the processing conducted at the central laboratory using efficient automated systems. These processing platforms isolate and aliquot multiple fractions from the EDTA tubes to produce fractions suitable for DNA extraction and a wide range of assays using the red cells and plasma. In addition, one gel plasma separation tube (PST) and one gel serum separation tube (SST) will be collected for each participant to protect the plasma/serum from any changes prior to delayed separation that might affect certain assays (e.g. elevation in the levels of potassium and homocysteine).

Type of sample	Collection priority	Volume collected (ml)	Transport temperature (^{o}C)
EDTA	1	9	4
EDTA (PST)	2	8	4
Clot activator (SST)	3	8	4
EDTA	4	9	4
Acid citrate dextrose	5	6	18
EDTA	6	4	4
Urine	-	9	4

Table 1.5.1: Sample collection priority, volume and transfer temperature

The "vacutainer" system will be used to collect these blood and urine samples (see Table 1.5.1). During venepuncture, the hypodermic needle is connected to these vacutainer tubes, which are held under a slight vacuum and contain the required additives, and the vacuum draws sufficient blood to fill them. As a set of the required tubes is collected, unique bar-codes for each tube are scanned into the assessment centre IT system to link each tube with the participant's identifier number. A collection priority is specified in the event that assessment centre staff cannot extract sufficient blood for the full set of tubes in order to provide the widest possible range of different fractions and sources of biological material (see below). A similar system is used to transfer

the participant's urine into a vacutainer from the urine collection vessel. All tubes are maintained at 4°C (with the exception of the ACD tube which is maintained at 18°C) until they are ready for dispatch to the central processing laboratories in temperature-controlled shipping boxes.

1.5.4 Central processing methodology

On an average day, UK Biobank will recruit a total of 600-800 participants in about 6 assessment centres distributed around the UK. This will yield about 5000 separate vacutainers of samples, which will be transported to the central laboratory for further processing. As indicated in Table 1.5.2, the different samples from each individual will yield up to 30 aliquots of 1.4ml volume for long-term frozen storage. The rationale for storing this large number of separate aliquots for each individual is to provide sufficient amounts of each type of sample for a wide range of experiments during long-term follow-up and to protect the samples from repeated rounds of freezing and thawing.

About 20,000 aliquots will be produced in 1.4ml bar-coded tubes each day. This high throughput repetitive work, coupled with the requirement for high quality and secure tracking of samples, has led to the development of highly automated platforms for UK Biobank that are fully integrated with the Laboratory Information Management System (LIMS) software. Some of the liquid handling tasks (e.g. urine) can be managed using customised integrated robotic workstations available from commercial suppliers. The more complex fractionation and liquid handling tasks will be performed on custom-built multi-function automated platforms. Importantly, these platforms do not rely on any "leading edge" technology to function; rather they represent a new configuration of existing robust technologies (which reduces the risk of failure). Only those assays that cannot be done subsequently on samples that have been frozen (i.e. haematology) are to be performed as samples arrive at the central laboratory in order to streamline processing, improve cost-effectiveness and minimise quality control issues.

Vacutainer tube	Fractions	Number of aliquots	
		**-80°C**	**Liquid N_2**
EDTA x 2	Plasma	6	2
	Buffy coat	2	2
	Red cells	-	2
EDTA (PST)	Plasma	3	1
Clot activator (SST)	Serum	3	1
ACD	DMSO blood	-	2
EDTA	Haematology (immediate)	-	-
Urine	Urine	4	2
TOTAL ALIQUOTS		18	12

Table 1.5.2: Fractions and aliquots of blood and urine samples

The different types of sample that are being collected for each participant have different processing requirements in the central laboratory (Table 1.5.2), and will allow a wide range of different types of assay:

- ***EDTA (x2 9 ml vacutainers):*** The different blood fractions will be separated by centrifugation at 2500g for 10 minutes at 4°C. Four aliquots of plasma, 2 aliquots of white cell “buffy” coat and 1 aliquot of red cells will be transferred from each of these two vacutainers to bar-coded 1.4ml storage tubes suitable for long-term cryopreservation. Subsequently, these aliquots can be used for assays of the proteome, metabonome and 5’ RNA fragments in plasma; for purification of large quantities of high molecular weight genomic DNA from the buffy coat; and for assay of red cell membrane lipids and heavy metals.

- ***EDTA (plasma separation vacutainer):*** Four aliquots of this plasma will be transferred to bar-coded 1.4ml storage tubes at 4°C prior to cryopreservation. These aliquots can be used subsequently for assay of the plasma proteome and metabonome when relevant changes (e.g. haemolysis) may have occurred following delayed separation in the standard EDTA tubes.

- ***Serum (serum separator vacutainer):*** Four aliquots of serum will be transferred to bar-coded 1.4ml storage tubes at 4°C prior to cryopreservation. These aliquots can be used subsequently for assay of the serum proteome and metabonome (including those chelated in EDTA plasma).

- ***Acid citrate dextrose:*** Two 500 µl aliquots of whole blood are mixed with two 500 µl aliquots of sterile 20% DMSO (diluted in RPMI growth medium) in bar-coded 1.4ml storage tubes in a laminar flow cabinet, and then transferred to a -80°C environment in insulated polystyrene containers for 16 hours prior to long-term cryopreservation in liquid nitrogen. These aliquots can be used subsequently for immortalization of peripheral lymphocytes with Epstein Barr virus in order to produce replenishable supplies of high molecular weight genomic DNA representative of all genomic regions, as well as mRNA transcripts and splice variants (albeit representative of a B-cell background). They can also be used for functional assays, such as *in vitro* antigen presentation studies, functional genomic studies and cell nuclei transfer studies.

- ***EDTA (4ml vacutainer):*** This vacutainer of whole blood will be mixed on arrival and then will be placed on the automated Beckman Coulter counter for haematological assays (since these cannot be done later on thawed samples).

- ***Urine:*** Six aliquots of urine will be transferred to bar-coded 1.4ml aliquot tubes at 4°C prior to cryopreservation. These aliquots can be used subsequently for assay of the urine proteome and metabonome and, potentially, for characterization of the gut microbiome.

1.5.5 Long-term sample storage

By the end of recruitment, UK Biobank will be storing about 15 million 1.4ml aliquot tubes. As indicated in Table 1.5.2, samples from each participant will be stored in two geographically separate locations in order to protect the resource from loss. One location will house the “working” archive that will typically be used first for any research project and the other location will house the “back-up” archive that will be used when samples in the working archive have been exhausted. At full capacity, the working archive will hold 9 million sample tubes at -80°C, and will use custom-built robust industrial automated processes to facilitate reliable storage and retrieval of samples. The rationale for using an automated working store is based on continuity of storage and robustness of operation, achievable reliability of sample tracking and identification, and sample security. The back up archive will hold 6 million 1.4ml sample tubes in liquid nitrogen vapour (-196°C) in insulated stainless steel tanks that require manual loading and retrieval of samples.

1.5.5.1 Continuity of storage and robustness of operation

Long-term sample integrity is of primary concern, especially since the intended lifetime of the UK Biobank resource is more than 20 years. The most important aspect of this is continuity of storage conditions at the intended temperature. Loss of condition that exposes samples to elevated temperatures (especially if allowed to thaw), would drastically limit their usefulness for future research and could potentially remove all value from the samples. In order to ensure sample integrity, the storage solution is designed to maintain conditions in the event of a range of potential problems (including mechanical failure of the store robotics or refrigeration plant, and electrical supply interruption). The refrigeration and environmental control systems have been specified with a high degree of redundancy. The use of liquid nitrogen as coolant minimises dependency on buildings services and utility supply to maintain conditions, as well as being an intrinsically simple method of cooling. The design of the system is such that a major refurbishment in the future is possible (should it be required) without disrupting the conditions of stored samples.

Over the intended lifetime of UK Biobank, some elements of the automated storage and retrieval system may fail at some stage. It has been designed, however, so that the impact of any likely failure is acceptable, in particular that the integrity of the samples is not compromised and, more generally, that the repository is able to continue to provide, or can quickly resume, its service to users. A Failure Mode Effects Analysis (FMEA) has demonstrated that the automated store provides appropriate response to all likely faults on its operation, to ensure the integrity of the stored samples and the associated inventory model. These include operator errors, robotic faults, power outages, computer hard disc crashes, and component failures in both control systems and refrigeration plant.

1.5.5.2 Reliability of sample tracking and identification

In a manually administered repository it is inevitable that errors will occur over time, leading to loss of samples and loss of sample quality (for example by misplacement of samples, or accidental delays in handling leading to thawing and frosting). Over the long lifetime of UK Biobank, the accumulation of small errors could reduce the ability of a manual system to provide complete sets of samples for research projects. The automated store ensures accuracy of storage and retrieval in two separate ways. First it maintains its own accurate inventory of which samples are where within the store. Second, although the robotics do not rely on the bar-codes on the tubes and racks to achieve automated picking of the correct samples, they do check the bar-codes of each vessel and carrier each time they are moved. This provides 100% verification of identity, and allows vessel movements to be tracked and logged without errors. Assembly of orders for tubes required to fulfil research requests is performed robotically within the controlled environment of the store, without requiring operators to be exposed to low temperature conditions, and none of the samples is ever exposed to a temperature above -20°C until after retrieval from the store.

1.5.5.3 Physical security of samples

Unlike a manual store, the automated system does not require operators to approach the stored inventory, which lies within a locked enclosure to which access is restricted. Operator access to the store user interface is password-protected, with access privileges limited according to user profile. Orders for sample retrieval can only be generated through the LIMS (subject to an approval process), and cannot be instigated by a store operative. Consequently, the physical security of the samples is enhanced by using the automated archive. The separate manual liquid nitrogen store provides protection against physical damage to the automated working store, as well as providing storage at very low temperatures for any analytes that might be affected during prolonged storage at higher temperatures.

1.6 Planning and piloting

1.6.1 Initial decision to establish UK Biobank

The proposal for a large prospective cohort was initially discussed at a meeting in 1999 hosted by the MRC and the Wellcome Trust. It was agreed that developments in biological research by the end of the 20th century provided unprecedented opportunities to improve our future understanding of the environmental and genetic causes of common diseases. Moreover, while several large retrospective case-control studies of the relevance of genes for specific diseases were being undertaken or planned, prospective recruitment and long-term follow-up of a sufficiently large sample from the UK population would allow complementary studies of the separate and combined effects of genetic, environmental and lifestyle causes of a wide range of diseases. It was recognised that the UK was in an ideal position to undertake such epidemiological research, given both its wide health coverage through the NHS and its world-leading researchers in genetics and epidemiology.

Consequently, in June 2000, the MRC and the Wellcome Trust agreed to the principle of developing a proposal for this large prospective cohort. Prof Tom Meade (London School of Hygiene & Tropical Medicine) chaired two expert working groups to develop the concept further. Following discussion with over 150 specialists and a series of public consultations, the working group recommended that a prospective cohort involving around 500,000 UK participants should be undertaken. A protocol was written that provided the overall scientific justification and an outline of the proposed design [113]. Following positive international peer review, the MRC and the Wellcome Trust each agreed to fund the project, and the Department of Health, Scottish Executive and North West Development Agency each subsequently agreed to provide support.

1.6.2 Detailed planning of UK Biobank

In July 2003, the Science Committee for UK Biobank (Chair: Prof John Bell; Oxford) was convened to develop the detailed protocol for UK Biobank Initial working groups were set up during the latter part of 2003 to provide general guidance on various aspects of cohort design (e.g. recruitment; sample handling; follow-up) that would require further development. In particular, the Sample Handling group (Chair: Prof Paul Elliott) considered what samples should be collected, how they should be transported and processed, and the best approach to archiving. It carefully considered a range of potential sample types (e.g. blood urine, hair, nails, faeces etc.) and excluded a number on the basis of limited additional scientific value or feasibility of collection in a non-clinical setting at high throughput (see Section 1.5). Having recommended a detailed sample handling protocol, this group was responsible for the careful and detailed testing of the processes through a series of sample handling pilot studies [105]. UK Biobank also started to investigate options for sample processing and archiving which led to the development of the automated sample handling and storage facilities (Section 2.4).

Subsequently, during 2004-5, expert working groups were established and charged with consulting widely on, and then developing, detailed plans for other specific aspects of UK Biobank. These groups initially aimed to identify a wide range of options within each specific topic area, and then refined these down to recommendations for achieving the best scientific outputs within the available budget. The key activities of these working groups are summarised below:

- ***Recruitment (Chair: Prof Alan Silman; Manchester)*****:** Consider different approaches to identifying and recruiting potential participants from the general population (see Section 2.2);

- ***Questionnaire (Chair: Prof Valerie Beral; Oxford)*****:** Coordinate the development and refinement of questions to be asked of participants and of the ways of obtaining such information (see Sections 1.3 & 2.3)

- ***Measurements (Chair: Prof Paul Elliott; London)*****:** Consider the physical measurements that could be undertaken during the baseline assessment and the equipment to use (see Section 1.4)

- ***Ethnic minorities (Chair: Prof Mark Caulfield; London):*** Recommend strategies and initiatives to recruit participants from ethnic minorities and other potentially hard-to-reach group (see Section 2.2)

- ***Environment (Chair: Prof David Coggon; Southampton):*** Consider approaches to assessing key environmental and occupational exposures via the questionnaire, biological samples and health care records used for long-term follow-up (see Sections 1.3 & 1.5)

- ***Diet (Chair: Prof Stephen Palmer; Cardiff):*** Consider feasible approaches to the baseline assessment of diet using questionnaires and biological samples (see Sections 1.3 & 2.5)

- ***Cognitive function and psychological status (Chair: Prof John Gallacher; Cardiff):*** Consider feasible approaches to the assessment of cognitive function and psychological status using at baseline (see Section 1.3)

- ***Longitudinal follow-up (Chair: Prof Mike Pringle, Nottingham):*** Consider approaches to the longitudinal follow-up of participants through existing and future NHS record systems available in England, Wales and Scotland, as well as issues related to data quality and validation (see Section 2.6)

Inevitably, such decisions were contingent on the outputs of other groups, and integration of all of these recommendations was a key role of the Science Committee. Based on the recommendations of these working groups, and guided by the Science Committee, representatives of the Regional Collaborating Consortia developed more detailed plans for recruitment and baseline assessment which were then piloted (see below).

1.6.3 Development of participant materials

The key ethics and governance principles relating to UK Biobank are laid out in the Ethics & Governance Framework (EGF). This was first prepared by the project funders (the Medical Research Council, Wellcome Trust and Department of Health) with the advice of an Interim Advisory Group on Ethics and Governance (IAG), chaired by Dr. William Lowrance (Geneva) and with members expert in research ethics, philosophy, law, science, social science, and consumer representation. The Group's deliberations were informed by an ethics consultation workshop in April 2002 and general consultation during 2003 on an earlier draft of the EGF with a wide-ranging group of experts and stakeholders, including members of the public, special interest groups and health-care professionals. The EGF (see www.ukbiobank.ac.uk) has been modified in the light of the developing plans for recruitment and follow-up, and the revised draft has been adopted by UK Biobank with the agreement of the funders and the independent Ethics & Governance Council (EGC: see www.egcukbiobank.org.uk). The participant materials (i.e. letters of invitation, information leaflets and consent form) have been developed with the advice of the EGC and in accordance with the key principles in the EGF.

In addition, two focus groups drawn from the general population were brought together during the summer of 2005 to provide feedback on possible approaches to recruitment and, specifically, to inform the design of the participants materials for invitation and consent. The main findings of these focus groups are summarised below:

- Invitation letters need to be immediately distinguishable from other unsolicited mail by clearly conveying that the person is being invited to participate in a major medical research project (as opposed to being asked to make a financial donation to a charity) that has the backing of the government (most notably, the support of the NHS);

- It should be made explicit that participation is entirely voluntary, what taking part involves, and that the benefits are most likely apply to future generations. Participant information leaflets should be in clear language, with a free telephone service available for any questions or concerns;

- Assessment centres should be conveniently located with flexible opening times (including evenings and weekends for working people);

- GPs should be informed that their patients are being invited to participate, but their day-to-day involvement should be minimal to avoid diverting scarce resources away from patient care.

Based upon this (and other) consultations, participant materials for the integrated pilot phase were developed and then approved by the North West Multicentre Research Ethics Committee (MREC) in January 2006 for the integrated pilot (see Section 1.6.4). Subsequent minor amendments to these materials have been submitted and approved by the MREC as a result of the

feedback received (see below). Feedback on the materials was analysed from a number of sources, including:

- Telephone calls to the freephone service, where staff recorded the type of questions asked by callers (as well as referring more complex questions to senior members of the UK Biobank team);

- Postal reply forms returned in the prepaid envelope provided, which also allowed invitees to record their reasons for not participating;

- Letters and e-mails received at the coordinating centre as a result of the invitation mailing;

- Random sample of 10% of participants sent a short questionnaire survey to get their feedback on the participant materials, their baseline assessment visit and their understanding of the consent given.

Based upon analysis of this feedback (and the advice of the EGC), the invitation letter and information material for the main phase of recruitment have been modified to make it clearer that:

- UK Biobank only has limited information on people for the purposes of inviting them, and that the invitation procedures comply with the Data Protection Act;

- UK Biobank has no access to medical information, and an apology is included in case the letter arrives at difficult time (e.g. when seriously ill or bereaved);

- Participation is entirely voluntary and the appointment is only provisional and can be easily changed/cancelled (or ignored);

- Travel expenses can be reimbursed at the end of the assessment visit;

- Feedback of information will not include any measure of blood and urine samples, which will chiefly be analysed in subsequent decades.

Moreover, in order to reduce the bulk of the initial invitation mailing to potential participants and help improve attendance rates, a confirmation letter is sent to those people who agree to attend an assessment centre appointment.

1.6.4 Piloting for full-scale recruitment

Methods for the identification, invitation and assessment of participants were developed following extensive consultation with leading groups in the UK and internationally (see above). In order to determine the feasibility of the planned approach, two phase of piloting were conducted in 2005 and 2006. The first small-scale phase 1 pilot study was intended to test a subset of the key parameters for the assessment visit which would then allow the full protocol to be tested in a much larger integrated pilot phase.

1.6.4.1 Phase 1 pilot study (February-March 2005)

The phase 1 pilot study was conducted between February and March 2005 in each of the six RCCs, and involved a total of about 300 participants. The chief aims of this phase were to evaluate and refine the assessment visit (especially estimating the duration of the various components); to investigate the utility of administering the questionnaire in a touch-screen format; to assess the feasibility of a broad range of physical measures; and to gather qualitative information about the visit from the participants. This pilot did not assess processes for identification and invitation of participants, collection of biological samples or high throughput systems for assessment centre visits.

Despite its relatively small scale, the phase 1 pilot produced a lot of useful information that has helped refine the final protocol:

- ***Visit duration***: The questions and measurements included in this phase of piloting took over two hours. It was agreed that this would need to be reduced (see below) in order to be able to conduct recruitment within the available budget and not deter potential participants;

- ***Questionnaire*****:** Administration of the questionnaire in a touch-screen format was highly successful both in terms of qualitative feedback from the participants and also in terms of speed, accuracy and internal validation. Feedback from participants helped to identify some questions that required clarification or that were redundant, and detailed timings helped to direct the shortening required to reduce the overall visit length;

- ***Physical measures:*** Valuable information was obtained on the various physical measures, including the time taken to complete each measure and data on reliability and reproducibility. Two specific issues were identified. First, although the spirometry equipment performed well, intra-subject measures were highly variable and many failed the quality requirements. This highlighted the need for improved staff training on spirometry, as well as greater integration of the assessment centre IT systems for real time validation of the flow curves. Moreover, in attempting to provide the three acceptable spirometry measures that had been sought, some participants became unduly fatigued and the visit duration was extended unacceptably (for up to 15 minutes). It was decided, therefore, that a maximum of three blows within 6 minutes would be sought in the main study. The second major output on physical measures from phase 1 piloting was the decision to exclude an electrocardiogram because it took up to ten minutes due to participants having to undress (which also caused some embarrassment).

- ***Fasting:*** Participants in the phase 1 pilot were asked not to eat or drink (except plain water) for up to four hours before their visit. Self-reported compliance was high, but many participants volunteered that they found fasting to be inconvenient and uncomfortable, especially for late morning and afternoon visits. Consequently, it was decide to assess the impact of a fasting request more systematically in the integrated pilot.

1.6.4.2 Integrated pilot study (March-June 2006)

The integrated pilot was conducted during March to June 2006, and involved recruitment of about 4,000 participants from the South Manchester area in one assessment centre in Altrincham. The operational objectives of this integrated pilot were to assess all of the planned procedures (i.e. identification, invitation, consenting and assessment of potential participants; data/sample collection, transfer and storage) prior to starting full-scale recruitment. Other objectives included determining the response rate to invitation, as well as any major factors that affected it, and assessing participants' views on the baseline assessment visit and an evaluation of their understanding of the consent to participate (see Section 1.6.3). The integrated pilot study showed that the centralised approach to participant identification, invitation and assessment works well (see Sections 2.2 and 2.3). Information from the integrated pilot has been used to refine the invitation and assessment procedures for the present protocol.

Experience in the integrated pilot is described in detail in a separate report (see www.ukbiobank.ac.uk). Key findings from it include:

- ***Identification of invitees:*** Despite having all necessary ethics and data protection approvals, the ease of obtaining contact details for invitations varied widely between the four different Primary Care Trusts (PCTs) that served the area around the pilot assessment centre in South Manchester. Contact data were obtained from one PCT within a few days of requesting them, and were used for the first rounds of invitations. But, data from the second PCT were obtained only after several rounds of communication with various data controllers, and they could not be obtained at all from the other two PCTs despite repeated requests over a period of some months. This finding re-enforces the value of access from a single database, which was proposed by the Department of Health for the main study.

- ***Appointment system:*** Nearly 60,000 people identified from local PCT registries were invited to participate with a pre-booked provisional appointment. Very few of these people raised any concerns about being contacted in this way or about being offered a provisional appointment (which was confirmed by about half of the attendees). Indeed, the easy availability of the freephone information and appointment service, which was able to provide rapid responses to questions (as well as confirm or change appointments), was frequently commended by invitees. For every 100 people invited, 15 responded by post (of whom 3 attended) and 11 responded by telephone (of whom 7 attended) with an average call length of 4 minutes. The availability of early morning, evening and weekend appointments helped working people to attend, and participant feedback led to married couples in the same household being sent appointments for the same date and time. Review of common questions systematically logged by the telephone service (as well as the few more material concerns raised) has informed the small number of amendments made to the invitation and consent materials (see Section 1.6.3).

- ***Invitation scheduling:*** Valuable experience and information were gained about the phasing of the mailing programmes. Specifically, as the pilot progressed and larger numbers of people were being invited to the assessment centre, the mailing pattern moved from every 2-3 weeks to every week. This allowed greater flexing of the numbers of invitees (to reduce or increase invitations for certain periods) and gave greater control over the different mailing programmes (see Section 2.2). It also meant that calls to the participant information centre were smoothed, which avoided excessively high demand periods with delays in answering calls. Although out-sourcing the call centre was considered, the pilot study confirmed the value of basing it within an academic environment using staff with experience of such studies. This ensured a standardised approach to call handling and provision of information, and an agreed and accessible escalation procedure for more complex enquiries. Based on the pilot experience, therefore, the information centre for the main phase of recruitment has been established within the Welsh RCC.

- ***Response rates:*** One of the key variables relating to participant recruitment and project costs is the response rate. Overall, about 10% of invitees attended the assessment centre. People living within a 2-10 mile radius of the assessment centre were invited, but few could be invited from within 2 miles because contact details from the relevant PCT were not available (see above). Although there was no evidence of different rates of response by distance between 2 and 10 miles, it seems likely that there might be somewhat higher response rates for those living within 2 miles. No differences in response rate were seen by age, but there were slightly higher rates among women (which can be compensated for by central invitation of a slightly higher proportion of men and by maintaining the availability of appointments outside work hours). It is anticipated that response rates can be increased in the main phase of recruitment not only by inviting people living immediately adjacent to assessment centres but also by increased local promotion.

- ***Assessment centre layout:*** In the main phase of recruitment, a mixture of academic clinical research facilities and serviced offices will be used for the assessment centres. Assessment centres will be located with good transport links so that they are convenient for participants to attend (see Section 1.7). There was already a lot of experience within the RCCs of using clinical research facilities, and it was agreed that the serviced office model should be tested in the integrated pilot. A commercial office space provider was identified in Altrincham (South Manchester) and 1800 square feet of serviced space was procured on a short lease. The assessment centre equipment was established using freestanding partitioned booths that were designed to be robust, to provide privacy for participants, and to be relatively easy to assemble and dissemble. Based on initial experience, dedicated seating areas were set up by each of the sequential stations (see Section 2.3) in order to assist the ordered flow of participants through their visit. It was found that the space available for the integrated pilot was about 400 square feet less than would have been ideal. In particular, more

space was required to allow for reception and waiting areas between stations, to make the assessment centre less cramped, to improve privacy, and to accommodate dedicated urine collection facilities. Some problems were encountered with room temperatures due to the lack of built-in air cooling and, very occasionally, with the building's internet connections (which identified the need for back-up cooling and data transfer systems for the main study). Importantly, the serviced office model was shown to work, which increases the flexibility available to UK Biobank for convenient location of assessment centres.

- ***Assessment centre flow:*** In order to achieve enrolment rates of over 100 participants per day in each assessment centre (while also maintaining participant satisfaction) it is essential to optimise flow through the assessment visit. The visit model evaluated in the integrated pilot generally worked well, and was shown to allow more than 100 participants to be seen each day by about 12-13 specially trained nurses, healthcare technicians and clerical staff (but see below for ideal staffing level). Some issues arose with the length of the visit and with delays at certain parts of the visit (and information about the slightly longer visit than originally anticipated was corrected in revised invitation material). The average duration for the first 1,000 participants was around 100 minutes, and refinements were introduced in order to reduce this to about 90 minutes. It was considered important that it be clear both to participants and staff where they should be at any time during the visit, with a simple system to avoid people progressing "out of turn". Several small changes to the lay out and sequence of the assessment were implemented in light of the experience from the early attendees. For example, the blood collection and exit stations were combined to avoid participants who had finished their visit being unclear when and how to leave. Additionally, it was observed that the original approach of collecting urine samples at any of several points during the visit caused confusion. Instead, therefore, this too was added to the blood/exit station, when participants would be given a urine collection pot and asked to provide a sample before leaving to the sample processing technician. Further changes for the main phase of recruitment include: removal of extra cognitive function tests from the interview (and, ideally, incorporating them in the touch-screen system); ensuring that the rest period between blood pressure measures is used to complete the interview; combining the physical measures and spirometry stations; and extending the assessment centre IT system in a number of ways (e.g. validation checks on spirometry to reduce the number of blows needed; alerts to the sample processing station when samples have been collected and are ready for processing).

- ***Assessment centre staffing:*** A major cost of recruitment relates to staff in the assessment centre, and a balance must be struck between the cost and skill levels of the staff to ensure the appropriate quality of the assessment visit within the available budget. Initially the staffing mix in the integrated pilot predominantly involved nurses, but a more cost-effective mix with more healthcare technicians and clerical staff was subsequently found to work well. Thirteen full time staff are required to be on duty

throughout each centre's opening hours to cover all of the visit stations (see Section 2.3.1). However, the pilot found that high participant throughput was more readily achieved if an additional senior staff member was present to ensure participants move smoothly through the visit, to direct staff to address short-term bottlenecks, and to conduct any of the stations when required during busy periods. Consequently, this post has been included in the plans for the main phase of recruitment.

- ***Other aspects of assessment visit:***

    - *Consent:* At least one member of staff was always available to answer any questions that potential participants had about taking part. No problems were encountered with using the touch-screen format for seeking consent, and the electronic signature pad worked well. Initially, participants were offered the choice of a touch-screen format or keyboard/mouse, but no participant had difficulties with the touch-screen format.

    - *Questionnaire:* Analyses of the data found that almost all of the questions provided good response distributions, with very high levels of completion (i.e. few selected "do not know" or "prefer not to answer" options) even for potentially sensitive questions about sexual history, and with good internal validity. Anticipated distributions of responses were recorded for the psychological and neuroticism scales. The cognitive tests on the touch-screen format also worked well; and, at a qualitative level, participants found them enjoyable and easy to perform. Three tests were included: a visual memory (pairs) test, a visual memory (windows) test and a reaction time (snap) test. In addition, there was a word fluency test during the subsequent interview. Following analysis of the data from these tests, it was agreed that some redundancy could be removed without compromising the value of the cognitive function data.

    - *Spirometry:* Significant intra-subject variability in the spirometry measures had been observed in the phase 1 pilot. Consequently, a standardised staff training programme was implemented in the integrated pilot, and data capture was supported by improved IT systems to allow staff to assess the quality of each participant's procedure. Analysis of spirometry data from the integrated pilot by Nigel Clayton (Chief Physiologist at the North West Lung Centre in Manchester) indicated that it was of high quality.

    - *Fasting:* People invited to participate in the integrated pilot were randomised to either being asked to fast for 3-4 hours or not. This did not have much impact on the average response rates, but nor did it have much impact on the reported hours from last meal (i.e. a median of 4-5 hours in each case). On the other hand, a number of participants allocated to "fasting" indicated that it was inconvenient or unpleasant, staff found that it was related to certain problems (e.g. dizziness during spirometry; more difficult blood collection) and, in a

few instances, people with diabetes fasted for potentially serious periods (despite being explicitly advised not to do so). Consequently, it was decided not to ask potential participants to fast prior to the assessment visit in the main phase of recruitment.

- ***Assessment centre management:*** Even though only one assessment centre was run in the integrated pilot, a number of day-to-day issues arose that showed the need for clear management structures. Consequently, a clear problem escalation protocol has been implemented for the main phase of recruitment: the senior member of staff at the assessment centre is responsible for either addressing the issue or escalating it through to an assessment centre administrator based in the coordinating centre (see Section 1.7). Depending on the nature of the issue, the centre administrator will direct it to an appropriate person for resolution. In the integrated pilot, supply of consumables and servicing of equipment was reactive in its approach; for example, staff would contact the coordinating centre only when supplies were running low. Because of the geographical proximity, supplies or new equipment could be easily and quickly transported to the assessment centre in the integrated pilot. But, in the main study, this will not be possible with 5-8 geographically distributed centres operating at any time. Therefore, standing orders for consumables and regular servicing schedules will be established using central systems to ensure consistency and budgetary control of these processes (see Section 1.7).

- ***Laboratory processes:*** Blood and urine samples collected from the participants in the integrated pilot were picked up by the courier in the late afternoon for overnight transport to the central processing laboratories (as would occur in the main study). In the main phase of recruitment, processing of samples at the required throughput and quality will be a highly automated process. But, although much of the sample processing for the integrated pilot was manual, it still allowed many of the laboratory process and systems to be successfully tested:

  - *Validation of laboratory processes and systems:* When the participant samples arrive at the coordinating centre laboratory they are logged into the Laboratory Information Management System (LIMS). The samples in the transport containers must match the sample identifiers expected from the assessment centre. This process generally worked well in the integrated pilot although, in the first weeks, some issues were identified (e.g. logging empty tubes) that have required modifications to the assessment SOPs, training and IT systems.

  - *Validation of manual processes for back-up in the main study:* If one of the automated processing platforms breaks down in the main study, the systems have been designed so that two platforms can cope with the throughput of samples for short periods. In the unlikely event that two processing platforms are out of commission, the integrated pilot has shown that the manual processing approach is

robust and could be implemented for short periods until automated capacity is restored.

- *Validation of automated processing approaches:* Some of the more straightforward liquid handling tasks in the sample processing protocol (urine and haematology) were carried out on the robotic workstations in the integrated pilot. These performed at the expected accuracy and throughput.

- *Validation of the LIMS:* The integrated pilot showed that automated interfacing and validation of data from robotic workstations worked well with no problems encountered. The in-built process validation prevented human-related errors in data transcription.

- *Sample archiving and logging:* Until the automated sample archive is commissioned in 2008, all samples will be stored in manual liquid nitrogen archives. Samples from the integrated pilot were transferred to liquid nitrogen, and hand-held data logging systems used to record the samples in the archive inventory. Subsequently, these hand-held devices were interfaced with the LIMS with 100% accuracy and all data records updated.

Experience in the integrated pilot phase of recruitment into UK Biobank has resulted in modifications to the procedures for the main phase of recruitment, which are described in outline in Section 2 of this protocol and in detail in the relevant Standard Operating Procedures. These procedures were subjected to detailed scrutiny in mid-2006 by the Wellcome Trust's Study Design Expert Group, the independent Ethics & Governance Council, and a specially convened International Review Panel (as well as other referees). The International Review Panel was explicitly asked to provide advice and recommendations on the scientific plans and the study design, amongst other things (such as the international competitiveness and public health value of the planned resource). It unanimously recommended that full scale recruitment should be launched without delay, and the study funders have confirmed funding for the recruitment phase of the project (with the understanding that the follow-up phase is likely to require continuing funding).

1.7 Assessment centre planning

1.7.1 Background

When participants agree to take part in UK Biobank, they will visit an assessment centre near to them for collection of the baseline information, physical measures and biological samples. Over the course of the study, UK Biobank will operate about 35 assessment centres located around the UK. Each centre will be open for about 6 months before it is closed, and a new centre opened in a different part of the country. Identification, commissioning and operation of these assessment centres will be a major part of the activity of the coordinating centre during the main recruitment period. Assessment centres will be identified against three criteria:

- ***Proximity of eligible population:*** Sufficient population will need to live within a convenient distance of the 35 centres (e.g. up to 10 miles radius, as in the integrated pilot in Altrincham, or equivalent in travelling time for other locations) in order to recruit 500,000 people.

- ***Assessment centre location:*** Assessment centres will be located in either clinical research facilities or serviced office space provided by commercial organizations. Whichever type of facility is chosen, it must have good transport links and proximity to parking.

- ***Assessment centre configuration:*** Although the layout of the various stations in the assessment centres is flexible, the premises must have a default level of services (e.g. adequate space, dedicated lavatories)

These three elements will dictate the location of the assessment centres throughout the main recruitment period. Each is covered in more detail in the following sections. When the centres have been identified and established, they will need careful management to ensure optimal operation of the assessment centre and a satisfactory visit experience for the participants (and this too is discussed below)

1.7.2 Proximity of eligible population to assessment centres

Assessment centres will be located in areas with a sufficient population aged 40-69 living within 10 miles (on average, for a centre to be feasible, there will be about 150,000 eligible people within the target area). The integrated pilot in Altrincham showed little difference in response rates out to a distance of 10 miles, but this may vary in the main phase of recruitment between different regions depending on local transport links. Based upon these conditions, a geographical modelling exercise was undertaken to determine the number of people aged 40-69 living within 10 miles of 35 potential assessment centre locations (based partly, but not exclusively, on towns associated with UK Biobank's RCCs). This analysis was undertaken, using GIS mapping based on data from the 2001 census, by the Small Area Health Statistics Unit of Imperial College, London (part of the London RCC). The location of each of

the 35 potential centres was optimised to maximise the number of people aged 40-69 within a radius of 10 miles without overlapping (Figure 1.7.1).

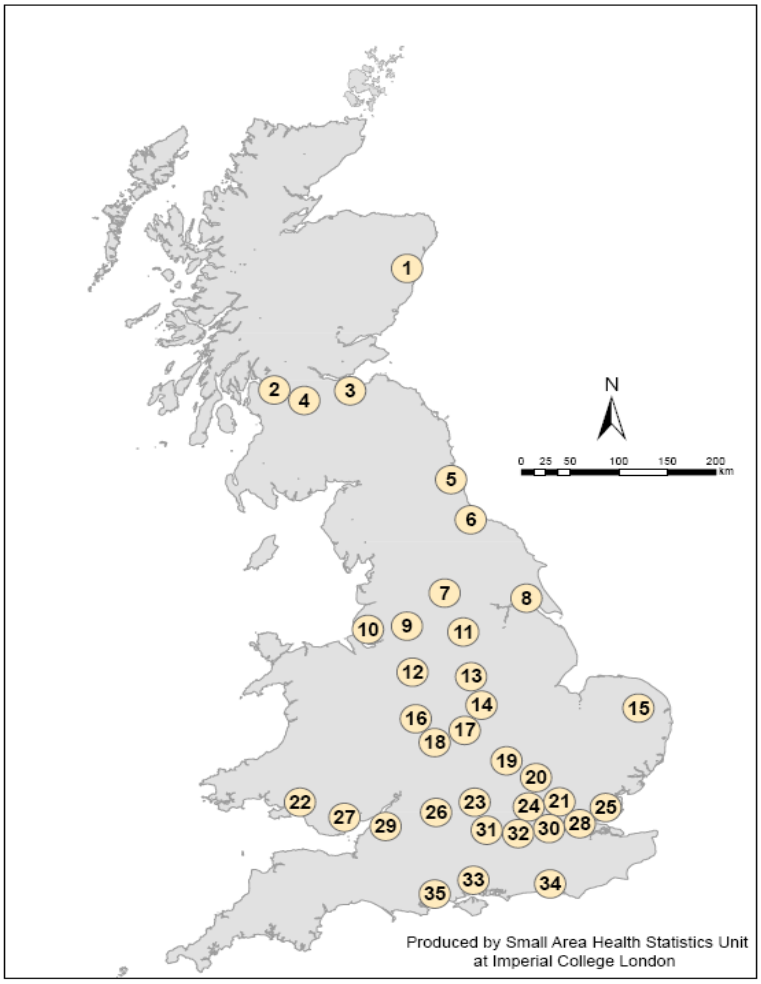

Figure 1.7.1: Locations of 35 potential assessment centre locations determined using GIS mapping with non-overlapping regions of high density populations of eligible individuals

Table 1.7.1 gives a detailed breakdown of the population in 5-year age bands within ages 40-69 for these potential centre locations. In this analysis, there are approximately 10 million eligible people within 10 miles of the potential assessment centres, suggesting that recruitment of the cohort in these locations is feasible. (Although this may well be an over-estimate because 10 miles would be too far for convenient travel in large cities, it still confirms the feasibility of the strategy since a population of 5 million would suffice at 10% response rates.) The actual location of assessment centres for the main phase of recruitment will be more precisely informed by GIS mapping. In addition to overall population density data (as presented above), other key demographic factors will be factored into the model, including practical considerations (e.g. ease of access via public and private transport) and the potential to recruit certain hard-to-reach groups (e.g. deprived populations, ethnic minorities), to help determine the ideal location of assessment centres for recruiting a widely generalisable population.

	% of eligible population in various age ranges						
Map number	40-44	45-49	50-54	55-59	60-64	65-69	Population aged 40-69
1	22	20	20	14	13	12	91,836
2	21	18	18	15	14	13	299,323
3	21	18	19	15	13	13	190,544
4	21	18	18	15	14	13	212,581
5	20	18	19	15	14	13	401,717
6	20	18	19	15	14	13	193,449
7	21	18	19	15	14	13	361,486
8	20	18	20	15	14	13	143,114
9	20	18	19	16	14	12	659,711
10	20	18	19	15	14	13	452,680
11	20	18	19	16	14	13	340,402
12	19	17	20	17	14	13	189,429
13	20	18	20	16	14	13	339,784
14	21	19	19	15	14	12	205,313
15	18	18	20	16	14	13	104,412
16	19	18	19	16	15	13	450,636
17	20	18	19	17	14	12	213,405
18	20	19	19	16	14	12	269,405
19	23	20	21	15	11	9	130,267
20	22	18	19	15	13	12	173,385
21	22	18	19	16	13	12	366,073
22	20	17	19	16	14	13	124,576
23	21	19	19	16	13	12	101,596
24	21	19	19	15	13	12	370,936
25	19	17	21	17	14	12	173,888
26	22	19	19	16	13	12	88,963
27	20	18	20	16	14	12	252,118
28	21	18	20	16	13	12	396,445
29	20	18	19	16	14	12	239,386
30	24	19	19	15	13	11	1,338,611
31	22	19	20	16	13	11	137,973
32	22	19	20	16	13	12	312,648
33	20	18	20	16	13	12	222,549
34	20	18	19	16	14	13	162,779
35	18	16	19	17	15	15	178,678

Table 1.7.1: Population aged 40-69 living within a 10 mile radius of 35 potential assessment centres locations determined using GIS mapping

1.7.3 Assessment centre location plan

It is planned that recruitment for the main phase of UK Biobank will start at the beginning of 2007, and that a new centre will open each month until a steady state of six is reached by around the middle of 2007. Assessment centres will generally run for an average of about six months (depending on population density, local transport links, etc) before being relocated to the next scheduled recruitment area. The first phase of centres is to be sited in cities related to the scientific leads for the 6 RCCs. When possible, the phasing of subsequent assessment centres will be geographically grouped in such a way as to allow trained staff to transition from an assessment centre that is closing to a nearby one that opens.

This assessment centre roll out plan should achieve recruitment of the full cohort of 500,000 people (and re-assessment of 25,000) by the end of the second quarter of 2010. In Figure 1.7.2, the three and a half year recruitment

period is shown on the X-axis, with estimated recruitment figures in dark blue and the cumulative number of assessment centres in light blue. Also shown are the installation and commissioning dates for major capital items (such as the -80°C and the liquid nitrogen archives required to store participant samples) and the annual reviews of progress and plans by the International Scientific Advisory Board (ISAB: see Section 2.9 and Annex 1).

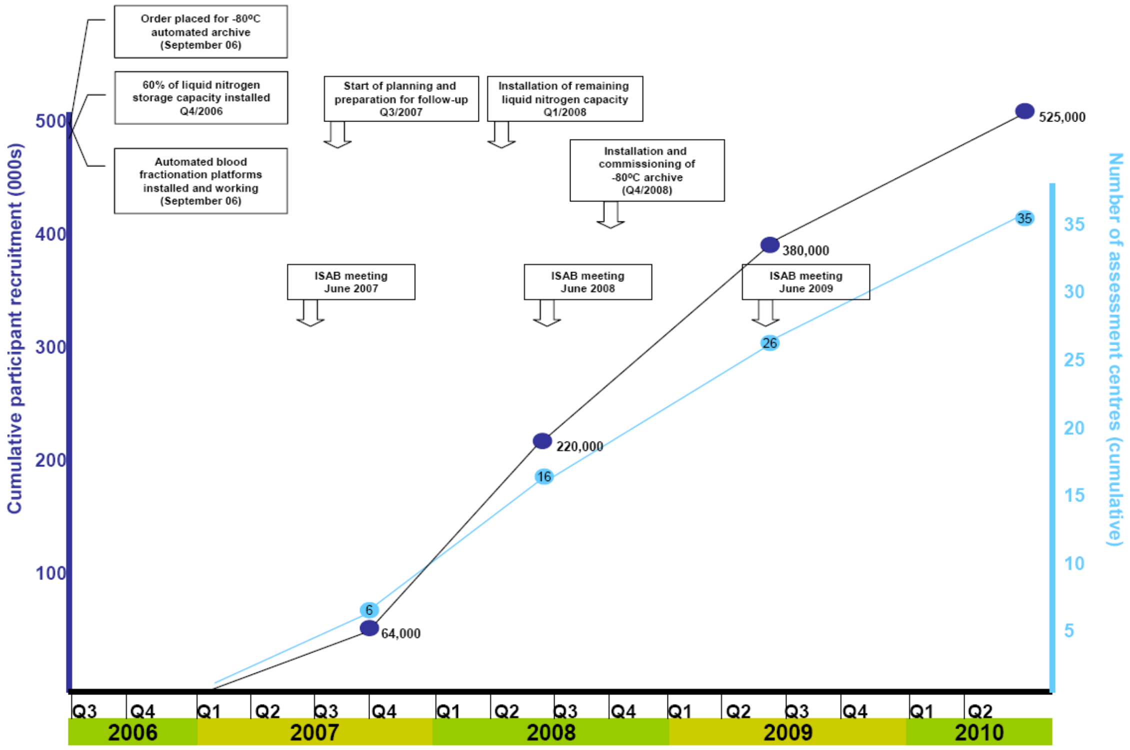

Figure 1.7.2: The targets for the UK Biobank study recruitment period

1.7.4 Assessment centre configuration

When a geographical location has been identified based on eligible population density, a suitable assessment centre facility needs to be established. The UK Biobank coordinating centre team will be responsible for sourcing and securing each of the assessment centre facilities that are required. A mixed facilities model is intended: where suitable cost-effective academic facilities are available then these may be used, but otherwise commercial space (as in the integrated pilot) that meets the requirements specification will be rented on a short-term lease from a serviced office supplier.

This section specifies the requirements for assessment centres, but is not intended to be an absolute specification (particularly since many of the utilities listed can be upgraded or retrofitted). When comparing several options, however, consideration will be given to the availability of the specified utilities at each site. Open plan facilities can be sub-divided by mobile partitions to create the necessary consulting booths. Various aspects of the “ideal” assessment centre are detailed below.

1.7.4.1 Space

- 1800-2200 sq ft (subject to specific configuration)
- Open plan or suitably divided consulting rooms
- Dedicated reception area
- Convenient lavatory area for urine sampling:
  2 x cubicles male
  2 x cubicles female
  1 x disabled cubicle

1.7.4.2 Accessibility

- Good local transport links (bus/rail/road)
- On site parking or nearby car park (within 500m)
- Ideally ground floor (if not, then lift) with disabled access
- Unrestricted evening and weekend access
- Cleaned outside assessment times (i.e. before 8 am or after 8 pm)

1.7.4.3 Other services

- Air cooling (14 KW heat extract capability for 2000 sq ft) or option for installation of portable device
- Tea/coffee making facilities, and area to site drinking water dispenser
- External area for clinical waste bins
- Accessible location for courier pick-up/delivery

1.7.4.4 Power and IT requirements

The following guidelines will be used to assess a potential site in terms of mains power and networking capability (i.e. allowing connection of all assessment centre computers and printers, and providing a suitable connection to the internet). If the space selected does not have a suitable power and network infrastructure then the information below can be used to specify what would need to be installed by UK Biobank staff or contractors:

- There are approximately 69 pieces of equipment that require a standard 3 pin 240 volt plug socket. A minimum of 25 power sockets

would allow safe use of multi-block power extension leads to provide the required number of power sockets

- An internet connection of 1 megabit per second is required. If no phone line is installed, this can normally be installed by British Telecom within a week and a high speed internet connection service provider used.

- Internet connection must be fire-walled from outside world and other building users. If the internet connection is shared with building users then, as well as a firewall on the main connection to the internet (to stop external attacks), a firewall will need to be put in place on the assessment centre system (to prevent internal attacks).

- Secure space for locating a small server, preferably air-conditioned. This could be a designated space within the assessment centre (e.g. the manager office or store room.)

1.7.5 Central management of assessment centres

1.7.5.1 Role of Assessment Centre Administrator

The Assessment Centre Administrator, based at the UK Biobank coordinating centre in Cheadle, will be responsible for coordinating the identification of appropriate premises, recruitment of appropriate staff for each assessment centre, and liaising with the Clinical Operations Manager (see Section 2.3.2) regarding the appointment, training and subsequent monitoring of staff. Staff will be recruited through vetted nursing and related healthcare staff agencies in accordance with the budgeted staffing mix required to carry out the baseline assessment (Section 2.3). Nominated senior nursing staff will be appointed as the centre manager for each shift and be responsible for overseeing the efficient operation of the assessment centre.

Day-to-day issues will be reported by the senior managing staff in the assessment centre to the Centre Administrator, who will deal with the issues directly or forward them to the appropriate person. If the issue is not resolved effectively, the duty operational director will be notified and be responsible for the rapid resolution of the issue. There will also be a documented out-of-hours escalation process for dealing with issues that arise at weekends and outside normal office hours. An issues log will be created and periodically reviewed by the coordinating centre and training/monitoring team. Where recurrent issues can be resolved by changes to the processes then this will be implemented.

1.7.5.2 Commissioning and decommissioning

The Centre Administrator will be responsible for coordinating the commissioning and decommissioning of assessment centres as required for the recruitment plan. It is anticipated with the correct team, planning and management that a new assessment centre can be established in five working days. Decommissioning will take two working days. A project plan specifying the specific tasks, human and physical resources, and duration will

be used as a template for commissioning and decommissioning. A multi-disciplinary assessment centre team will be created that will comprise of:

- Assessment Centre Administrator (1x)
- Operational staff (2x)
- IT staff (1x)
- Commercial removal staff

1.7.5.3 Equipment supply and maintenance

An assessment centre equipment specification will be constructed based on knowledge and experience gained from the integrated pilot. A “working set” of equipment will be procured for each of the centres running in parallel. These equipment sets will be inventoried, and preventative maintenance, routine servicing and calibration managed by the Centre Administrator. Hardware obsolescence after two years has been planned and budgeted.

Equipment failures will be immediately reported by the assessment centre nursing manager to the Centre Administrator in the coordinating centre. UK Biobank will hold an appropriate level of back-up equipment which can be dispatched by courier in the event of equipment failure that affects participant processing. Repair and/or replacement of defective equipment will be managed by the Centre Administrator.

1.7.5.4 Consumables supply

Supply of consumables required by each operating assessment centre will be managed by the Centre Administrator in the coordinating centre. A monthly/bi-monthly standing order delivery to each centre will be established in line with projected participant recruitment. There will be a small buffer stock held in each centre to compensate for greater-than-projected demand. A larger stock will be held in the coordinating centre so that supplies can be dispatched to an assessment centre by courier in the event of a unexpected problem arising (e.g. a damaged batch of ACD tubes).

1.7.5.5 Health & safety

The Centre Administrator will sit on UK Biobank’s health and safety committee and will ensure that each operational assessment centre has the required health and safety documentation. Any potentially harmful substances will be controlled using the COSHH policy and procedures. The Centre Administrator will ensure that relevant SOPs are current and comply with health and safety legislation, and will liaise closely with the Training Coordinator and Monitor to ensure compliance.

2 DEVELOPMENT OF THE RESOURCE

2.1 Overall strategy

UK Biobank aims to recruit 500,000 people from all around the UK who are currently aged 40-69, and then to follow their health long-term through medical and other health-related records. Recruitment will be via centrally coordinated identification and invitation from population-based registers (such as those held by the NHS) of potentially eligible people living within a reasonable travelling distance of an assessment centre (see Section 2.2). This central recruitment strategy will allow invitations to be targeted to enhance generalisability and to make allowance for the impact on participation rates of various factors (e.g. age, sex, ethnicity, socioeconomic status). Each assessment centre will aim to recruit as many as possible of the nearby target population during a period of about six months to one year (depending on the local population density and transport links), and will then be relocated in order to achieve recruitment across most of the UK.

When an individual arrives at the assessment they will be asked for their consent to participate, and will then move through a series of assessment stations involving questionnaires, measurements and blood/urine sampling (see Section 2.3). This baseline assessment visit takes an average of about 90 minutes, with about 14 staff required to process over 100 people daily. Staff with an appropriate mix of nursing and technical experience will be recruited and trained specifically for UK Biobank. A fully integrated clinic IT system has been developed specifically for the assessment centre visit, with each designated station having a desk top computer linked via a secure local area network to the main assessment centre server. At the end of each day, participant data and samples will be transferred securely to the UK Biobank coordinating centre (see Sections 2.4 & 2.7). Following sample processing in the central laboratory, multiple aliquots will be stored in an automated -80°C working archive and, at a geographically distinct location, in a back-up liquid nitrogen store for security.

It is anticipated that follow-up will be via both the primary care record (which includes all primary care generated entries and directly linked entries, such as laboratory tests requested by GPs) and the national care record (which will include summary entries from primary, secondary, tertiary and community care, including Hospital Episodic Statistics [HES]). UK Biobank is also in discussion with the Secondary Uses Service (SUS) with a view to obtaining data on death certification and cancer registration (as an alternative to the Office for National Statistics). With the rapid pace of change that is currently occurring in the implementation of NHS electronic records (particularly in primary care), it is intended that detailed planning for participant follow-up only commence after recruitment is well established (see Section 2.6). Such deferral has the advantage that both the quality and quantity of available data will increase over the next few years, and the systems currently under development will be more fully deployed.

2.2 Identification and invitation

2.2.1 General approach

The general approach to the identification and recruitment of participants is summarised in figure 2.2.1, and has been informed by experience from the integrated pilot involving around 4000 participants.

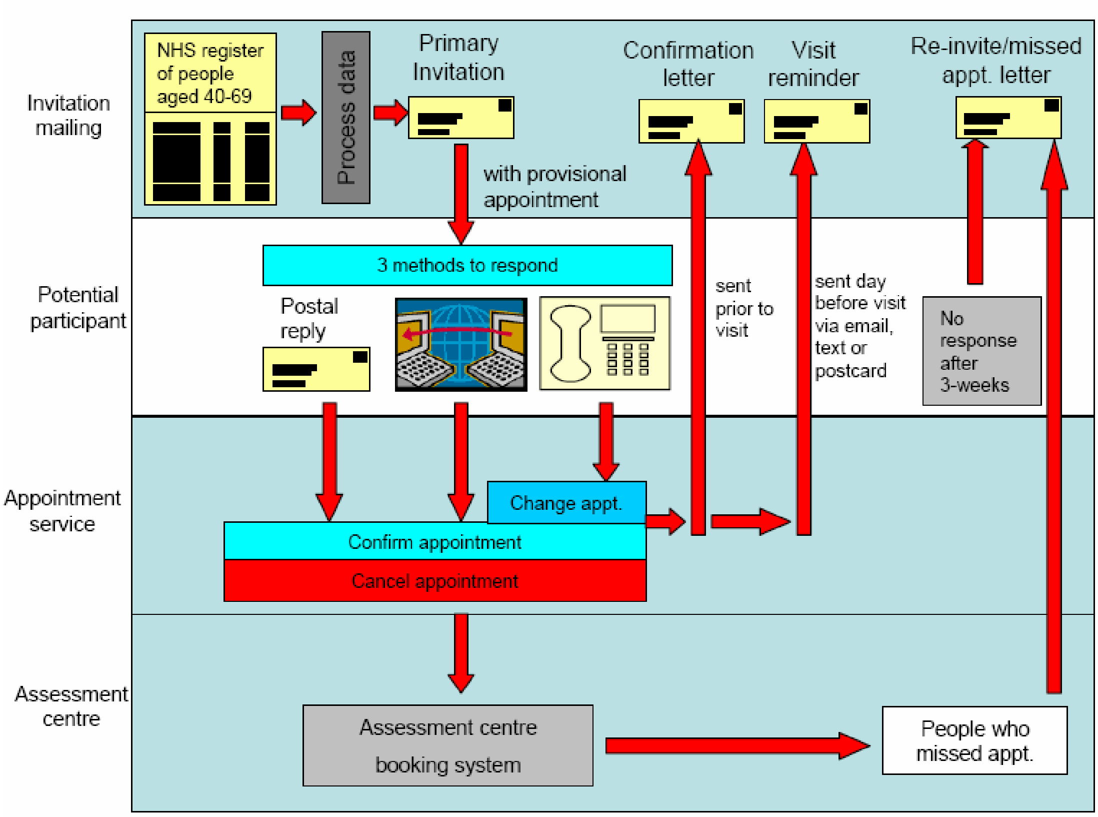

Figure 2.2.1: Schematic of invitation and appointment system

2.2.2 Identification of potential participants

In the United Kingdom, virtually all members of the general population are registered with a general practitioner through the National Health Service. Assessment centres will be located in accessible and convenient locations with a large surrounding population, and people to invite will be identified from NHS patient registers according to being aged 40-69 and living within a reasonable travelling distance of an assessment centre. Based on previous experience in the integrated pilot phase, it is estimated that about 5 million primary invitations may need to be mailed in order to recruit 500,000 participants.

2.2.2.1 Provision of NHS registry data

Following discussions with the Department of Health (specifically the DoH Caldicott Guardian and the Patient Information Advisory Group), it is intended that access to NHS patient registers will be obtained from a few national sources. This will avoid the delays in invitation mailing experienced in the integrated pilot phase as a result of the need to gain separate access through each Primary Care Trust (PCT) that manages individual patient registers. Data transfer and subsequent processing for invitation mailing will be covered by an agreement between the Department of Health (as data controller) and UK Biobank (as the data processor) in compliance with the Data Protection Act. It will be limited to the following information on people aged 40-69:

- title; forename; surname;
- gender;
- address;
- date of birth;
- name and address of General Practitioner (GP);
- NHS number

UK Biobank will receive no confidential medical information on potential participants. Date of birth and the NHS number are required to verify age and for the purposes of duplicate removal respectively. GP contact details will be used to inform them that people registered with their practices are being invited to participate (see Section 2.2.5.4).

2.2.2.2 Processing of contact details

As necessary, UK Biobank will process these NHS register data to remove duplicate records and to check that the person is aged 40-69 from their date of birth, and to remove the records of people who have died by screening against death certificate registration (e.g. Office for National Statistics). Postal addresses will be enhanced using commercially available software. In order to recruit a widely generalisable population, the invitation mailing will be stratified according to key demographic parameters (including age, gender and postcode areas as a measure of social deprivation), with over-sampling of particular groups as required. A provisional assessment visit appointment will then be generated for each potential participant.

2.2.3 Invitation mailing to attend assessment centre

A commercial mailing house will be contracted to UK Biobank to undertake the invitation mailing. The contract will ensure that the data received can only be used for the purpose of invitation mailing to participate in UK Biobank (in accordance with the Data Protection Act). The mailing house will be sent the following information for the purpose of invitation mailing:

- title; forename, surname;
- address;
- time, date and location of provisional appointment;

- unique mailing identifier number

2.2.3.1 Invitation letter with provisional appointment

Potential participants will generally be sent an invitation letter at least 6-8 weeks ahead of the date of their provisional appointment. The initial invitation mailing will include the following:

- invitation letter (with notes about confirming appointment on the back);
- participant information leaflet;
- pre-paid postal reply form.

The invitation letter will provide a pre-booked *provisional* appointment at the assessment centre. Potential participants will be asked to confirm their appointment within two weeks of receiving the invitation letter by:

- Telephoning the freephone service: if the appointment on the invitation letter is not convenient then it can be changed during this call; or

- Mailing the reply form in the pre-paid envelope provided or visiting the study website: this allows the appointment in the invitation letter to be confirmed (but not changed).

People who do not want to take part are asked to indicate this on the reply form, on the study website, or by telephone (although this is optional) so that the appointment can be re-allocated. In such cases, information will be sought about the main reason(s) for non-participation.

2.2.3.2 Information for participants

The participant information leaflet included in the invitation mailing will provide detailed information about UK Biobank. It also indicates that further information is available via the Freephone service or the study website. In addition to the opportunity to discuss the study with a member of the team via the Freephone service, a further information leaflet is available for potential participants.

2.2.3.3 Confirmation of appointment

People who confirm an assessment centre appointment will be sent a written confirmation of their appointment, along with advice on preparations for attending the assessment centre. This confirmation mailing will include the following:

- Confirmation letter (with the pre-visit questionnaire on the back);
- Directions for attending the assessment centre (including a map showing parking and bus/train stops).

2.2.3.4 Pre-visit questionnaire

The pre-visit questionnaire provides participants with an opportunity, ahead of their assessment visit, to record information that they might have difficulty recalling during the visit (e.g. medications, operations, family medical history and birth details). Such details will be entered directly into the assessment centre computer during the visit, and these pre-visit aide memoires will not be retained.

2.2.4 Freephone appointment and information service

The Freephone service will be operational on Monday to Saturday from 8am to 7pm. It will be staffed by specially trained staff (based at the Welsh Regional Collaborating Consortium in Cardiff) using an integrated computer system developed and piloted specifically for the purpose of appointment booking in UK Biobank. The main functions of the recruitment service are summarised below:

- To confirm or change a pre-booked appointment (and, with verbal consent, to record telephone/mobile phone/e-mail details in case the appointment must be cancelled or changed at short notice, and to send a reminder just before the appointment);

- To cancel the invitation and ensure the invitee receives no further contact (and, with verbal consent, to seek the main reason(s) for not participating);

- To allow questions from potential participants (and their GPs) to be addressed either by the trained call centre staff or, if not possible, by more senior members of the UK Biobank team;

Based on experience during the integrated pilot phase, a question and answer manual has been developed and integrated into the computer system (as well as being available on the UK Biobank website). This provides the call centre staff with standard answers to the most common questions (e.g. transport and parking; travel expenses; assessment centre procedures; consent and withdrawal; feedback of results; confidentiality) and allows the questions asked by potential participants to be logged. The call centre staff will also be responsible for processing the postal replies to invitations.

2.2.5 Other mailings and reminders

2.2.5.1 Re-invitation letter

About 3 weeks after mailing the invitation letter, people who have not responded may be sent a re-invitation letter once only (although, since experience in the integrated pilot suggested that such mailings may not be cost-effective, their value will be continually assessed). This letter will advise them that, if they might still be interested in attending an assessment visit, they need to contact the freephone service in order to book an appointment (as their previous appointment may have been re-allocated). It will also

indicate that further copies of the participant information leaflet can be obtained from the freephone service or from the UK Biobank website.

2.2.5.2 Pre-visit reminder message

When potential participants confirm their appointment by telephone or by mail, they will be asked to provide an e-mail address and/or mobile phone number. (Based on responses in the integrated pilot, more than 50% of participants are likely to have access to e-mail.) With the participant's agreement, these details will be used to send a reminder, via e-mail or SMS-text to a mobile phone, just before their scheduled appointment with a message along the following lines:

"A reminder of your UK Biobank appointment at [TIME] on [DATE].
If you have any questions, please call Freefone 0800-0-276-276."

Alternatively, for those people who do not have such contact details, a similar reminder may be mailed to them a few days before their appointment.

2.2.5.3 Missed appointment letter

Potential participants who confirm an assessment visit appointment but then do not attend will be sent a letter within 1-2 weeks of the missed appointment. This will ask them to contact the freephone service to book another appointment if they might still like to participate. (N.B. In the integrated pilot phase about 10-20% of participants did not attend their confirmed appointments, but the use of pre-visit reminders approximately halved this rate of non-attendance.)

2.2.5.4 General practitioner (GP) letter

UK Biobank's invitation mailing system will automatically generate letters to GPs, just prior to the first person being invited from the particular practice, informing them that their patients are about to be invited to participate in UK Biobank. This letter will be accompanied by several copies of the participant information leaflet, which the GP will be asked to share with colleagues in their practice. It will also indicate that further information about UK Biobank is available via the freephone service or dedicated website.

2.2.6 Increasing local awareness of UK Biobank

In parallel with the central processes of identifying and inviting eligible participants to the assessment centre, a number of activities will take place aimed at raising awareness of UK Biobank to improve the local response rates. A communications expert based in the coordinating centre will liaise with existing communications experts based locally either within the organisations representing the different RCCs or, where necessary, freelance individuals. The aim will be to plan, and implement, a number of public relations activities that raise and maintain local awareness of UK Biobank, and its aims, which are adapted to local circumstances. This might involve

features in local press and radio, including interviews with members of the relevant RCC, local participants and celebrities championing the resource. In addition, there may be engagement with stakeholder groups that might either be affected by UK Biobank or have a particular interest in its outcome (such as general practitioners and local practice staff who may have patients asking about it). Activity aimed at these groups could be either at the local level (e.g. through GP research networks) or more broadly through professional journals (such as "The Generalist" for GPs). Opportunities will also be explored for joint promotion with regional and local branches of medical research charities (such as the British Heart Foundation or Cancer Research UK) that support the aims of UK Biobank.

2.2.7 Information to be retained on non-participants

After the end of the recruitment phase, *anonymised* information only will be retained on all non-participants (i.e. did not respond or declined to participate) to allow the sampling frame to be defined with respect to: sex; month and year of birth; and Super Output Area (SOA). Post-codes for home addresses will be converted to lower layer SOAs (www.statistics.gov.uk/geography/soa.asp), which cover a minimum population of 1000 people (mean 1500) and provide information about socioeconomic class. Lower layer SOAs are built from groups of Output Areas (typically 4 to 6) and constrained by the boundaries of the Standard Table wards used for the 2001 Census. Upon conversion to SOAs, post-codes for non-participants will be safely and securely destroyed.

This information will allow issues about participation rates among different groups to be addressed, and help determine extra measures to recruit hard-to-reach groups (including the location of assessment centres and other targeted recruitment strategies). Subsequently, comparisons in terms of various demographic factors (such as age, gender, urban/rural, socioeconomic class) may be of relevance for considering the generalisability of the recruited cohort.

2.3 Baseline assessment

2.3.1 Assessment centre specification and staffing

Assessment centres are to be conveniently located with good public transport links, proximity to parking, and disabled and out-of-hours access. They are likely to be established either in commercial office space (as in the integrated pilot) or in academic clinical research facilities (see Section 1.7). Experience from the integrated pilot indicates that a total floor space of up to 2200 square feet is required. As in the integrated pilot, visit stations will be constructed using a combination of the space available and dedicated partitioning for privacy (Figure 2.3.1). The centre will ideally have dedicated toilets for urine sampling and infrastructure to connect the assessment centre computers via a secure network. Assessment centres are expected to be operational for an average of about 6 months before being relocated, and it is intended that 5-8 will be operational at any one time.

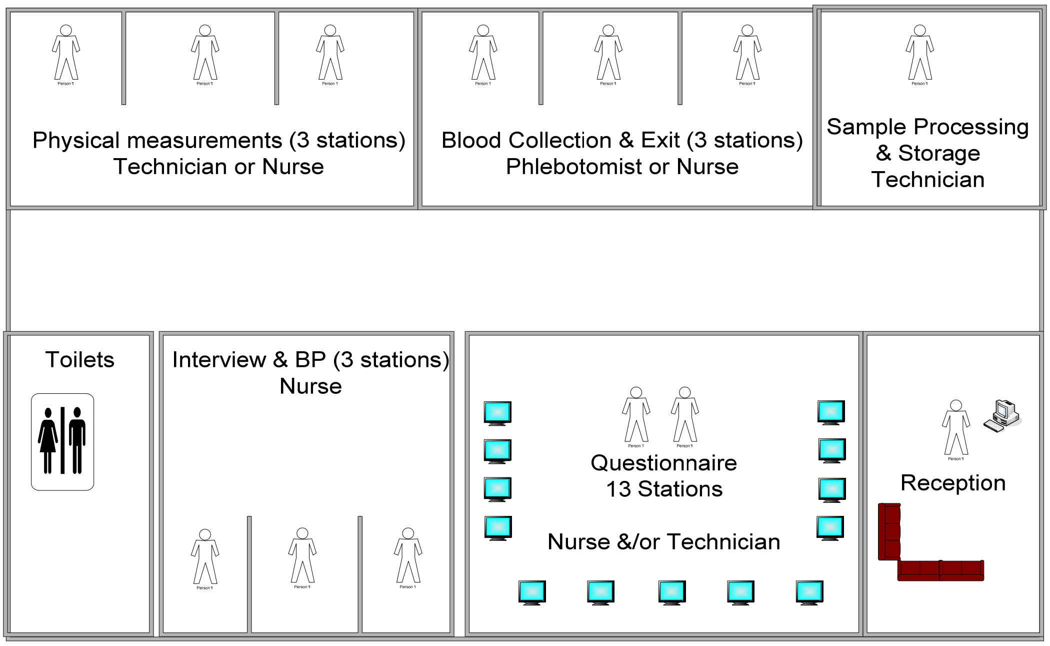

Figure 2.3.1: Notional layout of assessment centre stations

Appointment scheduling by the coordinating centre will be managed in order that each assessment centre assesses more than 100 participants per day from Monday to Friday, and more than 80 on Saturday. Based on the integrated pilot experience, this is likely to require 13-14 staff to be on duty from a pool of around 20-25 trained staff. The usual opening hours will typically be Monday to Friday 8.00 am to 8.00 pm (last appointment starting at 7.00 pm) and Saturday 8.00 am to 6.00 pm (last appointment starting at 5.00 pm). Staffing will involve a cost-effective combination of nurses, healthcare technicians and receptionists, recruited chiefly from nursing agencies. Senior nurses will be appointed as the centre managers reporting to UK Biobank's Clinical Operations Manager (see Section 1.7.5).

2.3.2 Training and monitoring

Prior to being appointed, all assessment centre staff will undergo a formal interview to assess their suitability and relevant experience (e.g. nursing or phlebotomy staff must be experienced at venepuncture). An up-to-date copy of the curriculum vitae of each staff member will be maintained in the coordinating centre and the relevant assessment centre, along with their training record specifying the procedures they are approved to undertake. The assessment centre IT system will only allow staff (via username and password protection) to perform approved procedures.

2.3.2.1 Core training programme

The core training programme for all assessment centre staff will be undertaken over a period of 3-5 days (although not all staff will be required to attend each day) during the week prior to the assessment centre opening. Training will be organised by UK Biobank's Clinical Operations Manager in the newly commissioned assessment centre, with individual modules delivered by specialised trainers (see Box 2.3.1).

Sessions	Areas covered	Staff trained
1. Introduction	• Overview of UK purpose, assessment visit & IT system;<br>• Consent process;<br>• Participant welfare.	All staff
2. Questionnaire	• Background to touch-screen and interview questionnaires;<br>• Use of touch-screen;<br>• Administration of interview.	Nurses (who do interviews) & all staff supervising touch-screens
3. Physical measurements	• Introduction to measurements (including rationale and need for standard technique to produce high quality data);<br>• Maintenance and calibration of equipment;<br>• Workshop using all equipment.	Nurses and technicians doing measurements
4. Biological sample collection & processing	• Health & safety, and participant welfare;<br>• Venepuncture technique;<br>• Urine collection;<br>• Sample processing;<br>• Courier transfer process.	Nurses and phlebotomists collecting blood and urine (& processing)
5. Practice sessions	• Q&A session with senior members of team;<br>• Practice runs of baseline assessment visit.	All staff

Box 2.3.1: Training program for assessment centre staff

New staff joining after the assessment centre is operational will receive the specific training that they require from the Clinical Operations Manager and experienced staff working in the assessment centre. When a centre closes, some staff may be able to transfer to a nearby one when it opens (for example, in some major cities), and the roll out plan aims to facilitate this continuity of expertise (see Section 1.7). These experienced staff will not be required to repeat the core training, but will instead be used to support the training and mentoring of new staff.

2.3.2.2 Mentoring and monitoring

Following initial training, assessment centre staff will receive a period (length dependent upon experience) of mentoring during which they will be observed by experienced members of the assessment centre team (and, if required, external trainers) while undertaking routine assessment procedures (with verbal consent from participants). Mentoring will also be undertaken on an ongoing basis by the Clinical Operations Manager and other members of the coordinating centre team.

Assessment centre monitoring will be undertaken by the coordinating centre, in consultation with collaborators in the related RCC, using a combination of computer review of assessment centre data and periodic monitoring visits. The computerised data review will focus on the following aspects at each assessment centre:

- ***Participants assessed per day:*** This will be compared with the number of confirmed appointments to highlight any potential issues with the operation of the assessment centre;

- ***Visit timings:*** The average and range of times taken for individual stations in the assessment visit (overall and for each staff member), and the times between stations, will be used to identify any issues with participant throughput (e.g. bottlenecks leading to long waiting times);

- ***Missing data:*** The number of participants missing one or more stations (and the reasons recorded) and, for blood or urine, the number of samples missing or unfit for processing at the coordinating centre will be used to identify process failures;

- ***Data quality:*** The number of outliers for each physical measurement and, for measurements with more than one value (blood pressure and spirometry), excessive variability within participants will be used to help identify staff requiring additional mentoring.

Any issues identified during this continuous review of the assessment centre data will be followed up by a monitoring visit undertaken by UK Biobank's Clinical Operations Manager or Clinical Research Associate (or other relevant member of the coordinating team). In addition, regular monitoring visits will be undertaken to each assessment centre by the coordinating centre team and collaborators from the RCCs.

2.3.3 Assessment Centre Environment (ACE) IT system

2.3.3.1 System architecture

A fully integrated IT system that includes all of the hardware and software applications required to allow direct electronic data capture has been developed and evaluated in the integrated pilot study. As required, data are input to desktop computers via keyboards, touch-screens, bar-code readers and direct transfer from measurement devices (for example, the electronic sphygmomanometer or spirometer). These computers are connected via secure wireless or Ethernet local area networks through a local server to the remote core Biobank databases. Person-identifiable information is not kept at the local assessment centre for longer than necessary.

2.3.3.2 Data transfer between visit stations

Each participant will be issued with a Universal Serial Bus (USB) memory key at the start of the visit. This memory key acts both as the participant identifier (i.e. contains ID, name, date of birth and gender) and as a back-up temporary storage device for data recorded during the visit. At the end of the visit, all data on the key will be removed following successful back-up to the assessment centre local server and/or central core databases.

2.3.4 Assessment visit sequence and timing

Based on the integrated pilot experience, the baseline assessment visit is expected to take around 90 minutes. It involves the participant moving through a fixed sequence of visit stations, with the sequence and expected timing of each station shown in Box 2.3.2.

Visit Station	Assessments undertaken	Estimated time (mins)
Reception	• welcome & registration<br>• consent	10
Questionnaire	• touch-screen questionnaire<br>• cognitive function tests	40
Interview (& blood pressure)	• interviewer questionnaire<br>• blood pressure measurement	10
Physical measurements	• height (both standing & sitting)<br>• hip & waist measurement<br>• bio-impedance measurement<br>• hand-grip strength<br>• *heel bone ultrasound*<br>• spirometry	15
Sample collection (& exit)	• blood sample collected<br>• urine sample sought<br>• consent & result summary printed<br>• travel expense claim provided	15
TOTAL		90

Box 2.3.2: Estimated time for assessment visit

2.3.5 Baseline assessment procedures

This section provides a summary of the visit procedures (with full details provided in the assessment visit Standard Operating Procedures).

2.3.5.1 Reception station

The reception station will be staffed by two receptionists (clerical grade), and equipped with a reception desk and seating for several people (although the appointment scheduling aims to minimise any waiting time at reception). The following activities will be undertaken at the station:

- Attendees will be welcomed and asked if they have their appointment confirmation letter so that their unique ID can be scanned with a bar-code reader. If the person does not have the letter, their details can be recalled from their appointment time, name, and address;

- Name, address and date of birth will be verified, and attendance confirmed by the receptionist in the appointment booking system;

- Potential participants will be asked if they have read the Participant Information Leaflet that was sent to them and, if not, offered a copy. More detailed written information can be provided with the Further Information Leaflet;

- A small USB memory key will be given to the participant, which will be used for registration at each of the visit stations and temporary storage of

all data during the visit (and then uploaded to the centre server at the exit station and deleted from the key);

- Participants will be advised that water is available at all times during the visit and that a urine sample will be sought at the end of the visit (with tea/coffee available at the end of the visit after blood sampling).

Following completion of these procedures, the receptionist will seat the person at one of the touch-screen stations and hand over to the staff member (nurse or healthcare technician) responsible for these stations.

2.3.5.2 Consent station

At least one member of staff will be available to introduce the participant to the touch-screen system and to answer any questions about UK Biobank. The room will be sufficiently large to accommodate 10-12 touch-screen computers and to provide each participant with privacy by spacing and partitions. The staff member will take the person through the consent process:

- The participant's USB memory key will be connected, the staff member will enter their username and password, and will then confirm the identity of the person before introducing the use of the touch-screen;

- The potential participant will be asked to confirm on the touch-screen that they are ready to begin the consent process, and summary information about UK Biobank will then be displayed;

- The potential participant will be asked to select the "I agree" button for each of the individual statements on the Consent Form and, only if all of these statements are selected, asked to provide their signature on an electronic pad;

- If the participant selects "I disagree" for any of the consent questions, a message will be displayed to contact a member of staff who will then provide further information and clarification on any issues. (N.B. More senior staff will also be available and, should it be required, senior members of the central UK Biobank team can be contacted by telephone at any time during assessment centre operation.)

The computer system will not allow any subsequent stations to be undertaken unless the consent process has been completed by the participant signing the consent form and a member of staff verifies that this has been done.

2.3.5.3 Touch-screen questionnaire station

When the participant has completed the consent process, they will remain seated at the station and the supervising member of staff will introduce the touch-screen questionnaire:

- The participant will be advised to aim to spend about 30 minutes on the questionnaire (and shown the indicators of elapsed time and amount competed), not to dwell for too long on any questions, and to skip any questions that they do not wish to answer (e.g. considered sensitive);

- In the unlikely event that a participant is unable to complete the questionnaire using the touch-screen, the staff member will initiate the keyboard and mouse;

- Periodically during the touch-screen questionnaire, the staff member will check on the progress of the participant to ensure they are not experiencing any difficulties completing the questionnaire;

Following completion of the touch-screen questionnaire, the staff member will sign it off, return the USB memory key to the participant and direct them to the interview and blood pressure station.

2.3.5.4 Interview and blood pressure station

There will be three separate interview and blood pressure stations to avoid a bottleneck, and each station will be manned by a nurse and partitioned to provide sufficient privacy for the interview and procedure. The participant will be seated at one of the stations and the following activities undertaken:

- Their USB memory key will be connected, the staff member will enter their username and password, and will then confirm the identity of the person before introducing the interviewer questionnaire;

- After completing the interview with the participant (which ensures that they have been seated for at least 5 minutes), blood pressure and pulse will be measured twice (with a minimum interval of one minute) using an Omron 705 IT monitor connected directly to the computer;

- During the rest period between measurements, the staff member can enter information recorded by the participant on the pre-visit questionnaire (which will not be retained);

Following completion of the station, the staff member will sign it off, return the USB memory key to the participant and direct them to the physical measures station.

2.3.5.5 Physical measures station

There will be three separate physical measures stations to avoid a bottleneck, and each station will be manned by a healthcare technician (or nurse), and partitioned to provide sufficient privacy for the procedures. The participant will be seated at one of the stations, asked to remove their shoes and socks, and the following activities undertaken:

- Their USB memory key will be connected, the staff member will enter their username and password, and will confirm the identity of the person before introducing the measurements to be undertaken;

- The correct procedure for assessing grip strength will be demonstrated before the participant is asked to provide a single measure of hand grip strength for each hand using a Jamar Hydraulic hand dynamometer, with the results typed into the computer;

- The circumference of both waist (at the position of the natural indent) and hip (at the widest point) will be measured using a Seca-200 tape measure (without the participant being required to remove any clothes), with the results typed into the computer;

- Standing and sitting (using a custom made seat) height using the Seca 202 height measure with both results typed into the computer;

- Before measurement of body impedance, the staff member will check that the participant does not have a pacemaker or is pregnant (requiring measurement of weight using manual scales). If such contraindications are not present, the participant will be asked to stand in their bare feet on the measuring plate of the Tanita BC418ma bio-impedance device, and to firmly hold the handles with their arms hanging loosely at their sides. A single measure of weight, impedance and estimated percent fat will be recorded directly into the computer;

- A single measure of calcaneal bone density will be undertaken on the left heel using a Norland McCue Contact Ultrasound Bone Analyser (CUBA) with the participant sitting upright. The measurement takes 1-2 minutes and, during this time, the participant will be asked to watch a short video demon-demonstrating the correct procedure of spirometry (see below). Results for the Broadband Ultrasound Attenuation (BUA) will be recorded on the computer;

- The staff member will check that the participant does not have any contraindications to spirometry (e.g. recent chest infection or heart attack; recent chest, abdominal or eye surgery; history of detached retina or pneumothorax: any reported will be recorded and spirometry not undertaken). If such contraindications are not present, it will be explained that the aim is to record two acceptable blows (defined as differences between the blows of less than 5% in both FVC and FEV_1) from a maximum of three blows (with the computer automatically analysing the blows and indicating whether a third blow is required);

- The participant will be given the Vitalograph pneumotrac spirometer fitted with a new disposable mouthpiece/spirette and asked to sit with their back straight and feet firmly on the floor. They will be instructed to fill their lungs as much as possible, ensure their lips are sealed around the mouthpiece (without blocking it with teeth or tongue), and then to blow out as hard and as fast as possible (ideally for at least 6 seconds). During the procedure,

the staff member will encourage the participant to continue blowing until no more air will come out;

- The flow curves will be recorded directly into the computer and the staff member will, if necessary, show the curve to the participant in order to highlight any issues which could be improved on subsequent blows;

Following completion of the station, the participant will be asked to put on their shoes and socks, and the staff member will sign it off, return the USB memory key to the participant and direct them to the sample collection (and exit) station.

2.3.5.6 Sample collection (and exit) station

There will be two separate sample collection stations to avoid a bottleneck and each station will be manned by a phlebotomist (or nurse), and partitioned to provide sufficient privacy for blood collection. The participant will be seated at one of the stations and blood collection undertaken:

- Their USB memory key will be connected, the staff member will enter their username and password, and confirm the identity of the person before introducing the procedure;

- The computer will generate a printed copy of the participant's consent form and a report on the key measurements from their assessment visit, which they can review while blood is being collected. If there are any questions about values on the report, the participant will be advised to contact their GP or NHS-Direct.

- The phlebotomist will check whether the participant has had any previous problems giving blood and will then inspect the suitability of the veins in the inner elbow region. If these veins appear suitable then blood collection will be undertaken from the inner elbow using an 18G green vacutainer needle and barrel;

- Should the veins in the inner elbow appear unsuitable or blood collection fails on a previous attempt from this region then permission from the participant will be sought to attempt blood collection from veins on the back of the hand using a 21G Safety Lok butterfly needle connected to a vacutainer barrel;

- Alcohol wipes will only be used to clean the area of skin for blood collection if the skin is visibly dirty (and, if wipes are used, 30 seconds will be allowed to elapse for evaporation of alcohol before inserting the needle to prevent sample contamination or pain for the participant);

- Bar-coded vacutainer tubes will be used to collect blood in the order (based on priority) shown in the box below, using pre-prepared racks;

Order of collection	Vacutainer tube	Preservative	Cap colour	Volume
1	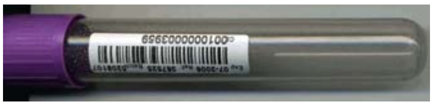	EDTA	Purple	9 ml
2	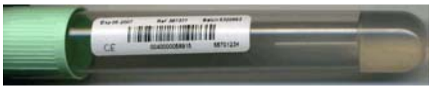	EDTA (plasma separator)	Green	8 ml
3	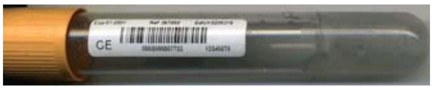	Clot activator (serum separator)	Orange	8 ml
4	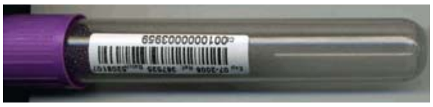	EDTA	Purple	9 ml
5	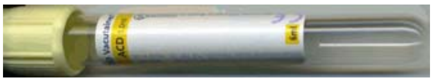	Acid citrate dextrose	Pale yellow	6 ml
6	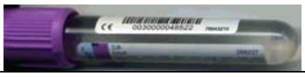	EDTA	Purple	4 ml

- Immediately following collection, all vacutainers containing blood will be scanned with the bar-code reader (part-filled tubes will be scanned, but not any unfilled tubes) and transferred immediately to the sample processing area. (Scanning activates a timer on the sample handling computer to advise the relevant staff member to collect the tubes and to allow the clot activator tube to stand at room temperature for 30 minutes prior to centrifugation: see Section 2.4.);

Following blood collection, the staff member will verify from the computer that the participant has completed all of the stations (and, if not, that a reason has been recorded for missing any station or arrange for that station to be completed).

The participant will then be asked if they are able to provide a urine sample, and, if so, provided with a urine collection pot and bar-coded vacutainer (scanned to assign the bar-code to the participant) in an opaque plastic bag, directed to the toilet and asked to return the sample to the collection box outside the station.

Finally, the participant will be thanked and asked if they wish to claim travel expenses; if they do, then they will be given a claim form to complete and return by mail subsequently (or leave with the receptionist).

2.3.6 Post-visit questionnaire

Within 4 weeks of attending the assessment visit, a random sample of participants will be sent a post-visit questionnaire to complete anonymously and return in a pre-paid envelope (as in the integrated pilot phase). This questionnaire aims to assess participants' understanding of the project and their consent, as well seeking opinions on the assessment visit and highlighting areas for improvement. It is anticipated that the questionnaire will be sent to a random sample of about 5-10% during the first few weeks of operation of any new assessment centre and then subsequently as needed.

2.4 Sample processing

2.4.1 Processing of blood and urine at the assessment centre

Processing of blood and urine samples at the assessment centres will be minimal in keeping with the UK Biobank sample handling pilots [105]. As blood is collected from a participant, the vacutainers are to be inverted ten times to mix the anticoagulant/preservative/clot activator with the whole blood. After collection of a complete set of vacutainers, the unique bar-code on each one will be scanned into the assessment centre IT system that links each vacutainer with the unique participant identifier number. This is important to link the participant data from the assessment centre with the start of the laboratory data structure in the central Laboratory Information Management System (LIMS). It will also automatically initiate a timer built into the assessment centre IT system to allow accurate measurement of clotting time for the serum separator tube.

The blood in the plasma separation tube is to be immediately centrifuged at 2500g for 10 minutes and the time of centrifugation recorded in the assessment centre IT system. The blood in the serum separator tube will be allowed to clot for 25-30 minutes at room temperature before centrifugation at 2500g for 10 minutes; the time of centrifugation is to be recorded in the assessment centre IT system. Urine from the urine collection vessel will be transferred to the pre-assigned bar-coded vacutainer by removing the protective label from the lid of the collection vessel and pushing the cap of the vacutainer onto the sheathed needle in the vessel recess. All vacutainers are to be maintained at 4^oC (with the exception of the acid citrate dextrose tube which is to be maintained at 18^oC) until ready for packing and dispatch to the coordinating centre laboratory in temperature-controlled shipping boxes. The boxes will be collected by a commercial courier and transported overnight to the central laboratory where they will be processed and transferred to ultra-low temperature archives..

2.4.2 Processing of blood and urine at the central processing laboratory

When the vacutainers arrive at the central laboratory, they will be processed as soon as possible according to Table 2.4.1. All of the vacutainers that arrive will be scanned and compared against the LIMS data file from the assessment centres to ensure the correct tubes have arrived and the

laboratory data file can be linked to the other participant data. The vacutainers will then be processed using automated systems (see below), with times and temperatures of all operations and operator identifiers logged in the LIMS.

Vacutainer tube	Fractions	Number of aliquots	
		-80°C	Liquid N_2
EDTA (9ml) x 2	Plasma	6	2
	Buffy coat	2	2
	Red cells	-	2
EDTA (PST)	Plasma	3	1
Clot activator (SST)	Serum	3	1
ACD	DMSO blood	-	2
EDTA (4 ml)	Haematology (immediate)	-	-
Urine	Urine	4	2
TOTAL ALIQUOTS		18	12

Table 2.4.1: Fractions and aliquots of blood and urine samples

2.4.2.1 EDTA (9 ml) vacutainers

The large EDTA vacutainers will be transferred to the laboratory's automated blood fractionation system for processing. Blood fractions will be separated by automated centrifugation at 2500g for 10 minutes at 4°C. Following digital imaging, each vacutainer will be transferred to liquid handling robots that aliquot the blood fractions at 4°C into 2D bar-code labelled 1.4ml cryostorage tubes with split septum seals arrayed in 96 position racks (designed to the Society for Biomolecular Screening standard footprint: Figures 2.4.1a & b). The digital image and associated software are used to define the interfaces of the various fractions which are then associated with the unique bar-code on the vacutainer by the liquid handling robots. Four aliquots of plasma (about 800ul each), 2 aliquots of buffy coat (about 200ul each) and 1 aliquot of red cells (about 1ml) will be taken from each vacutainer according to Table 2.4.1 for long-term cryopreservation. The bar-codes on the 1.4ml sample storage tubes will be attributed to the bar-code on the vacutainer and the LIMS data set updated.

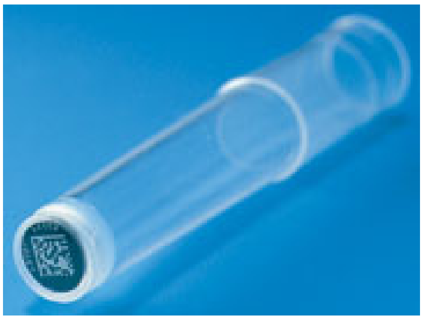

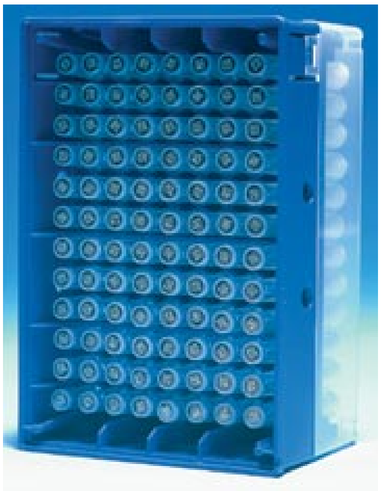

Figure 2.4.1a: 2D bar-code labelled 1.4ml aliquot storage tube (without seal); and Figure 2.4.1b: 96 x 1.4ml tubes in SBS footprint storage rack

2.4.2.2 EDTA Plasma Separator Tube (PST) vacutainers

The PST vacutainers will be transferred to the automated blood fractionation system for processing. Following digital imaging, each vacutainer will be transferred to liquid handling robots that aliquot the plasma fraction at 4°C into 2D bar-code labelled 1.4ml cryostorage tubes with split septum seals arrayed in 96 position racks (Figures 2.4.1a & b). Four aliquots of plasma (about 800µl each) will be transferred from each vacutainer according to Table 2.4.1 for long-term cryopreservation. The bar-codes on the 1.4ml sample storage tubes will be attributed to the bar-code on the vacutainer and the LIMS data set updated.

2.4.2.3 Clot activator Serum Separator Tube (SST) vacutainers

The SST vacutainers will be transferred to the automated blood fractionation system for processing. Following digital imaging, each vacutainer will be transferred to liquid handling robots that aliquot the serum fraction at 4°C into 2D bar-code labelled 1.4ml cryostorage tubes with split septum seals arrayed in 96 position racks (Figures 2.4.1a & b). Four aliquots of serum (about 800µl each) will be transferred from each vacutainer according to Table 2.4.1 for long-term cryopreservation. The bar-codes on the 1.4ml sample storage tubes will be attributed to the bar-code on the vacutainer and the LIMS data set updated.

2.4.2.4 Acid citrate dextrose (ACD) vacutainers

The ACD vacutainers will be transferred to a customised TECAN liquid handling platform configured inside a laminar airflow cabinet maintained at 18°C. Two 500µl aliquots of whole blood from each tube will be mixed with two 500µl aliquots of sterile 20% DMSO (diluted in RPMI growth medium) in 2D bar-coded 1.4ml sample storage tubes with split septum seals arrayed in 96 position racks (Figures 2.4.1a & b). These storage tubes will then be transferred to a -80°C environment in insulated polystyrene containers for 16 hours prior to long-term cryopreservation in the liquid nitrogen back-up store

(see Table 2.4.1). The bar-codes on the 1.4ml sample storage tubes will be attributed to the bar-code on the vacutainer and the LIMS data set updated.

2.4.2.5 EDTA (4 ml) vacutainers

The small EDTA vacutainers will be transferred directly into assay cassettes that hold 10 tubes and oriented so that the bar-codes are readable. Whole blood is used for a standard range of haematological parameters (Box 2.4.1) on a Beckman automated haematology analyser. Data will be attributed to the vacutainer bar-code and the LIMS data set updated.

Haemoglobin	Platelet Count
Packed Cell Volume	White Cell Count
Red Cell Count	Neutrophil count
Mean Cell Volume	Lymphocyte count
Mean Cell Haemoglobin	Monocyte count
Mean Cell Haemoglobin Concentration	Eosinophil count
	Basophil count

Box 2.4.1: Haematological assays being performed on whole blood from 4ml EDTA vacutainers.

2.4.2.6 Urine vacutainers

The urine vacutainers will be transferred to a customised TECAN liquid handling platform configured to maintain the samples at 4°C. Six aliquots of urine (about 1.0ml each) will be transferred from each vacutainer into 2D bar-code labelled 1.4ml cryostorage tubes with split septum seals arrayed in 96 position racks (Figures 2.4.1a & b) according to Table 2.4.1 for long-term cryopreservation. The bar-codes on the 1.4ml sample storage tubes will be attributed to the bar-code on the vacutainer and the LIMS data set updated.

2.4.3 Cryopreservation of samples

Following processing, aliquot samples will be maintained at 4°C prior to transfer of the sample racks to either the automated -80°C working archive or manual -196°C liquid nitrogen back-up archive (as outlined in Section 1.5.5). Times and temperatures of all archiving operations and operator identifiers will be logged in the LIMS.

2.4.3.1 Automated -80°C working archive

Arrays of tubes in racks destined for the automated -80°C working archive will be loaded onto the archive loading trays and transferred to the loading buffer in the archive. Prior to entering the main chamber of the archive, they will pass into an environment purged with ultra-dry air (<3 ppm moisture); this is important to prevent frost build-up on the samples that could compromise the function of archive. After entering the archive, the bar-code on each tube and tube rack will be read. Racks will then be transferred automatically to empty

storage spaces in the storage units within the -80°C working archive. The location of each rack in the archive will be attributed to the rack bar-code and the tube bar-code. This record will be maintained in the independent archive inventory software and a message logged in the LIMS that the samples have been successfully stored.

2.4.3.2 Manual -196°C back-up archive

Sample racks destined for the liquid nitrogen back-up archive will be transferred to the archive site in temperature controlled shipping boxes at 4°C (or, in the case of the DMSO samples, on dry ice). Sample racks will be withdrawn one at a time and transferred to a storage tower in a liquid nitrogen vessel. The bar-codes for the liquid nitrogen vessel, the storage tower, and the storage tower shelf position will be attributed to the sample rack bar-code in the LIMS data set.

2.4.4 Withdrawal of samples from the archives

With the exception of the DMSO samples in the liquid nitrogen archive, any samples required for subsequent research will generally be withdrawn from the automated -80°C working archive. An approved sample set will be generated and the sample bar-codes identified from LIMS to produce an order which will be transferred to the archive inventory. The archive automation will retrieve racks containing the required tubes and transfer them to a tube picking station within the automated store (held at -20°C). Picked tubes will be transferred to an output rack which, when the order is complete, will be issued to the operator. Unpicked tubes in the racks will be returned to vacant storage location within the archive and the archive inventory updated to reflect the new situation. Issued samples will be aliquoted and sent to the laboratory conducting the assays (see Section 2.8), with any excess sample subsequently returned to the archive. The LIMS will maintain a record of the volume of sample used and the volume remaining; this will trigger replenishment from the back-up archive and help guide resource access decisions for depletable samples.

When samples in the automated archive need to be replaced from the back-up archive or DMSO samples are required for cell immortalization studies, a picking list will be generated from the LIMS indicating the exact location of the required samples. Tubes will be withdrawn from the liquid nitrogen vessel and assembled into an output rack held on dry ice; when the order is complete the accuracy of the order will be verified using a 2D bar-code reader. Issued samples will be transferred to the working store, or aliquoted and sent to the laboratory conducting the assays (see Section 2.8). All operations will be recorded in the LIMS, which will also maintain a record of the volume of sample used and the volume remaining.

2.5 Potential for enhancements

2.5.1 Repeat assessments in representative subsets

Typically in prospective studies of the relevance to disease of risk factors (such as blood pressure or blood lipids), various characteristics of the cohort are recorded at the initial "baseline" assessment visit and these baseline characteristics of individuals who subsequently develop a particular disease are then compared with those of individuals who do not. But, because of fluctuations in the *measured* values of a risk factor at baseline, such comparisons often substantially underestimate the strength of the real association between the *"usual"* (i.e. long-term average) level of that risk factor during a particular exposure period and the disease rate during that same, or a later, period [114]. This "regression dilution" effect may be caused by measurement error, by short-term biological variability (including both transient fluctuations and any diurnal or seasonal variation), or by longer-term within-person fluctuations in risk factor values (which may occur for several reasons, including physical activity, diet, treatment, disease or age).

Information from repeat measurements of the risk factor after just a year or two in a reasonably representative sample of individuals can be used to correct for the effects not only of random measurement error but also of short-term variability in risk factor levels. If, however, the aim is to estimate the usual risk factor levels 10 or 20 years later then corrections based on re-measurements made relatively soon after baseline may not allow properly for the effects of longer-term within-person variability. Moreover, since the interval between the baseline survey and the occurrence of an event in prospective studies is typically longer among those who suffer events at older ages, such underestimation may well be greater in the elderly. In order to make appropriate "time-dependent" corrections for these effects of regression dilution, re-measurements during prolonged follow-up can be used to estimate the usual risk factor levels at some particular fixed interval prior to death in each decade of age [49, 52]. In order to be able to adjust sufficiently reliably within various subsets of the cohort (e.g. for different ages at risk), such re-assessments need to involve at least a few tens of thousands of individuals on each occasion. Consequently, in UK Biobank, it is planned to repeat the baseline assessment (i.e. questionnaire, measurements and sample collection) in about 25,000 participants during the recruitment phase and then every 2-3 years during follow-up in a similar sized cohort.

2.5.2 Additional measures at re-assessment

Typically, in order to allow correction for regression dilution, the measures of interest made at baseline are repeated during the periodic re-assessments in representative samples of the cohort (Section 2.5.1). But, such repeat assessments can also provide an opportunity to conduct more intensive phenotyping of the participants being re-assessed. Whereas it might not be feasible (e.g. for reasons of cost) to undertake such intensive phenotyping in the whole cohort, more detailed assessment in several thousand individuals could still help to inform the whole cohort [115, 116]. For example, if for some reason it

was only feasible to estimate blood pressure as “below average”, “average” or “above average” (rather than to measure it directly) in all participants at baseline, then the informativeness of this estimate of blood pressure as a predictor of disease would be limited. But, if it was then possible to measure blood pressure in a representative subset of the cohort (e.g. during a subsequent re-assessment), these measured values could be used to determine the measured long-term usual blood pressure for each of the baseline-defined groups (i.e. below average/average/above average). That is, more precise measurement of some particular factor in a reasonably representative subset of the cohort would allow adjustment not only for regression dilution but also “calibration” for other sources of imprecision in baseline measures conducted in the cohort as a whole.

This calibration approach is likely to be particularly useful for various measures that it has not been possible to include in the baseline assessment of all participants in UK Biobank. For example, as described in Section 1.3.3.6, it is intended to develop an internet-based dietary recall questionnaire that could be completed by a substantial proportion of the cohort and so supplement the more limited food frequency information being sought in the whole cohort. Similarly, the repeat assessment visits planned for about 25,000 participants every few years (Section 2.5.1) provide an opportunity to conduct some more intensive measurements (e.g. the questionnaire-based estimates of physical activity being obtained at baseline could be supplemented by some more objective validated measure of energy expenditure, such as heart rate monitoring [117]). Development and conduct of the internet-based dietary recall questionnaire has been included in the budget for UK Biobank, and so too have the costs of repeating the standard baseline assessment visit every few years in about 25,000 participants. Separate funding will need to be sought, however, for the additional costs of conducting some more intensive measure in a subset of the participants attending for re-assessment. Given the potential value of such add on studies (and their relatively modest marginal costs), it seems likely that researchers interested in enhancing the UK Biobank resource in this way would be able to raise this funding through the regular peer-review mechanisms.

2.5.3 Intensive phenotyping at baseline

As discussed in Section 1.4.3, a large number of physical measures potentially associated with various health outcomes were excluded from the baseline assessment of the whole cohort for reasons of feasibility (i.e. available funding would not allow a more prolonged visit). These included electrocardiogram; continuous or ambulatory blood pressure and pulse rate; ankle-brachial index; pulse wave velocity; carotid intimal-medial thickness; cardiac echocardiogram; skinfold thickness; spirometry reversibility; bone densitometry; quadriceps strength; timed shuttle walk test; aggregated locomotor test; and visual and auditory acuity. Section 1.2 provides the rationale for recruiting at least 500,000 individuals aged 40-69 and following them for several years in order that there will be sufficient numbers of cases of any particular disease to allow the reliable assessment of plausible risk associations. Indeed, even with the more common conditions (such as coronary heart disease or diabetes), it is likely to require at least 5 years of follow-up before 5,000 cases have developed. But, as follow-up continues and more cases of these common conditions occur, more detailed

baseline measurements made in only a substantial subset of the whole population might well become informative. This would be the case especially if such measures were more precise and strongly related to health outcomes than those made in the whole cohort (e.g. heart rate monitoring rather than a questionnaire for physical activity) [118, 119].

As discussed above, variability and other sources of imprecision in the baseline assessment can be allowed for in UK Biobank by conducting repeat assessments that include some more precise measures in several thousand reasonably representative participants. As a complementary strategy, it has been proposed that some additional measures be conducted at baseline in about 100-200,000 of the participants. This option for an intensively phenotyped sub-cohort within UK Biobank has not been included in the budget and additional funding will need to be obtained to cover the full costs of its inclusion (including the impact on the assessment centre throughput and any changes to IT or other systems). Nor have there been detailed discussions as to what (if any) additional measures might be conducted in such an intensively phenotyped cohort. Instead, what is planned is that there be wide consultation during the early phase of recruitment among interested researchers in the UK (and elsewhere) as to what additional measures might be included. Funding will then be sought from relevant sources (e.g. heart disease charities for vascular outcomes; cancer charities for neoplastic outcomes) by those researchers, in collaboration with UK Biobank, with a view to incorporating these additional measures into the assessment visits during the latter phase of enrolment (e.g. the last 100-200,000 recruited).

2.6 Long-term follow-up

2.6.1 General approach

The value of the UK Biobank resource depends not only on its ability to obtain rich baseline data and samples but also on detailed follow-up of the health of participants through their medical records. Permission will be obtained at enrolment from all participants to access all of their past and future medical and other health-related records. These health records will be used to supplement information recorded at enrolment about previous medical history, family history, investigations (e.g. radiology reports, blood tests) and exposures (e.g. medication, occupational health). Most importantly, access to such records is needed to provide follow-up information related to cause-specific mortality and other health events (e.g. general practice consultations; out-patient and in-patient hospital activity; cancer and other disease registries; investigations; prescribing information).

A reliable mechanism is required for continuing to keep track of individual participant's health records during long-term follow-up. The most reliable single identifier is the NHS number in England and Wales and the Community Health number (CHNo) in Scotland. These identifying numbers are to be obtained for all potential participants prior to their invitation to attend the assessment centre. Other identifiers (such as name, date of birth, address, general practice) will also be obtained prior to invitation, and checked during enrolment, to allow linkage to other types of health-related information (such as occupational health records). Further information will also be sought during enrolment (including mobile telephone numbers and e-mail addresses). These different identifiers will help ensure that participants are not lost during follow-up, which may continue for many decades (e.g. the NHS tracing service can use the NHS number, or name and date of birth, to obtain updated GP details and address when people move).

A variety of different sources and systems will be used to ascertain death, disease occurrence and other health-related information among participants during long-term follow-up. Some of these systems have an established track record for long-term follow-up in epidemiological studies (i.e. death and cancer registries), whereas other systems have been used less widely in such circumstances (e.g. general practice and hospital activity records), although they have been successful in particular parts of the UK (e.g. Oxford Record Linkage Study; Scottish Morbidity Record). The NHS IT systems for Scotland are already sufficiently advanced to provide an electronic link to a wide range of relevant medical records, and a substantial effort is now ongoing to establish similar systems for the NHS in England and Wales. Linkage of participants within some of these systems will be initiated during the recruitment phase, but linkage to other systems will await further evolution of the central NHS IT systems. In either case, however, information will be sought from the relevant system about the participant's health from the time of their enrolment in UK Biobank and, where appropriate, from the period before recruitment (e.g. supplementing self-reported past medical history). The rest

of this section describes the current and likely future availability of different types of health-related information from these different sources and systems.

2.6.2 Death and cancer registries

In England and Wales, it is already possible to “flag” participants in research projects through the Office for National Statistics (ONS) in order to be notified regularly of all deaths and their certified causes (or embarkations when any participants emigrate). Similarly, in Scotland, such cause-specific information on all deaths is available from the Registrar General’s Office (RGO). Information about site-specific cancer incidence is also readily available from established registries of notified cancers in England and Wales through the ONS and in Scotland through the RGO. Such sources have been widely used in the UK for long-term follow-up of death and cancer in many previous epidemiological studies. Fact of death information from these sources is extremely complete, and the certified causes of deaths have also been shown to be suitably reliable for many epidemiological purposes. For example, among 2,500 deaths during one study, the certified underlying cause was confirmed in about 90% by information from other sources [120]. Moreover, information that is to be sought from other sources (such as hospital and GP records: see below) about events preceding death will be available, when needed, to help validate causes of death. It is intended, therefore, that follow-up of death and cancer incidence be initiated early during the recruitment phase of UK Biobank.

2.6.3 Hospital records

The UK Biobank data repository needs to include information about health events and activities that are experienced by participants when they attend hospitals. While the initial referral and other information about hospital activity is likely to be recorded within the primary care record, it is important that this should be supplemented by, and validated against, the information that can be derived from the hospital systems.

The Scottish Morbidity Record (SMR) has been collecting data on all admissions to all Scottish NHS hospitals since 1980, and these data are routinely collated by the Information and Statistics Division (ISD) of the Common Services Agency. UK Biobank’s Regional Collaborating Center for Scotland has access to methodology developed and implemented for the specific purpose of automatic retrieval of such information (e.g. the GENIE software application used successfully in the context of the national diabetes computing system). This software can be programmed to update all changes in health status for particular individuals on a daily, weekly or monthly basis by attaching an electronic flag to their CHNo in the electronic systems that hold the relevant health care information. Consequently, with the permission of the NHS Privacy Advisory Committee, UK Biobank will be able to extract hospital admission data for Scotland (and the same structures will also allow retrieval of primary care records, prescribing information, and maternity, cancer and death data: see below).

In the medium to long term, developments in the new National Care Record Service will also allow hospital activity data for England and Wales to be retrieved from a central source. Such information is already collected at a national level for other purposes: that is, the Department of Health's Hospital Episode Statistics (HES). HES is the national statistical data warehouse for England of the care provided by NHS hospitals and for NHS hospital patients treated elsewhere. It is the data source for a wide range of healthcare analysis for the NHS, Government, and many other organisations and individuals. Data held in HES are derived from the NHS-wide Clearing Service that provides the mechanism by which HES data are transferred from individual hospital trusts' clinical systems. For each financial year, there are approximately 12 million records (episodes of care) in the HES database, which represent all NHS-funded admissions for patient care, and private care within NHS hospitals in England. (Data are not included, however, about private health care, activity in Accident and Emergency departments, or drugs used during the hospital episode.)

For each episode of care, HES includes information about:

- Patient identifiers (including NHS number);
- In-patient, day case and out-patient episodes (with out-patient data having become mandatory in October 2001 and the mental health minimum dataset mandatory from April 2003), maternity records and psychiatric census;
- Administrative details (e.g. admission and discharge date) and the organisation providing the treatment;
- Clinical information relating to diagnoses (ICD10 codes) and procedures (OPCS4 codes).

As with the SMR in Scotland, HES retains historical data that can allow UK Biobank to supplement, and validate, the information obtained at enrolment about participants' past medical history. For example, cross-referencing of validated outcomes from regular clinic (and GP) follow-up showed a very high concordance (>90%) in the Heart Protection Study [120] with retrospective review of computerised hospital records.

Privacy of the individual is one of the basic principles behind the whole HES and SMR ethos. There are well described processes by which organisations can apply to receive this information, which is supplied as responses to specific query criteria and extracts from the core dataset. The nature of UK Biobank's request will entail special service agreements since the provision of clinical information in respect of identifiable patients is outside the normal areas of information provision to third parties. With respect to HES, SD2HES has obtained the agreement of the Security and Confidentiality Advisory Group to allow access to raw codes in specific circumstances; and, in Scotland, access to SMR data has previously been provided for such studies with the agreement of the NHS Privacy Advisory Committee. In both cases, the provision of these data to UK Biobank should be acceptable since all participants will have given signed consent at enrolment for extraction of their individual hospital records and other health-related information. It is intended,

therefore, that follow-up of hospital activity through HES and SMR be initiated during the recruitment phase of UK Biobank.

2.6.4 Primary care records

In Scotland, as discussed above, an individual's CHI number can already be used to link to a wide range of health-related information, including primary care, clinical and prescribing databases (e.g. GPASS in 85% of practices) going back to 1984, and systems have been developed for its automatic retrieval. Consequently, after obtaining permission from the NHS Privacy Advisory Committee and other relevant groups, it should be relatively straightforward for UK Biobank to extract general practice data for Scotland.

In England and Wales, there are numerous projects (e.g. Q-Research, EPIC/THIN and GPRD) that work directly with general practices and their current clinical system suppliers to retrieve practice data, but these do not provide national coverage. Instead, it will be more efficient to wait for the introduction of some of the infrastructure and applications that will be provided by the Connecting for Health (CfH) programme before national follow-up of primary care information for UK Biobank is started. The two key elements of CfH are the NHS Care Records Service and the Secondary Uses Service. The NHS Care Records Service will, in summary, contain the following components:

- ***Organisational records:*** The electronic equivalent of detailed paper records entered by clinicians and support staff to record and plan patient care within that organisation;

- ***Detailed care records:*** Where organisations share the same electronic records architecture within defined geographical areas, organisational records will be shared (within the constraints of access controls);

- ***Pathways of care and care plans:*** When patients have complex or chronic care needs, "pathways of care" will indicate the local care that is normally to be delivered (with multiple pathways of care applicable to those with co-morbidity). For each patient, a single shared care plan will be derived from their separate pathways of care. The care plan will contain key relevant past events for the patient (e.g. their blood pressure measurements, by whom and when) and their planned care (e.g. who is responsible for their blood pressure monitoring and when it will next be measured by whom). These pathways of care and care plans will be shared by all those caring for the patient.

- ***Summary Care Record:*** This will contain contributions from the general practice longitudinal record, hospital discharge and out patient summaries, pathology and imaging results and, in time, care by others (such as social care). The Summary Care Record will be widely available to appropriate health professionals through the Personal Spine Information Service.

UK Biobank should be able to access the data in the Summary Care Record, from the pathways and journeys of care and, in some situations, from the organisational and detailed care records.

The other key programme in CfH is the Secondary Uses Service (SUS), which aims to provide *“timely, pseudonymised patient-based data and information for purposes other than direct clinical care… [including] research”* [121]. SUS will access data from all sectors of the health service and social care, including general practice, community teams, secondary care hospitals, tertiary care and private providers supplying the NHS. It will have access to the data within the NHS Care Records Service and will be able to link it to external sources, such as registration of deaths, census data and health service organisational boundaries. Certain types of health data will not be available through SUS, including care from private providers, over-the-counter and complementary therapies, self care and care delivered overseas. But, for care delivered within the NHS in England, the geographic and organisational coverage of SUS data should be close to 100%. It has been confirmed with CfH that it will be possible to use the NHS number to track and extract clinical data from SUS for research participants who have given their consent (as in UK Biobank).

In order to ensure a robust and complete data set, UK Biobank will work with SMR in Scotland and with CfH in England and Wales to specify how access to routine clinical data for participants can be achieved (and, to that end, UK Biobank’s Chief Information Officer is working on secondment within the DoH, thereby enabling more direct discussions with the relevant parties). This will involve the initial specification of a historical and continuing dataset, with options for obtaining additional data from time to time to meet the specific needs of particular areas of research. During the recruitment phase, UK Biobank is likely to be able to initiate follow-up through primary care records during enrolment in Scotland and, at least, to have established and piloted the systems for such follow-up in England and Wales.

2.6.5 Self reporting by participants

An additional proven data source for capturing health events and medication in epidemiological studies is directly from participants during follow-up clinic visits [120] or via mailed questionnaires [120,122]. This approach has been shown to provide complete and reliable ascertainment of a wide range of health outcomes (e.g. in the Heart Protection Study, reports of serious vascular events or cancer by 1000 high-risk participants were shown to be more complete than those obtained from GPs). Although follow-up visits would not be feasible in UK Biobank, regular questionnaires (e.g. annual) could be used to supplement the other sources of information described above. Moreover, since the integrated pilot indicated that over 50% of participants would be willing to be contacted through their e-mail account, web-based follow-up would be a low-cost alternative to mailed questionnaires. This would allow participants to provide information about recent or current conditions (including those that might be under-reported in other data sources) and the drugs that they are actually taking during follow-up (i.e.

providing additional information relating to compliance and over-the-counter medications). These data could be cross-referenced with other information extracted from the health records of the participants to help minimise missing outcomes and to validate them.

2.6.6 Coding and validation

It will require several years of follow-up in UK Biobank before enough participants have developed any particular condition for reliable assessment of the main determinants of the condition (see Section 1.2). The initial recruitment phase and early years of follow-up will allow the careful development and piloting of systems for accessing and validating data from a variety of different systems. Consequently, by the time sufficient numbers of events have occurred among the participants, UK Biobank will have validated data on a wide range of health outcomes that is sufficiently reliable and complete for the purposes of most research (and that can be readily supplemented in particular ways when required for specific purposes).

Currently, the accuracy and completeness of the data available through health care records systems is variable, and one of the principal aims of CfH is to improve data standards and consistency. Most is known about the quality of general practice data, where early adoption of computerised systems has resulted in data quality that is often higher than in other sectors. Almost all general practices in the UK are already computerised [123], and up to two thirds are now using their clinical computer as the only means of recording clinical care (including encounters, diagnoses, prescriptions, etc) [124, 125]. Moreover, the Quality and Outcomes Framework of the new General Medical Services contract for general practices has stimulated efforts to improve accuracy and completeness [126]. Although UK Biobank may need to access some free-text entries in order to establish the exact nature of a health care event or decision, it will primarily use the capture and analysis of codes. Experience with Read codes shows some variability in their use [127-130], but further education and training should help to ensure the effective implementation of Snomed codes [131, 132]. More problematic is the exact meaning of certain terms: for example, while there are internationally agreed diagnostic criteria for myocardial infarction (and the patient’s record is likely to include evidence that those criteria have been met: see below), no such criteria are routinely applied to post-natal depression. Moreover, clinicians are skilled at interpreting such diagnoses in their historical context (willingness to make diagnoses and use certain labels changes with time) and according to the background of the person generating the entry (e.g. different weights may be given to a label of postnatal depression that is applied by a consultant psychiatrist, obstetrician, GP or community midwife). As the health services become more reliant on electronic health records, they are shared more widely and such deficiencies become more evident. For example, analyses through SUS have revealed variations in the quality of data recording which educational initiatives (such as PRIMIS+) are now working to rectify.

For UK Biobank, clinical research staff will develop and implement procedures for identification and cross-validation of outcomes from different healthcare

sources. It will be important to start the process of identification and validation of health outcomes during the latter part of the recruitment phase so that their coverage becomes comprehensive during the subsequent 5 year period when the resource starts to become sufficiently mature for informative case-control studies of the commoner conditions (such as heart disease). As multiple sources of information about health events (e.g. primary care; hospital activity; investigations; prescriptions) become available to UK Biobank, it will be possible to build a range of semi-automatic systems for the confirmation or refutation of a wide range of outcomes that should suffice for many research purposes. For example, myocardial infarction identified from the primary care record might then be supported by a confirmatory hospital discharge record and/or by an electrocardiogram or laboratory report consistent with myocardial infarction (or, alternatively, refuted by a discharge record or investigations more consistent with, say, unstable angina). Similarly, cancer registry data may not only be confirmed but also made more specific by linking them to relevant laboratory systems (e.g. histology). These approaches will build on research that is currently being supported through the MRC's e-science program (such as the VOTES project, which involves UK Biobank's RCCs). Even where such automated systems are not able to provide sufficiently specific information about the type of health outcome (at least in the short term before all relevant records can be accessed), they should be able to identify a suitably limited group of individuals for whom particular information needs to be sought.

Follow-up data will be appended to the UK Biobank core data repository, and linked to pre-existing data (such as assessment visit records) primarily through indirect linkage using the participant's NHS number (validated by reference to other information, such as name and address). Some datasets may not include the NHS number, which will necessitate an auditable comparison of supplied data with other identifying data for participants (e.g. name, address and date of birth). Data that are to be included within the repository will initially be transformed into a standards based format (see Section 2.7.3.5), keeping coding structures, values and textual data in their original form in order to ensure an audit trail back to the source data. Imported data will then provide the basis for ongoing clinical validation and cross-referencing with any previously supplied data residing within the core systems. If appropriate, data may be re-coded (e.g. by conversion to a standardised coding system) or summarised to aid high-level search and querying processes which will provide more consistent information sets for subsequent data-mining and other research activities. All clinical cross-referencing and re-coding work will be performed under secure conditions, without direct reference to information that identifies a participant (such as name and address). Since initial tests on primary care data have shown high variability in the quality of coded data, it is highly likely that any automated processes will require auditable human validation and sign-off before being included within the core repository and made available for research.

2.6.7 Participant withdrawal

Participants will be advised at enrolment that they have the right to withdraw at any time without giving a reason and without penalty. This is essential to preserve and demonstrate the voluntary nature of participation. UK Biobank will explain the options for withdrawal:

- ***"No further contact":*** This means that UK Biobank would no longer contact the participant directly, but would still have their permission to use information and samples provided previously and to obtain further information from their health-relevant records.

- ***"No further access":*** This means that UK Biobank would no longer contact the participant or obtain information from their health-relevant records in the future, but would still have their permission to use the information and samples provided previously.

- ***"No further use":*** This means that, in addition to no longer contacting the participant or obtaining further information, UK Biobank would aim to destroy all of their information and samples collected previously (although the participant would be told that it may not be possible to trace all distributed sample remnants for destruction). Such a withdrawal would prevent information about them from contributing to further analyses, but it would not be feasible to remove their data from analyses that had already been done.

If, having discussed their concerns and options, a participant decides to withdraw then UK Biobank will seek written confirmation of the level of withdrawal from the participant. UK Biobank will need to retain some minimal personal data on such individuals for a number of reasons, which include: ensuring that participants who have withdrawn are not re-contacted; and assessing the determinants of withdrawal and any impact on research findings. Participants who withdraw will be assured that this administrative record will not be part of the main database that is available to others.

UK Biobank will not enrol potential participants who express the view that they would want to withdraw should they lose mental capacity or die, because this would reduce the value of the resource for research. But, if a participant decides some time after enrolment that he or she would wish to be withdrawn in the event of incapacity or death then this request will still be honoured and their consent modified accordingly. If a participant loses mental capacity or dies, UK Biobank will be guided by the most recent record of the participant's consent. Family members will not be able to withdraw incapacitated or deceased relatives unless the participant's consent was amended accordingly beforehand. In all events, UK Biobank will safeguard the confidentiality and security of participants' data and samples as long as it holds them, including after a person's death.

2.7 Data handling and security

2.7.1 Overview

The data that are to be used by UK Biobank are of the highest sensitivity and, as such, need to be handled with the greatest care. Security is a prime concern, especially during transit. It is essential that UK Biobank is compliant with the requirements of relevant legislation, such as the Data Protection Act (DPA), and also meets the needs of other relevant groups, such as the Patient Information Advisory Group (PIAG) and the CHI Advisory Group. It is unlikely that UK Biobank will be able to gain access to the broad range of third party data sets required, or be able to provide validated research data, if these external requirements are not taken into account. Key aspects of the controls required include identity and identifier management, ensuring the accuracy of the data collected, inclusion of comprehensive audit data (such as the staff and equipment involved in data collection) and strict controls on data access.

From a data handling and security perspective, UK Biobank activities relating to the collection and use of data on participants have been defined under the following broad headings:

- ***Invitation and recruitment:*** This covers the initial records supplied by the NHS, invitation mailings and pre-assessment operations (including the telephone information service);

- ***Assessment centre data collection:*** Data collected during a participant's visit to an assessment centre include the informed consent necessary to allow retention and updating of data in the repository;

- ***Laboratory operations*****:** Samples collected during the assessment centre visit and stored in the coordinating centre sample archive, along with related data in the Laboratory Information Management System (LIMS);

- ***Core operations*****:** Also housed at the coordinating centre, the core repository will securely store and maintain all collected and interpreted clinical data relating to participants;

- ***Participant health records*****:** Subsequent information collected from health records will be validated and appended to the core repository;

- ***Research management*****:** Data provided to researchers will need to be controlled in order to prevent inadvertent disclosure of identity and ensure acceptable usage.

2.7.2 Systems architecture

Figure 2.7.1 illustrates the conceptual components which make up the overall UK Biobank systems architecture. The subsystems and processes shown

have been developed from experience gained during piloting operations. It is important to note that the architecture is specifically not designed as an interactive environment where people (for example in assessment centres or the call centre) have any access to data stored in core systems. Access to these systems will only be permitted to a limited number of named UK Biobank staff (or designates) under controlled conditions.

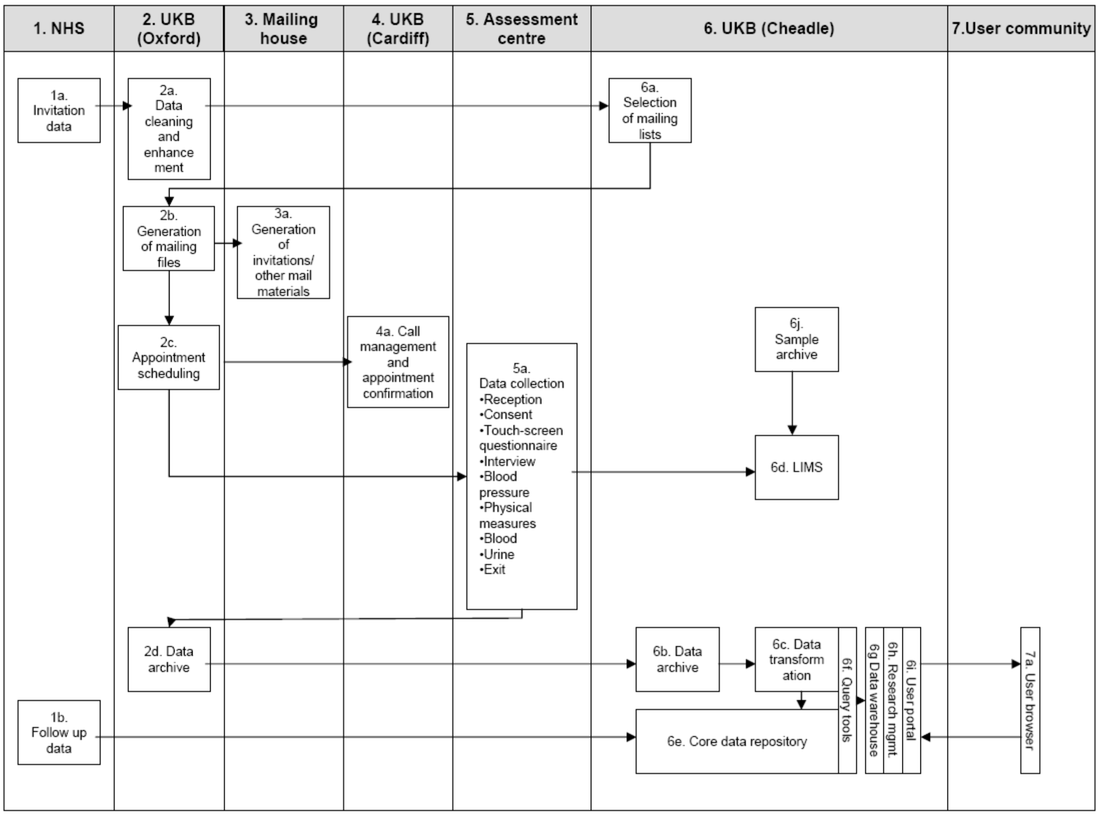

Figure 2.7.1: Key components of the UK Biobank systems architecture

In summary form, the NHS will supply lists of people to the UK Biobank coordinating centre in Cheadle (UK Biobank [Cheadle]). The Clinical Trial Service Unit in Oxford (UK Biobank [Oxford]) will then provide systems for the management of mailing lists, call centre operations and the initial participant details that are supplied to assessment centres. Assessment centres will collect informed consent and subsequent clinical data from the participants into bespoke IT systems. These data will be passed to UK Biobank for inclusion in the core repositories. UK Biobank will subsequently obtain follow-up data from medical and other health-related records systems. Repository data will then be validated before being made available for research purposes. The above processes are detailed in the following section, with particular emphasis on identifying data.

2.7.3 Data Handling

The major components shown in the diagram in Section 2.7.2 will handle data as below. Reference has been made to the operational components in the diagram by appending the component number in brackets.

2.7.3.1 Invitation and recruitment

NHS Invitation Data (1a) will be forwarded to UK Biobank Initial Clearing (6a) under conditions agreed with the NHS. Dependant upon the NHS data set supplied, an Office for National Statistics (or equivalent) check may be performed to remove deceased or unknown records and a separately cross-referenced unique identifier [UKB-ID-01] added.

This modified and checked data set will then be passed to the Invitation System (2a) hosted at UK Biobank [Oxford]. A new identifier [UKB-ID-02] will be assigned to invitees for use in appointment booking, mailing, and call management operations (2b, 3a, 4a). A secure web-based interface will be provided to nominated UK Biobank staff in order to generate invitation (and any subsequent re-invitation, DNA and post-visit) mailing data sets which will be passed on to the Mailing System (3a). The Invitation System will separately supply the identifier linkage [UKB-ID-01 to UKB-ID-02] information to UK Biobank, which will ensure that participant data are transmitted separately to data that allow linkage back to the NHS Number.

The Participant Booking system (2b) will be securely hosted by UK Biobank [Oxford]. A secure web-based interface (https) will be provided to the Call Management (4a) operations hosted at the Welsh Regional Collaborating Centre for UK Biobank (UK Biobank [Cardiff]), and to other nominated UK Biobank staff, for the management of invitees prior to their assessment visit.

2.7.3.2 Assessment centre data collection

Periodically, the Booking System (2b) will securely transfer appointment data (including name, date of birth, gender, address, and UKB-ID-02, but not the NHS number) to bespoke Assessment Data Collection systems (5a) in the relevant assessment centre. The Data Collection system will also be provided with security-related information to control access to the system by assessment centre staff and prevent unauthorised access. When a participant registers at the reception station, the Assessment Data Collection System will first collect informed consent from the participant. Data will then be collected as they proceed through the following modules: self-administered touch-screen questionnaire; interviewer questionnaire; physical measurements (blood pressure, grip strength, weight, height, impedance, spirometry); and blood and urine collection. Finally participants receive a copy of their consent form, a key measurements report and a travel expenses form before their departure. Between the different visit stations, each participant transfer their encrypted data on a dedicated USB key, which also provides a temporary back-up for the assessment centre system (before the key is retrieved and wiped clean at the end of the visit). Assessment centre staff or users will not be able to view or alter collected data retrospectively.

2.7.3.3 Laboratory operations

Blood and urine samples will be initially processed within the assessment centre and then shipped to UK Biobank [Cheadle] at the end of each day for

further processing and archiving within the sample archive (7a). Participant and vacutainer identifiers will be securely transferred to UK Biobank [Cheadle] in order to enable logging of received samples into the secure LIMS (7b). Before the LIMS receives these data, the participant identifier [UKB-ID-02] will be replaced with a LIMS specific identifier [UKB-ID-03]. This will ensure that aliquot-related data cannot be directly linked to the participant identifiers used in other operational areas, whilst enabling the laboratory to begin their archiving operations by checking that the correct vacutainers have been received and processed in an auditable manner. Participant identifying data (such as name and address) will not be available to the LIMS.

2.7.3.4 Core operations

Because of the distributed nature of UK Biobank assessment centres, it is necessary to return clinical data to central operations in order to provide timely and regular audits that data are being collected correctly and to provide the necessary feedback for efficient and flexible pre-assessment operations. On a daily basis, the Assessment Data Collection systems (5a) will securely transfer encrypted assessment data to the intermediate Assessment Archive (2c) for initial data validation and unpacking. This will enable UK Biobank [Oxford] to provide rapid responses, and any necessary improvements required, for the smooth running of the assessment centre systems.

The Assessment Archive will periodically provide validated assessment data to UK Biobank [Cheadle], either using secure file transfer or on encrypted CD-ROM. Using dataset specific transformation services (7c) residing separately from the core repository, data will be unpacked and transformed into, and validated against, a standards-based Health Level 7 (HL7) format. This will incorporate audit data, such as the staff responsible and equipment used during the data collection process. HL7 is an internationally developed information standard that has gained wide acceptance, and is being used by the NHS as the basis for ongoing national developments (such as Connecting for Health) and is referenced by European standards (such as CEN [/TC 251] and the openEHR initiative). The controlled and auditable processing of data using standards-based transformation and validation services that comply with internationally recognised information standards maximises the likelihood of UK Biobank being able to provide data of certifiably high standard, and increases the potential for future interoperability.

When the assessment data have been successfully transformed and validated, they will be deposited into the highly secure Core Repository (7d) which will form the basis of the long-term UK Biobank data store. It is necessary to deposit the various data sets supplied to UK Biobank in a single location, not only to ensure consistent quality but also to maximise the potential value of participant-related data received from multiple disparate sources and to provide a "central authority" for managing and protecting these sensitive data.

2.7.3.5 Participant health records

Validated and deposited assessment data will subsequently provide the trigger for requesting medical and other health-related records from the NHS and other sources (chiefly for longitudinal follow-up but also for enhancing the baseline assessment). The participant identifiers used for assessment purposes will be mapped back to NHS numbers in order to generate requests for data from health record sources. Health records will be assigned a new specific identifier [UKB-ID-04]. Where separate sources of health records are provided (for example non-NHS cancer registries), a new identifier will also be provided in order to ensure the separation of data sources within the repository. The health records data that are to be provided to UK Biobank will form the bulk of information stored within core systems and will provide the essential longitudinal information necessary to enable further ongoing research. Further details on health records and the strategy to be adopted for linking to them can be found in Section 2.6.

It is currently planned that linkages to a participant's NHS number and name/address data will be stored separately to the Core Repository (7d), within the UK Biobank Clearing function (6a, 6b). On receipt of health records by UK Biobank Clearing and subsequent replacement of the NHS number, data will be transformed into, and validated against, a standards-based format before being appended to the Core Repository. Whilst the Core Repository would be sufficiently secure to hold these participant identifying data, it may be preferable to store such data separate from any sensitive records (such as health information). This would, however, induce extra overheads for UK Biobank Clearing operations (6b) when requesting health record data.

2.7.3.6 Research management

Validated research requests will provide the parameters necessary to generate appropriate limited data sets containing only the necessary data to answer a particular research question (Data Warehouses). Disclosure control and identifier replacement [UKB-ID-nn] will be performed on these warehouses in order to ensure that the data included do not enable the identification of participants. These data will then be made available for Research Management. Further details on research management and the higher level strategy for allowing access to research data can be found in Section 2.8.

2.7.4 Controlled linkage to identifiers and consent validation

The key that links the UK Biobank participant identifiers to publicly available identifiers (e.g. name, address, NHS number) will be stored separately from the tables that store medical and other sensitive data. This linkage information will be accessed as data flows are received in order to check that information received relates to consenting UK Biobank participants. Human access to this linkage information will be subject to the strictest controls, with the minimum numbers of named individuals authorized to access it and then only under

strictly defined conditions. In practice, linkage tables may be stored on a physically separate partition of the UK Biobank core storage, or even on a completely separate hardware platform remote from the core systems.

In order to protect the rights of participants, UK Biobank information management processes must validate consent when data are transferred between systems and before they are used for research purposes. The information management processes also need to be able to deal appropriately with withdrawal of consent by participants, including the different levels of withdrawal (see Section 2.6.7)

2.7.5 Security and Resilience

A high level of information security and resilience is a primary requirement for the ongoing viability of UK Biobank as a usable resource. Any compromise of the information systems may invalidate its operation and seriously affect public perception of UK Biobank as a project worthy of participation. Moreover, a lack of resilience may mean that, in the event of a disaster, the resource becomes compromised or unavailable for further use.

The processing and storage components of UK Biobank systems will be hosted in dedicated facilities. Strict controls over physical and logical access will be implemented which permit access only to authorised individuals. Consideration will be given to resilience issues such as off-site backup and escrow facilities to facilitate the resumption of operations in the event of system failure or disaster. UK Biobank is currently developing a detailed Information Security Management System with external experts, working towards ISO 27001 compliance. This will put in place a set of controls, consisting primarily of policies and procedures, to manage:

- ***Overall security:*** An information security governance structure that provides strategic direction and implements the high level processes for monitoring the success or failure of the underlying security processes. This is comparable to the high level PDCA (Plan, Do, Check, Act) processes implemented in a Quality Management System.

- ***Organisational assets:*** Understanding what information assets are held, and managing their security appropriately. Policies and procedures will cover the classification of information, and its appropriate handling by UK Biobank, to ensure that sensitive data are not compromised.

- ***Communications and operations*:** Security controls for systems and network management will ensure that IT systems are configured and used in a secure manner, mitigating against intrusion and failure. Control of logical access to IT systems, networks and data will prevent unauthorized use.

- ***Human resources security*:** Access rights for staff, including acceptable usage policies and suitable security awareness and training activities.

- ***Physical and environmental security*:** Protection of valuable IT systems against malicious or accidental damage, or loss through overheating or mains power failure. Use of equipment will need to be controlled and monitored in order to ensure that the data collected by, or stored on, this equipment are accurate and not compromised.

- ***Systems development and maintenance:*** Taking information security into account in the processes for specifying, building/acquiring, testing and implementing IT systems.

- ***Security incidents:*** Prompt reporting and proper management of information security events, incidents and weaknesses (including near-misses) provides a key feedback mechanism for the monitoring and improvement of information security systems.

All policy and procedure documents will be integrated with the Quality Management System being developed by UK Biobank laboratory operations.

2.8 Strategy for access

2.8.1 General approach

It is anticipated that the UK Biobank resource will chiefly (although not solely) be used to assess the relevance of different exposures through a series of case-control or case-cohort studies of particular health outcomes “nested” within the cohort. By comparing the answers, measurements and samples collected at baseline from participants who develop some particular disease during follow-up with those from apparently similar non-diseased controls selected from within the same cohort, it should be possible to work out why some people develop the disease of interest while others do not. This strategy has the advantage that most biological assays (other than haematology which cannot use stored samples) will only need to be conducted on baseline blood and urine samples from cases of the particular disease and from their matched controls. Consequently, it allows assays to be performed more cost-effectively on a relatively small subset of the cohort (e.g. a few thousand or tens of thousands people, rather than all 500,000), which also facilitates good quality control. Even in a cohort of 500,000 individuals, it will take several years before sufficient numbers have developed any particular disease to allow reliable statistical analyses (see Section 1.2). Consequently, this approach has the additional advantage that decisions about what assays to perform need only be made some years in the future when specific hypotheses will be clearer than at the time of collection, and the range of assays 0that can be conducted with available resources is much wider. It also means that there are largely predictable timelines when the resource is likely to become mature for particular conditions based on their differing incidence rates, which allows a coordinated approach to the use of the resource.

2.8.2 Coordination of resource use

The UK Biobank sample resource is finite and it is likely to be in considerable demand from academic and commercial groups in the UK and internationally. Consequently, it will need careful management to ensure the greatest scientific value can be extracted in order to achieve UK Biobank’s long-term aims. Prioritisation of requests for access to the resource will be determined according to strategies, processes and criteria for prioritisation (i.e. an Access Policy) to be set by the Board of Directors of UK Biobank on advice from its Steering Committee and an Access Committee (see below), and in consultation with the Ethics & Governance Council and the International Scientific Advisory Board. In particular, it will be important to:

- Involve leading academic and commercial researchers in the UK and internationally in advising on the best use of the resource to address key scientific questions now and in the future across the entire spectrum of disease research.

- Develop and monitor review processes that objectively address similar proposals for studies in similar disease areas, and that also provide balance between proposals for access for studies in different areas.

- Manage the use of the resource over time in order to avoid depletion of samples on a “first-come first-served” basis, which might otherwise prevent opportunities to answer fundamental questions in the future.

- Establish clear and controlled processes from receipt of a proposal for access to samples/data to delivery of information to the researcher (with the possibility of different procedures for requests that involve access to data alone compared to those requiring sample assays).

- Monitor the output from research on the resource to ensure that the overall aims of UK Biobank are tangibly achieved.

Based on the anticipated incidence rates for a range of different conditions and plausible estimates of importantly relevant exposure associations (see Section 1.2), a timetable will be developed to indicate when the UK Biobank resource is likely to be sufficiently mature to establish case-control collections for each condition (and the frequency at which it is likely to be worthwhile updating such collections). Researchers would then be able to develop their proposals against this indicative timetable, which UK Biobank could use to guide its calls for use of the resource and to plan its work schedules (e.g. sample retrieval). Given the limited and depletable nature of the blood and urine samples (other than the DNA, which can be amplified and replenished: see Section 1.5), it is essential that their use is carefully controlled in order to maximise the informativeness of the resource in the long-term. Such scheduling of access for each condition would help ensure the more efficient use of the resource: for example, case-control sample sets could be established and updated in a planned way (rather than unduly frequently in response to separate requests) and a wide range of assays required by many different researchers could be conducted in a coordinated fashion at one or a few laboratories (rather than sending separate aliquots to a large number of different laboratories, with each doing just a few assays). Due to the different underlying incidence rates, the resource will mature in this way at different times for different conditions of interest. Consequently, it should generally be possible to smooth the main activities over time and to focus attention on just a few conditions at any one time.

2.8.3 Review of access proposals

UK Biobank aims to encourage and provide wide access to the resource for researchers from the academic, commercial, charity and public sectors, both nationally and internationally, in order to maximise its value for health. It is important that the application process for access is fair, open, transparent and streamlined, and that it includes suitable methods for managing conflicting interests. All applications for access to the resource are to be judged on their merit (bearing in mind the depletable aspects of some parts of the resource), and exclusive access to any part of the resource will not be provided to any user. As discussed above, UK Biobank’s Board will develop the detailed processes for assessment of proposals based on advice from its Steering Committee and an Access Committee. The nested case-control approach

allows calls for proposals in particular disease areas to be advertised by UK Biobank in accordance with indicative timelines made public in advance. Review of these disease-specific proposals can then be conducted by *ad hoc* groups of independent experts in the particular disease area. Based on their advice, prioritisation of proposals from different disease areas can then be considered by a more general Access Committee which would consider wider issues (e.g. depletion of the resource and long-term needs) and advise UK Biobank's Board accordingly. This Access Committee, and all such *ad hoc* groups, would need to be broadly representative of relevant areas of UK science. In particular, although individuals might be included from one or other of the Regional Collaborating Consortia, it is essential that other relevant national and international experts are involved. Both the Ethics & Governance Council and the International Scientific Advisory Board will have oversight roles with respect to the timetable for proposals, the review process, the access recommendations, and the outcomes of approved research.

2.8.4 Access agreements and fees

As a condition of access to relevant data (i.e. assay results, physical measures, or questionnaire responses) from the resource, the approved researcher would be required to enter into an access agreement with UK Biobank. This would detail the specific purposes for which use of the data has been agreed and standard terms relating to exploitation and dissemination of results. Similarly, when samples are provided to a laboratory for assays, a materials transfer agreement will require that the samples are used for the agreed purposes only and that the results of the assays are returned to UK Biobank within specified time limits. Information identifying participants will be removed before any data or samples are released, and the agreements will include an undertaking not to attempt to identify participants. UK Biobank will generally permit exclusive use of the relevant data set for a limited period from its release in order to allow time for the approved researcher to conduct and report the agreed analyses. Subsequently, the results will be incorporated into the resource database for use by other approved researchers. Access to the resource will not be permitted for police use, except where required by court order, and UK Biobank will resist access for this use (in particular by seeking to be represented in all court applications for such access). A system for monitoring compliance with the terms of the access agreement will be put in place before the resource becomes available for access, and a policy developed for dealing with non-compliance (e.g. restrictions on future access).

It is anticipated that a data access fee will generally be charged for access to the UK Biobank resource. The chief aim of this fee will be to cover the costs of any sample and/or data retrieval, preparation and analysis required for the particular research use and to help cover the costs of maintaining the resource for future users. The Board will determine a fee structure which, in keeping with UK Biobank's charitable status, is set at a level that does not discourage use. Fees for commercial use may be higher than those for non-commercial use, although consideration will be given to the impact of this on the full range of potential uses (including, for example, by smaller companies or innovative uses in large companies) and the difficulties of applying such

differential fees in practice (particularly given collaborations between non-commercial and commercial users).

2.8.5 Dissemination of results

UK Biobank's Board will develop the detailed processes related to the dissemination of results. Researchers who use the UK Biobank resource will be required to disseminate the results of their research as rapidly and widely as possible, subject to ethics and confidentiality considerations. They will be encouraged to discuss their research findings with other scientists and the public, and to share relevant data and materials as openly as possible. Laboratories and other users who have had access to samples will be required to provide details of the assay techniques used. A limited delay prior to the dissemination of findings will be permitted in order to enable a paper to be published, a patent to be filed or other competitive advantage to be pursued. Users will be required to undertake to notify UK Biobank in advance of publishing such findings, to acknowledge the contribution of the resource, and to provide a copy of any published reports. In addition, researchers will be required to provide UK Biobank with a copy of all of the results of their research based on the resource (including any negative findings and relevant supporting data) for incorporation into the central database.

2.9 Organisation

2.9.1 Overall structure

UK Biobank has been established as a non-profit making charitable company limited by guarantee, and is funded by the Department of Health, Medical Research Council, Scottish Executive and North West Regional Development Agency, and by the Wellcome Trust research charity. It is also supported by other health research charities, such as the British Heart Foundation and Cancer Research UK, as well as by the National Health Service and the Royal College of General Practitioners. Several discrete elements are involved in management and advisory roles (Figure 2.9.1).

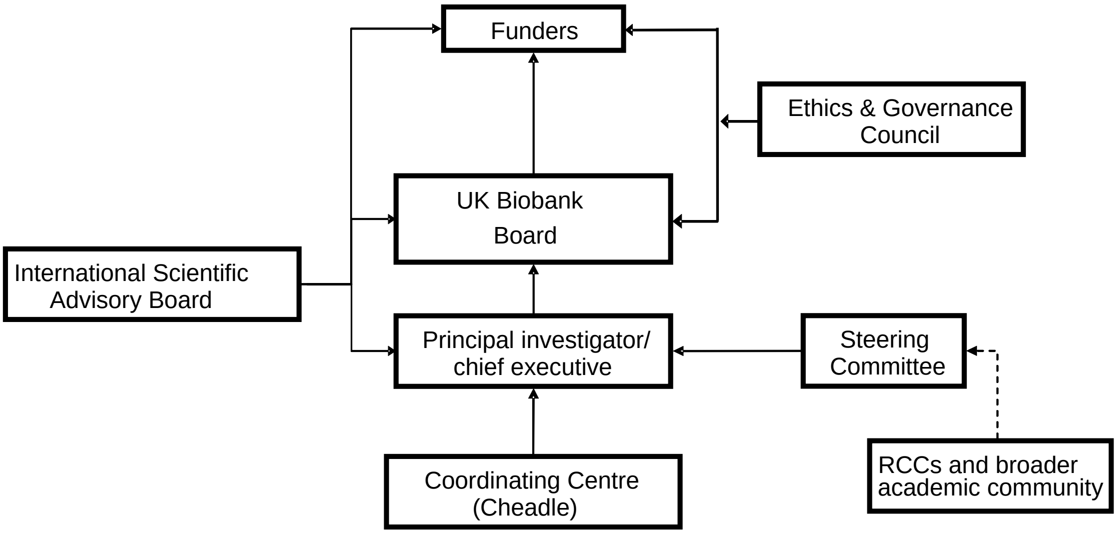

Figure 2.9.1: Management and governance structure for UK Biobank

Ultimate responsibility for delivering the resource, ensuring careful budgetary and corporate governance, falls to the Board of UK Biobank. The Board is chaired by Sir Alan Langlands, who was previously Chief Executive of the NHS and is now Principal and Vice Chancellor of the University of Dundee. The Board delegates responsibility for UK Biobank's design and conduct to the Principal Investigator/Chief Executive (PI/CEO), Professor Rory Collins, who is BHF Professor of Medicine & Epidemiology at Oxford University.

UK Biobank's coordinating centre is based at Manchester University. This national initiative involves the collaboration of over 20 UK universities (see Annex 1), with several other universities also contributing. Representatives of the six Regional Collaborating Consortia (RCC) form the Steering Committee which advises the Principal Investigator on scientific aspects of the resource. They also act as a link to consortium members, and the wider academic community, in order to facilitate national recruitment and access the best scientific advice.

An International Scientific Advisory Board has also been established, chaired by Professor Stephen MacMahon (Director of the George Institute at Sydney University), to provide further scientific advice to the Steering Committee, Board and funders. Guidance on the way in which the resource is established and used is provided by an independent Ethics & Governance Council, chaired by Professor Graeme Laurie, (Professor of Medical Jurisprudence at Edinburgh University). Finally, UK Biobank’s research activities are currently being reviewed by the NHS Northwest Multicentre Research Ethics Committee (MREC) to ensure that they meet the required standards for conducting research using human volunteers in the UK.

2.9.2 UK Biobank Board

Members of the Board are appointed by the Wellcome Trust and the MRC, or by the Board itself with the agreement of these funders. In addition, the Scottish Executive, Department of Health and Manchester University are each entitled to appoint one member (see Annex 1 for membership).

The Board is responsible for the overall management and operation of UK Biobank and for complying with all company law, charity law and statutory and regulatory obligations. It is also responsible to the funders for ensuring that the resource achieves its scientific objectives within the available budget, that all appropriate ethics approvals are obtained and complied with, and that the resource is used appropriately. All of UK Biobank's legal powers are vested in the Board, although the Board can and does delegate certain of its powers to committees (including the Audit Committee and the Remuneration Committee) and to the CEO/Principal Investigator. The Board has adopted a formal schedule of matters reserved for its approval, and remains directly responsible for overall governance issues, risk management, the adoption of budgets and business plans, changes in structure, and the approval of contracts or commitments exceeding a designated amount. The Board also retains responsibility for approving the protocol and associated policies, including the Access Policy and Ethics & Governance Framework (although these documents are subject to Wellcome Trust and MRC approval).

Scientific advice is received from the Steering Committee through the CEO/PI, and will also be provided by the International Scientific Advisory Board (ISAB). The Ethics & Governance Council (EGC) provides the Board with advice on ethics and governance issues relating to the UK Biobank resource.

2.9.3 Steering Committee and Regional Collaborating Consortia

The Steering Committee is chaired by the Principal Investigator (PI). Membership includes the lead investigator from each RCC, with UK Biobank’s Executive Director and Chief Scientific Officer as observers (see Annex 1). The Steering Committee is responsible for advising the PI on the development of the scientific protocol, and on the direction and scientific objectives of UK Biobank. In particular, it provides scientific input into the location of the assessment centres; the recruitment and monitoring of assessment centre staff; the identification, recruitment and processing of participants; and the

questionnaire and baseline measures at the assessment centre visit. It has also been responsible for defining the sample collection, processing and archiving strategy, including the decision to implement an automated working store and manual back-up store. As development of the resource progresses, the Steering Committee will support UK Biobank in the development of approaches for follow-up of participants' health records, for adjudication of health outcomes and for repeat assessments of participants. There will also be opportunities to consider, in collaboration with individual members of the RCCs and the wider scientific community, possible enhancements to the baseline assessment (see Section 2.5). It will be part of the role of the Steering Committee to review the likely costs, value and implications of such enhancements, and to work with researchers to identify possible sources of additional funding.

The relationship between UK Biobank and the RCCs has been established under a standard collaborative research agreement. Each RCC provides scientific input through the Steering Committee. Members of individual RCCs may also provide additional specialist skills required by the project as a whole. Having a single group responsible for such activities (rather than replicating them at each RCC) should help to reduce costs and improve consistency. The Scottish RCC is providing expert training and monitoring for all assessment centre staff and the Welsh RCC will provide the central information and appointment telephone service for potential participants. These two areas exemplify the "added value" of the RCCs. As UK Biobank progresses, there will be more such centralised activities (such as follow-up and adjudication of heath outcomes) that can be centralised at one or more RCC. Moreover, UK Biobank's collaborations are not confined to academic institutions associated with an RCC. It has already consulted widely with the broader academic community in the United Kingdom (and elsewhere) to obtain expert advice on specific scientific aspects of the resource design. Assessment centres in centres of population not directly associated with an RCC consortium may be established through other academic organisations. Moreover, opportunities for enhancing the baseline assessment (e.g. internet-based diet diaries; intensive baseline or repeat assessments in subsets: see Section 2.5) will be explored with the UK and international scientific community.

2.9.4 Coordinating Centre

The UK Biobank coordinating centre in Manchester is responsible for a number of areas:

- ***Management of the identification and invitation of participants:*** Using the lists of potential participants provided by health agencies, staff at the coordinating centre will run the mailing programme to ensure participant throughput at the assessment centres in the various locations is maintained at a high level. This will require procurement and management of a large-scale printing and mailing operation in partnership with a commercial supplier. Management of the mailing programme will be done in close collaboration with the appointment scheduling and management systems in the information call centre.

- ***Establishment and management of the assessment centres:*** In parallel with the recruitment strategy determined by the principal investigator, the coordinating centre will identify and procure facilities suitable for assessment centres. The coordinating centre will commission the facilities and manage them on a day-to-day basis until they are ready to be de-commissioned and moved to another location. It will also be responsible for the recruitment and management of assessment centre staff over the course of the recruitment period.

- ***Implementation and operation of the high throughput sample processing laboratory:*** The laboratory group at the coordinating centre have designed and implemented a high throughput sample processing laboratory. This will be used to process the large numbers of participant samples at high throughput and quality.

- ***Sample archiving:*** Once the participant samples have been processed they will be archived in ultra low temperature stores either in the -80°C automated sample store in Cheadle (at the coordinating centre) or in the liquid nitrogen back up store in Wythenshawe (approximately 5 miles from the Cheadle site). The coordinating centre will be responsible for running and maintaining these stores during the lifetime of the resource, and for issuing samples for research requests once sufficient incident cases of disease have occurred.

- ***Establishment and maintenance of IT systems for participant data:*** The coordinating centre is responsible for establishing information systems and standards for secure storage of all of the participant data from the assessment centres and all of the associated data from the processed and archived participant samples. It will also establish the systems and security for accessing, validation and storage of information from participant health records during long-term follow-up.

- ***General management of UK Biobank as a limited company:*** The coordinating centre is responsible for budgetary and statutory financial control and reporting, management of the central and assessment centre staff, implementation of statutory policies and procedures such as the requirements of the Health and Safety at Work Act.

2.9.5 Ethics & Governance Council

The Ethics & Governance Council (EGC) has been established by the Medical Research Council and the Wellcome Trust in a way that enables it to operate independently of them and of UK Biobank (see www.egcukbiobank.org.uk and Annex 1). The remit of the EGC includes: acting as an independent guardian of the Ethics & Governance Framework and advising the Board on its revision; monitoring and reporting publicly on the conformity of the UK Biobank project with this Framework; and advising more generally on the interests of participants and the general public in relation to UK Biobank. In order to be able to fulfil its remit, the EGC will need to be appropriately

knowledgeable about UK Biobank's continuing activities. It will be able to require from parties involved in UK Biobank whatever information and discussion are necessary to fulfil its remit. Normally the EGC will communicate its reflections and criticism informally. But, if the EGC is not satisfied with UK Biobank's response, it could make a formal statement of concern (e.g. to the Board or funders) or, if necessary, make a public statement that certain actions should or should not be taken. The Ethics & Governance Council will work in an open and transparent fashion and report to participants and the public. This may be achieved in a variety of ways, such as through publishing reports of its reviews or discussions, occasionally meeting in public, or holding public meetings.

2.9.6 International Scientific Advisory Board (ISAB)

The International Scientific Advisory Board (ISAB) has been established by the Medical Research Council and the Wellcome Trust to provide advice to the Principal Investigator, the Board of UK Biobank and the funders on the scientific direction, strategy and operations of the resource (see Annex 1 for membership). It will meet annually to review progress and achievements against the agreed objectives and also the future plans. It will evaluate the outputs of the resource and their contribution to the scientific community both nationally and internationally. Its remit will also include advising and commenting on issues relating to using UK Biobank for collaborative research (such as access to participant data or samples).

Annex 1: UK Biobank committees and staff

UK Biobank Board
Chair: Sir Alan Langlands (University of Dundee)
Vice Chair: Prof. Mike Pringle (University of Nottingham)
Prof John Bell (University of Oxford)
Hon Peter Benson (London)
Ms Jane Lee (Medical Research Council)
Dr Pat Goodwin (Wellcome Trust)
Dr Alison Spaull (Scottish Executive)
Mr C. Marc Taylor (Department of Health)
Secretary: Mr Andrew Moberly

UK Biobank senior staff
Prof Rory Collins (Principal Investigator & Chief Executive)
Dr Tim Peakman (Executive Director)
Dr Tim Sprosen (Chief Scientific Officer)
Mr Steve Walker (Chief Information Officer)
Dr Paul Downey (Head of Laboratories)

UK Biobank Steering Committee
Chair: Prof Rory Collins (University of Oxford)
Prof Valerie Beral (University of Oxford)
Prof Paul Burton (University of Leicester)
Prof Paul Elliott (Imperial College London)
Dr John Gallacher (University of Wales, Cardiff)
Prof Jill Pell (University of Glasgow)
Prof Alan Silman (University of Manchester)
Observers:
Dr Tim Sprosen (UK Biobank)
Dr Tim Peakman (UK Biobank)

Regional Collaborating Consortia (lead institution in italics)
Central England Consortium
University of Oxford

Fosse Way Consortium
University of Leicester
University of Birmingham
Warwick Medical School
University of Nottingham
Peninsula Medical School
University of Sheffield

London Consortium
Imperial College London
University College London
Kings College London
Queen Mary University of London

Welsh Consortium
University of Wales College of Medicine, Cardiff
University of Wales, Swansea
University of Wales, Bangor

Scottish Consortium
University of Glasgow
University of Aberdeen
University of Edinburgh
University of Dundee

North West Wessex Consortium
University of Manchester Medical School
University of Keele Medical School
University of Southampton

International Scientific Advisory Board
Chair: Prof Stephen MacMahon (Sydney University, Australia)
Prof John Danesh (University of Cambridge)
Prof Terry Dwyer (Murdoch Children's Research Institute, Australia)
Dr Silvia Franceschi (International Agency for Research on Cancer, France)
Prof Hilary Graham (University of York)
Dr Tom Hudson (McGill University, Canada)
Dr Prabhat Jha (University of Toronto, Canada)
Prof Bernard Keavney (University of Newcastle)
Prof Michael Kidd (Balmain Hospital Australia)
Prof Mark Lathrop (Centre National de Génotypage, France)
Dr Teri Manolio (National Human Genome Research Institute, USA)
Prof Sir Richard Peto (University of Oxford)
Prof Neil Risch (Stamford University, USA)
Prof Meir Stampfer (Harvard, USA)
Dr Michael Thun (American Cancer Society, USA)

Ethics & Governance Council
Chair: Prof Graeme Laurie
Deputy chair: Ms Andrea Cook OBE
Deputy chair: Prof Roger Higgs
Prof Erica Haimes
Dr Anneke Lucassen
Prof Ian Hughes
Dr Roger Moore
Ms Hilary Newiss
Ms Sally Smith QC
Prof Martin Richards
Dr Heather Widdows
Prof Christopher Wild
Secretary: Ms Adrienne Hunt (Wellcome Trust)

Annex 2.0: References

1. Grimes DA, Schulz KF. Cohort studies: marching towards outcomes. *Lancet* 2002; 359: 341-45.
2. Schulz KF, Grimes DA. Case-control studies: research in reverse. *Lancet* 2002; 359: 431-34.
3. Chen Z, Lee L, Chen J, et al. Cohort Profile: The Kadoorie study of chronic disease in China (KSCDC). *Int J Epi* 2005; 34: 1243-49.
4. Tapia-Conyer R, Kuri-Morales P, Alegre-Diaz J, et al. Cohort profile: The Mexico City Prospective Study. *Int J Epi* 2006; 35: 243-49.
5. Riboli E, Hunt KJ, Slimani N, et al. European Prospective Investigation into Cancer and Nutrition (EPIC): study populations and data collection. *Pub Health Nutr.* 2002; 5: 1113-24.
6. Burton PR, Hansell A. UK Biobank: the expected distribution of incident and prevalent cases of chronic disease and the statistical power of nested case-control studies. *Technical Report for UK Biobank*, 2005. http://www.ukbiobank.ac.uk/
7. Colhoun HM, McKeigue PM, Davey Smith G. Problems of reporting genetic associations with complex outcomes. *Lancet* 2003; 361: 865-72.
8. Lander ES, Kruglyak L. Genetic dissection of complex traits: guidelines for interpreting and reporting linkage results. *Nature Genetics* 1995; 11: 241-47.
9. Todd JA. Interpretation of results from genetic studies of multifactorial diseases. *Lancet* 1999; 354: 15-16.
10. Hattersley AT, McCarthy MI. What makes a good genetic association study. *Lancet* 2006; 366: 1315-23.
11. Garcia-Closas M and Lubin JH (1999) Power and sample size calculations in case-control studies of gene-environment interactions: comments on different designs. *Am J Epi* 1999; 149: 689-92.
12. Marchini J, Cardon LC, Phillips MS, et al. The effects of human population structure on large genetic association studies. *Nature Genetics* 2004; 36: 512-17.
13. Wacholder S, Rothman N, Caporaso N. Counterpoint: Bias from population stratification is not a major threat to the validity of conclusions from epidemiological studies of common polymorphisms and cancer. *Cancer Epidemiology Biomarkers and Prevention* 2002; 11: 513-20.
14. Freedman LF, Reich D, Penney K, et al. Assessing the impact of population stratification on genetic association studies. *Nature Genetics* 2004; 36: 388-93.
15. Centre for Longitudinal studies web site. http://www.cls.ioe.ac.uk/studies.asp?section=000100020003
16. Clarke R, Breeze E, Sherliker P, et al. Design, objectives, and lessons from a pilot 25 year follow up re-survey of survivors in the Whitehall study of London Civil Servants. *J Epi Com Health* 1998; 52: 364-369.
17. McCormick A, Fleming D, Charlton J. Morbidity Statistics from General Practice. Fourth national study 1991-1992. London: HMSO, 1995.
18. Davey Smith G, Ebrahim S, Lewis S, et al. Genetic epidemiology and public health: hope, hype, and future prospects. *Lancet* 2005; 366: 1484-98.
19. Inequalities in health: a report of a research working group. London: Department of Health and Social Security, 1980.
20. Pocock S, Sharper A, Cook D, et al. Social class differences in ischaemic heart disease in British men. *Lancet* 1987; ii: 197-201.
21. Leon D, Wilkinson R. Inequalities in prognosis: socio-economic differences in cancer and heart disease survival. In: Fox J, ed. Health inequalities in European countries. pp. 280-300. Aldershot: Gower, 1989.
22. Morris R, Carstairs V. Which deprivation? A comparison of selected deprivation indexes. *J Public Health Med* 1991; 13: 318-26.
23. Macintyre S, McKay L, Der G, Hiscock R. Socio-economic position and health: what you observe depends on how you measure it. *J Public Health Med* 2003; 25:288-94.
24. Ezzati M, Lopez A, Rodgers A, et al. Selected major risk factors and global and regional burden of disease. *Lancet* 2002; 360: 1347-60.
25. Peto R, Lopez A, Boreham J, et al. Mortality from tobacco in developed countries: indirect estimation from national vital statistics. *Lancet* 1992; 339: 1268-78.

26. Thun MJ, Peto R, Lopez A, et al. Alcohol consumption and mortality among middle-aged and elderly U.S. adults. *N Engl J Med* 1997; 337: 1705-14.
27. Doll R, Peto R, Boreham J, et al. Mortality in relation to smoking: 50 years' observations on male British doctors. *BMJ* 2004; 328: 1519.
28. Feunekes GI, van't Veer P, van Staveren WA, et al. Alcohol intake assessment: the sober facts. *Am J Epi* 1999; 150: 105-12.
29. Barker D, Winter P, Osmond C, et al. Weight in infancy and death from ischaemic heart disease. *Lancet* 1989; ii: 577-80.
30. Weiderpass E, Braaten T, Magnusson C, et al. A prospective study of body size in different periods of life and risk of premenopausal breast cancer. *Cancer Epi Biomarkers Prev* 2004; 13: 1121-27.
31. Troy L, Michels K, Hunter D, et al. Self-reported birthweight and history of having been breastfed among younger women: an assessment of validity. *Int J Epi* 1996; 25: 122-27.
32. Kerber R, Slattery M. Comparison of self-reported and database-linked family history of cancer data in a case-control study. *Am J Epi* 1997; 146: 244-48.
33. Jarvis D, Chinn S, Lucyznska C, et al. Association of respiratory symptoms and lung function in young adults and use of domestic gas appliances. *Lancet* 1996; 347: 426-31.
34. Coggon D, Croft P, Kellingray S, et al. Occupational physical activities and osteoarthritis of the knee. *Arthritis Rheum* 2000; 43: 1443-49.
35. Office of National Statistics. Standard Occupational Classification 2000. London: Crown copyright, 2000.
36. Bingham S, Luben R, Welch A, Wareham NJ, et al. Are imprecise methods obscuring a relation between fat and breast cancer? *Lancet* 2003; 362: 212-14.
37. Beresford SAA, Johnson KC, Ritenbaugh C, et al. Low-fat dietary pattern and risk of colorectal cancer: the Women's Health Initiative Randomized Controlled Dietary Modification Trial. *JAMA* 2006; 295: 643-54.
38. Howard BV, Van Horn L, Hsia J, et al. Low-fat dietary pattern and risk of cardiovascular disease: the Women's Health Initiative Randomized Controlled Dietary Modification Trial. *JAMA* 2006; 295: 655-66.
39. Day NE, McKeown N, Wong MY, et al. Epidemiological assessment of diet: a comparision of a 7-day diary with a food frequency questionnaire using urinary markers of nitrogen, potassium and sodium. *Int J Epi* 2001; 30: 309-17.
40. Willett WC. Commentary: Dietary diaries versus food frequency questionnaires - a case of indigestible data. *Int J Epi* 2001; 30: 317-19.
41. Schatzkin A, Kipnis V, Carroll RJ, et al. A comparison of a food frequency questionnaire with a 24-hour recall for use in an epidemiological cohort study: results from the biomarker-based Observing Protein and Energy Nutrition (OPEN) study. *Int J Epi* 2003; 32: 1054-62.
42. Cade J, Thompson R, Burley V, et al. Development, validation and utilisation of food-frequency questionnaires - a review. *Public Health Nutr* 2002; 5: 567-87.
43. Hoare J, Henderson L, Bates C, et al. The national diet and nutrition survey: adults aged 19 to 64 years. Summary Report. Norwich: Office for National Statistics, 2004.
44. Craig CL, Marshall AL, Sjostrom M, et al. International physical activity questionnaire: 12-country reliability and validity. *Med Sci Sports Exerc* 2003; 35:1 381-95.
45. Hu FB, Li TY, Colditz G, et al. Television watching and other sedentary behaviors in relation to risk of obesity and type 2 diabetes mellitus in women. *JAMA* 2003; 289: 1785-91.
46. Jakes RW, Day NE, Khaw K-T, et al. Television viewing and low participation in vigorous recreation are independently associated with obesity and markers of cardiovascular disease risk: EPIC-Norfolk population-based study. *Eur J Clin Nutr* 2003; 57: 1089-96.
47. Ormel J, Rosmalen J, Farmer A. Neuroticism: a non-informative marker of vulnerability to psychopathology. *Soc Psychiatry Psychiatr Epidemiol* 2004; 39: 906-12.
48. Brayne C, Day N, Gill C. Methodological issues in screening for dementia. *Neuroepi* 1992; 11(suppl 1):88-93.
49. De Jager C, Blackwell AD, Budge MM, et al. Predicting cognitive decline in healthy older adults. *Am J Geriatr Psychiatry* 2005; 13: 735-40.
50. Prospective Studies Collaboration. Age-specific relevance of usual blood pressure to vascular mortality: a meta-analysis of individual data for one million adults in 61 prospective studies. *Lancet* 2002; 360: 1903-13.

51. Qiu C, Winblad B, Fratglioni L. The age-dependent relation of blood pressure to cognitive function and dementia. *Lancet Neurol* 2005; 4: 487-99.
52. Health Survey for England. http://www.dh.gov.uk/PublicationsAndStatistics/0PublishedSurvey/HealthSurveyForEngland/HealthSurveyResults/fs/en. 2004.
53. Clarke R, Shipley M, Lewington S et al. Underestimation of risk associations due to regression dilution in long-term follow-up of prospective studies. *Am J Epi* 1999; 150: 341-53.
54. Webster JD, Hesp R, Garrow JS. The composition of excess weight in obese women estimated by body density, total body water and total body potassium. *Hum Nutr: Clin Nutr* 1984; 38C: 299-306.
55. Willett W. Nutritional epidemiology. Second edition. New York: 1998.
56. World Health Organization. Physical status: the use and interpretation of anthropometry. World Health Organization, 1995.
57. National Task Force on the Prevention and Treatment of Obesity. Overweight, obesity, and health risk: National Task Force on the Prevention and Treatment of Obesity. *Arch Intern Med* 2000; 160: 898-904.
58. Manson JE, Colditz GA, Stampfer MJ et al. A prospective study of obesity and risk of coronary heart disease in women. *N Engl J Med* 1990; 322:882-89.
59. Rexrode KM, Hennekens CH, Willett WC et al. A prospective study of body mass index, weight change, and risk of stroke in women. *JAMA* 1997; 277:1539-45.
60. Carey VJ, Walters EE, Colditz GA et al. Body fat distribution and risk of non-insulin-dependent diabetes mellitus. *Am J Epi* 1997; 145: 614-19.
61. Felson DT, Anderson JJ, Naimark A et al. Obesity and knee osteoarthritis: The Framingham Study. *Ann Intern Med* 1988; 109: 18-24.
62. Calle EE, Rodriguez C, Walker-Thurmond K et al. Overweight, obesity, and mortality from cancer in a prospectively studied cohort of U.S. adults. *N Engl J Med* 2003; 348: 1625-38.
63. IARC Handbooks of Cancer Prevention: Volume 6, Weight Control and Physical Activity. 2002
64. den Tonkelaar I, Seidell JC, Collette HJA. Body fat distribution in relation to breast cancer in women participating in the DOM-project. *Breast Cancer Research and Treatment* 1995; 34: 55-61.
65. Giovannucci E, Ascherio A, Rimm EB, et al. Physical activity, obesity and risk for colon cancer and adenoma in men. *Ann Intern Med* 1995; 122: 327-34.
66. Wannamethee SG, Shaper AG, Whincup PH et al. Adult height, stroke, and coronary heart disease. *Am J Epi* 1998; 148: 1069-76.
67. Batty GD, Shipley MJ, Langenberg C et al. Adult height in relation to mortality from 14 cancer sites in men in London (UK): evidence from the original Whitehall study. *Ann Oncology* 2006; 17: 157-66.
68. Hardman AE. Physical activity, obesity and blood lipids. *Int J Obesity Related Metabolic Disorders* 1999; 23: S64-71.
69. Rexrode KM, Carey VJ, Hennekens CH et al. Abdominal adiposity and coronary heart disease in women. *JAMA* 1998; 280: 1843-48.
70. Kahn BB, Flier JS. Obesity and insulin resistance. *J Clin Investigation* 2000; 106: 473-81.
71. Wang Y, Rimm EB, Stampfer MJ et al. Comparison of abdominal obesity and overall obesity in predicting risk of type 2 diabetes among men. *Am J Clin Nutr* 2005; 81: 555-63.
72. Baik I, Ascherio A, Rimm EB et al. Adiposity and mortality in men. *Am J Epi* 2000; 152: 264-71.
73. Janssen I, et al. Waist circumference and not body mass index explains obesity-related health risk. *Am J Clin Nutr* 2004; 79: 379-84.
74. Ribeiro-Filho FF, Faria AN, Azjen S et al. Methods of estimation of visceral fat: Advantages of ultrasonography. *Obesity Research* 2003; 11: 94.
75. Schreiner PJ, et al. Sex-specific associations of magnetic resonance imaging-derived intra-abdominal and subcutaneous fat areas with conventional anthropometric indices: The Atherosclerosis Risk in Communities Study. *Am J Epi* 1996; 144: 335-45.
76. Molarius A, Seidell JC, Sans S et al. Waist and hip circumferences, and waist-hip ratio in 19 populations of the WHO MONICA Project. *Int J Obesity* 1999; 23: 116-25.

77. Caulfield M, Munroe P, Pembroke J, et al. Genome-wide mapping of human loci for essential hypertension. *Lancet* 2003; 361: 2118-23.
78. Heitmann BL, Frederiksen P, Lissner L. Hip circumference and cardiovascular morbidity and mortality in men and women. *Obesity Research* 2004; 12: 482-7.
79. Seidell JC, Pérusse L, Després J-P et al. Waist and hip circumferences have independent and opposite effects on cardiovascular disease risk factors: the Quebec Family Study. *Am J Clin Nutr* 2001; 74: 315-21.
80. Yusuf S, Hawken S, Ôunpuu S et al. Obesity and the risk of myocardial infarction in 27 000 participants from 52 countries: a case-control study. *Lancet* 2005; 366: 1640-9.
81. National Institutes of Health. Clinical guidelines on the identification, evaluation, and treatment of overweight and obesity in adults - the evidence report. *Obesity Research* 1998; 6(Suppl 2): 51S-209S.
82. Willett WC, Dietz WH, Colditz GA. Guidelines for healthy weight. *N Engl J Med* 1999; 341: 427-34.
83. Deurenberg P, Deurenberg-Yap M. Validation of skinfold thickness and hand-held impedance measurements for estimation of body fat percentage among Singaporean Chinese, Malay and Indian subjects. *Asia Pacific J Clin Nutr* 2002; 11: 1-7.
84. Jebb SA, Cole TJ, Doman D et al. Evaluation of the novel Tanita body-fat analyser to measure body composition by comparison with a four-compartment model. *Brit Jf Nutr* 2000; 83: 115-22.
85. Prentice AM, Jebb SA. Beyond body mass index. *Obesity Reviews* 2001; 31:1-7.
86. Ellis K. Human body composition: In vivo methods. *Physiological Reviews* 2000; 80:649-80.
87. Sternfeld B, Nogo L, Satariano WA et al. Associations of body composition with physical performance and self-reported functional limitation in elderly men and women. *Am J Epi* 2002; 156: 110-21.
88. Luke A, Ramón D, Rotimi C et al. Relation between body mass index and body fat in black population samples from Nigeria, Jamaica, and the United States. *Am J Epi* 1997; 145: 620-28.
89. Harris T. Invited commentary: Body composition in studies of aging: New opportunities to better understand health risks associated with weight. *Am J Epi* 2002; 156: 122-24.
90. Rantanen T, Harris T, Leveille SG, et al. Muscle strength and body mass index as long term predictors of mortality in initially healthy men. *J Gerontology Series A Biological Sciences and Medical Sciences* 2000; 55A: M168-73.
91. Rantanen T, Guralnik JM, Foley D, et al. Midlife hand grip strength as a predictor of old age disability. *JAMA* 1999; 281: 558-60.
92. Rantanen T, Volpato S, Ferrucci L, et al. Hand grip strength and cause-specific and total mortality in older disabled women: exploring the mechanism. *J Am Geriatrics Society* 2003; 51: 636-41.
93. Metter EJ, Talbot LA, Schrager M, et al. Skeletal muscle strength as a predictor of all-cause mortality in healthy men. *J Gerontology Series A Biological Sciences and Medical Sciences* 2002; 57A: B359-65.
94. Dixon WG, Lunt M, Pye SR, et al. Low grip strength is associated with bone mineral density and vertebral fracture in women. *Rheumatology* 2005; 44: 642-46.
95. Ebi-Kryston KL. Respiratory symptoms and pulmonary function as predictors of 10-year mortality from respiratory disease, cardiovascular disease and all causes in the Whitehall Study. *J Clin Epi* 1988; 41: 251-60.
96. Truelsen T, Prescott E, Lange P, et al. Lung function and risk of fatal and non-fatal stroke. The Copenhagen City Heart Study. *Int J Epi* 2001; 30: 145-51.
97. Sin DD, Wu LL, Man SFP. The relationship between reduced lung function and cardiovascular mortality: a population-based study and a systematic review of the literature. *Chest* 2005; 127: 1952-59.
98. Strachan DP. Ventilatory function, height and mortality among lifelong non-smokers. *J Epi Comm Health* 1992; 46: 66-70.
99. Canoy D, Luben R, Welsh A, et al. Abdominal obesity and respiratory function in men and women in the EPIC-Norfolk study, United Kingdom. *Am J Epi* 2004; 159: 1140-49.
100. Myint PK, Luben RN, Surtees PG, et al. Respiratory function and self-reported functional health: EPIC-Norfolk population study. *Eur Resp J* 2005; 26: 494-502.

101. Hemingway H, Shipley M, Britton A, et al. Prognosis of angina with and without a diagnosis: 11-year follow-up in the Whitehall II prospective cohort study. *BMJ* 2003; 327: 895-98.
102. Whincup PH, Wannamethee G, Macfarlane PW, et al. Resting electrocardiogram and risk of coronary heart disease in middle-aged British men. *J Cardiovasc Risk* 1995; 2: 533-43.
103. Hans D, Dargent-Molina P, Schott AM, et al. Ultrasonographic heel measurements to predict hip fracture in elderly women: The EPIDOS prospective study. *Lancet* 1996; 348: 511-14.
104. Khaw K-T, Reeve J, Luben R, et al. Prediction of total and hip fracture risk in men and women by quantitative ultrasound of the calcaneous: EPIC-Norfolk prospective population study. *Lancet* 2004; 363: 197-202.
105. UK Biobank sample handling pilots. *Int J Epi* 2006 (in press)
106. Cranfield Centre for Analytical Science – Biomarkers in IBS www.cranfield.ac.uk/ibst/ccas/research/medical/biomarkers.htm
107. Nielsen JB, Andersen O, Grandjean P. Evaluation of mercury in hair, blood and muscle as biomarkers for methylmercury exposure in male and female mice. *Arch Toxicol* 1994; 68: 317-21.
108. Vahter M, Marafante E, Dencker L. Metabolism of arsenobetaine in mice, rats and rabbits. *Sci Total Environ* 1983; 30: 197-211.
109. Harrington JM, Middaugh DL, Housworth J. A survey of a population exposed to high concentrations of arsenic in well water in Fairbanks, Alaska. *Am J Epi* 1978; 108: 377-85.
110. Landi MT, Caporaso N. Sample collection, processing and storage. *IARC Sci Publ* 1997; 142: 223-36.
111. Kohek M, Leme C, Nak I, et al. Effects of EDTA and Sodium Citrate on hormone measurements by fluorometric (FIA) and immunofluorometric (IFMA) methods. *BMC Clin Path* 2002; 2:2. www.biomedcentral.com/1472-6890/2/2/
112. House RV. Cytokine measurement techniques for assessing hypersensitivity. *Toxicol* 2001; 158:51-58.
113. The UK Biobank protocol: A study of genes, environment and health. February 2002.
114. MacMahon S, Peto R, Cutler J, et al. Blood pressure, stroke, and coronary heart disease. Part 1, Prolonged differences in blood pressure: prospective observational studies corrected for the regression dilution bias. *Lancet* 1990; 335: 765-74.
115. Kaaks R, Riboli E. Validation and calibration of dietary intake measurements in the EPIC project: methodological considerations. *Int J Epi* 1997; 26 S15-25.
116. Wareham NJ, Jakes RW, Rennie KL, Schuit J, Mitchell J, Hennings S, Day NE. Validity and repeatability of a simple index derived from the short physical activity questionnaire used in the European Prospective Investigation into Cancer and Nutrition (EPIC) study. *Pub Health Nutr*. 2003; 6: 407-13.
117. Ceesay SM, Prentice AM, Day KC, et al. The use of heart rate monitoring in the estimation of energy expenditure: a validation study using indirect whole-body calorimetry. *Br J Nutr* 1989; 61: 175-86.
118. Phillips AN, Davey Smith G. The design of prospective epidemiological studies: more subjects or better measurements? *J Clin Epi* 1993; 46: 1203-11
119. Wong MY, Day NE, Luan JA, et al. The detection of gene-environment interaction for continuous traits: should we deal with measurement error by bigger studies or better measurement? *Int J Epi* 2003; 32: 51-57
120. Heart Protection Study Collaborative Group. MRC/BHF Heart Protection Study of cholesterol lowering with simvastatin in 20,536 high-risk individuals: a randomised placebo-controlled trial. *Lancet* 2002; 360: 7-22.
121. National Programme for IT in the NHS – NHS Connecting for Health www.connectingforhealth.nhs.uk
122. The Million Women Study Collaborative Group. The Million Women Study: design and characteristics of the study population. *Breast Cancer Res* 1999; 1: 73-80.
123. Department of Health. Primary Care Computing. URL: http://www.d.gov.uk/PolicyAndGuidance/OrganisationPolicy/PrimaryCare/PrimaryCareComputing/fs/en
124. Mitchell E, Smith G. An oral history of everyday general practice 9: Record keepers. *British J Gen Pract* 2003; 53: 166-7.

125. Hippisley-Cox J, Pringle M, Cater R, et al. Electronic record in primary care—regression or progression? Cross sectional survey. *BMJ* 2003; 326: 1439-43.
126. Information Services Division Scotland Quality & Outcomes Framework www.isdscotland.org/isd/info3.jsp?pContentID=3305&p_applic=CCC&p_service=Content.show&
127. Hobbs FDR, Hawker A. Computerised data collection: practicability and quality in selected general practices. *Fam Pract* 1995; 12: 221-6.
128. Pringle M, Ward P, Chivers C. Assessment of the completeness and accuracy of computer medical records in four practices committed to recording data on computer. *Br J Gen Pract* 1995; 45: 537-41.
129. Thiru K, de Lusignan S, Hague N. Have the completeness and accuracy of computer medical records in general practice improved in the last five years? The report of a two-practice pilot study. *Health Informatics Journal* 1999:5(4); 233-239.
130. Hassey A, Gerrett D, Wilson A. A survey of validity and utility of electronic patient records in a general practice. *BMJ* 2001; 322: 1401-1405.
131. Stearns MQ, Price C, Spackman KA, et al. SNOMED clinical terms: overview of the development process and project status. In: Bakken S, ed. Proceedings of the 2001 AMIA Fall Symposium. Philadelphia: Hanley and Belfus, 2001: 662-6.
132. ISD Scotland. www.isdscotland.org/isd/collect2.jsp?p_applic=CCC&p_service=Content.show&pContentID=785
